# A ‘train the trainers’ approach to infection prevention and control training in pandemic conditions

**DOI:** 10.1016/j.clinpr.2023.100228

**Published:** 2023-07

**Authors:** Kathryn A. Haigh, Francesca Liuzzi, Sharon Irvine, Alison Thompson, Edward Hepworth, Marie-Claire Hoyle, James Cruise, Paul Hine, Naomi F. Walker

**Affiliations:** a Tropical and Infectious Diseases Unit, Liverpool University Hospitals NHS Trust, Prescot Street, Liverpool L7 8XP, UK; b Institute of Infection, Veterinary and Ecological Sciences, University of Liverpool, Liverpool L69 3BX, UK; c Infection Prevention and Control, Liverpool University Hospitals NHS Trust, Prescot Street, Liverpool L7 8XP, UK; d Department of Clinical Sciences, Liverpool School of Tropical Medicine, Pembroke Place, Liverpool L3 5QA, UK

**Keywords:** IPC, COVID-19, Training, Pandemic, Infection, Education

## Abstract

**Background:**

The first wave of the SARS-CoV-2 global pandemic in early 2020 required a rapid roll-out of infection prevention and control (IPC) training for healthcare workers (HCW), including use of appropriate personal protective equipment (PPE). Education about respiratory droplet and aerosol transmission was of paramount importance to ensure safe working practices and improve confidence.

**Methods:**

A joint working group of Infectious Diseases and IPC staff developed a ‘train the trainers’ programme, to be rapidly deployed over a three-week period. This model utilised a snowballing approach, training selected staff with the intention that they would train their teams, facilitating swift cascading of information. Targeted invitations prompted staff from diverse departments of the hospital to attend. Pre- and post-session questionnaires evaluated staff confidence with regard to appropriate PPE use.

**Results:**

The programme trained 130 HCW over a three week period, was well received and led to increased confidence with PPE use amongst staff. Real-time evaluation ensured content could be adapted to the specific needs of HCW involved. We highlight perceived gaps in training despite existing and enhanced training structures.

**Conclusion:**

Provision of face-to-face training in transmission-based precautions, including PPE use, is required to maintain confidence in safe and appropriate IPC amongst hospital staff. We highlight the importance of including non-clinical staff in PPE educational programmes, recognising that these roles are vital for patient care and are frequently patient-facing. We recommend adopting the train the trainers model to facilitate rapid dissemination of education, with interactive multidisciplinary training in future outbreaks to improve HCW confidence and effective IPC.

## Highlights

• The SARS-CoV-2 pandemic has highlighted the importance of IPC training.
• A train the trainers approach was implemented successfully in a large NHS Trust.
• Face-to-face training is recommended to maintain confidence in PPE use in staff.
• Non-clinical staff benefit from inclusion in PPE educational programmes.
• Interdisciplinary team working is vital for successful rapid roll-out of training.

## Background

Severe acute respiratory syndrome coronavirus 2 (SARS-CoV-2), responsible for the current global COVID-19 pandemic, is a virulent pathogen spread by respiratory droplets and aerosols. Over 520 million people have been infected, with a mortality of approximately 1.2% (Johns Hopkins University and Medicine, 2020). During the first surge of COVID-19 in the United Kingdom (UK) between March and May 2020, care of COVID-19 patients transitioned from specialist care (Infectious Diseases (ID) and Intensive Care) to acute and general medical settings, as SARS-CoV-2 was declassified from a high consequence infectious disease (Health Security, 2020). Guidelines from the national public health authority, then called Public Health England (PHE), now named the UK Health Security Agency (UKHSA), on personal protective equipment (PPE) use for COVID-19 were published and revised on several occasions (Health Security, 2020). Due to the rapid rise in case numbers, preparation and training of frontline healthcare workers (HCW) in the National Health Service (NHS) in appropriate use of PPE was limited. Owing to high observed mortality and uncertainty about transmissibility, there was considerable apprehension amongst the workforce (Wingfield and Taegtmeyer, 2020).

At the Royal Liverpool University Hospital, a large tertiary teaching hospital in North West England, all staff routinely receive annual training on infection prevention and control (IPC). With the onset of the SARS-CoV-2 pandemic, PHE guidelines and training materials were disseminated to staff via email and the intranet. The IPC team provided support by visiting wards and advising managers. The role of ‘PPE advocate’ was established, comprising staff from diverse backgrounds, including veterinary surgeons, dental technicians and administrative staff, to encourage appropriate PPE use at ward entry and exit points. Despite this, great variability in PPE use was observed. Common errors comprised recycling gowns, re-using face masks and simultaneous use of multiple aprons or pairs of gloves.

A joint working group from ID and IPC teams recognised an urgent need for enhanced PPE training. The rapidly evolving nature of the situation and changing guidelines was causing confusion amongst staff, negatively impacting PPE use. With insufficient personnel to individually train all 7,500 staff employed at the hospital in a timely fashion, a ‘train the trainers’ approach was adopted to meet this need. This model utilises a snowballing approach, training key staff who then train others within their teams, rapidly cascading information to large numbers. Train the trainers models have been used successfully for other purposes (Centers for Disease Control and Prevention, 2020, Olayo et al., 2019). Training was implemented and evaluated concurrently, to inform and improve the process, and ensure it met the needs of participants. The programme built staff confidence and supported implementation of effective IPC practice in the hospital, as shown by the results of surveys evaluating PPE training coverage and staff confidence levels.

## Methods

### Hospital surveys

Surveys were carried out before (March 2020) and after (September 2020) the training programme (April-May 2020). The initial survey established the need for enhanced training, the repeat survey assessed whether the intervention had been effective. Surveys utilised convenience sampling of medical, nursing, allied healthcare professionals, portering, domestic and support staff. Fifteen wards were surveyed and a minimum of two staff per ward were asked their job role, whether they had received training in PPE and how confident they were using PPE. All staff provided consent and data were anonymised. There was no attempt to sample the same staff members in the repeat survey. The results were compared using the Chi-squared test.

### Pilot session and development of teaching material

Teaching material for the train the trainers programme was developed by members of the ID and IPC working group, based on the Trust SARS CoV-2 IPC policy. Content for a ‘pilot’ training session was based on perceived and observed need. The session included demonstrations and practical experience with PPE but revealed the need for additional content, including adaptation of PPE protocols to specific areas within the hospital (for example, the Resuscitation area of the Emergency Department). Questions raised by participants highlighted areas of uncertainty, such as evidence regarding transmissibility of SARS-CoV-2. Following debrief and discussion within the team, training materials were revised to address these issues.

The final two-hour teaching programme comprised the following sections (accompanied by a PowerPoint presentation): (1) SARS-CoV-2 background and basic virology; (2) discussion around the chain of infection; (3) demonstration of donning and doffing PPE for aerosol-generating and non-aerosol generating procedures including the processes for side rooms and bays; (4) advice around communication challenges. The slide set template can be found in the Supplementary Appendix (online). Each session was led by two demonstrators from the ID and IPC departments. Participants filled in anonymised pre- and post-session questionnaires assessing confidence with PPE use, scored from 1 (no confidence) to 10 (completely confident). Pre- and post-training scores were compared by Wilcoxon rank-sum test. Qualitative feedback was collected in free text.

### Train the trainers

Staff were invited using a targeted approach, which aimed to train staff who were influential in their wards or areas. Thus, senior nurses, Infectious Diseases doctors, medical registrars and PPE advocates were amongst the first to be invited. Staff from portering, cleaning and catering teams were also invited. These groups may be overlooked by ward-based approaches to training, but frequently have as much interaction with potentially infectious patients as clinical staff.

The work was undertaken as a Quality Improvement Project with Trust Governance approval, following a Plan-Do-Study-Act cycle approach (Improving, 2014). Statistical analysis was performed using R version 3.6.1 (R Foundation for Statistical Computing, Vienna, Austria).

## Results

### Initial hospital survey

The survey (March 2020) was completed by 63 staff members from 14 wards plus non-ward-based staff, for example porters. Inpatients in all but one of these wards had confirmed or queried COVID-19 at the time. Despite the available guidance, mandatory training and IPC support, 28/63 (44%) respondents reported no training in PPE use.

### Training attendance

**Fig. 1:**
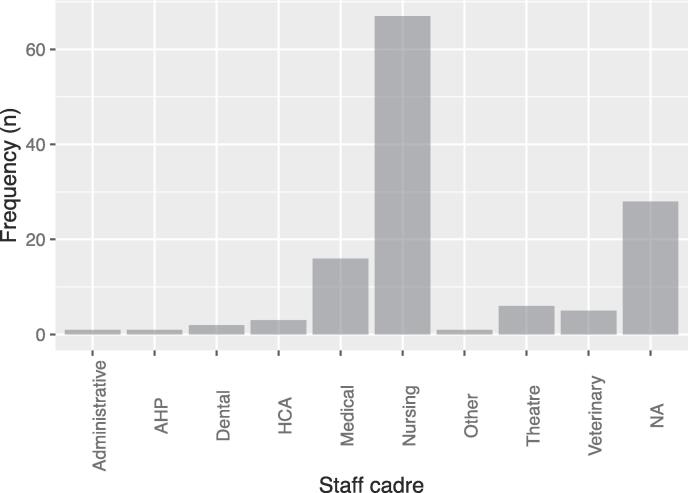
Cadres of staff attending training. AHP = allied healthcare professional, HCA = healthcare assistant, NA = field not completed.

Between 27th April to 20th May 2020, 130 staff were trained over 16 sessions. A wide range of HCWs attended, including medical, nursing, healthcare assistant, allied healthcare professional, dental, administrative, portering, cleaning and catering staff (Fig. 1).

### Pre- and post-session questionnaires

**Fig. 2:**
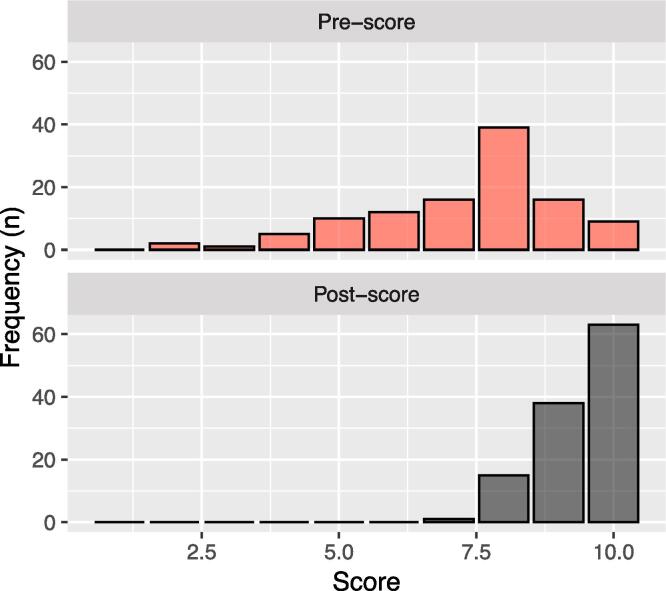
Distribution of confidence scores pre- and post-training.

Pre-session questionnaires were completed by 110/130 participants (Fig. 2). On a 1–10 confidence scale (higher score representing more confidence) regarding PPE use, the median score was 8 and the range was 2–10, with 23% of respondents reporting a confidence score of 9 or 10. Post-session questionnaires, containing the same question template, were completed by 117/130 participants. These revealed a median score of 10 and range 7–10, with 86% of respondents reporting a confidence score of 9 or 10. The difference in these scores did not meet statistical significance (Wilcoxon rank sum test, p = 0.14). However, with no prospective plan for statistical analysis, the study was not powered to detect a difference. Pre- and post-session scores were not matched by participant. All participants were given the opportunity to complete the questionnaires but some chose not to, resulting in missing data.

### Qualitative feedback

Free text comments were provided by 68 respondents. Feedback was collated and reviewed after each session. Comments were globally positive, although one participant commented that the intervention came ‘unfortunately late in the pandemic’. Some staff members reported they were not always positively received when trying to advise others on correct PPE use and found the communication section particularly beneficial for this purpose.

### Repeat hospital survey

The repeat survey (September 2020) was completed by 77 hospital staff members from 16 wards plus non-assigned staff. At this time, 17/77 (22%) respondents reported no training in PPE use. Both surveys captured medical, nursing, allied healthcare professionals, portering, domestic and support staff. The difference in reported training was significant compared to the initial survey (Chi-squared, p < 0.01).

## Discussion

Implementation and evaluation of an enhanced PPE training programme was achieved using a train the trainer model during the first wave of the SARS-CoV-2 pandemic. The programme increased coverage of PPE training and improved confidence with PPE use.

### Defining the need for training

That 44% of respondents to the initial survey reported no training in PPE use was surprising given the availability of training resources. However, the survey reflected the subjective experience of the working group that training was not being accessed, at a time when PPE competence was critical to prevent HCW infections. Respondents comprised a wide range of staff working across medical and surgical wards, many of whom were caring for patients with COVID-19 infection. Given the rapidly increasing numbers of SARS-CoV-2 cases in the Trust, and lack of other control measures at the time (e.g. vaccination), this clearly identified the need for enhanced training.

### Logistical challenges

Securing suitable venues with the necessary facilities and space to maintain social distancing was a challenge. Scheduling ID and IPC personnel to be available to run sessions concurrently was also difficult due to demands on their time. However, the importance of training was recognised and staff members were freed up from clinical duties where required.

### Self-reported confidence with PPE use

The pre- and post-session questionnaire results indicate a positive effect from the training. High baseline confidence in PPE use likely reflects that staff in leadership roles were specifically targeted. However, despite reported confidence, considerable uncertainty and variable practice was observed. Qualitative feedback showed participants felt their confidence had increased after the session.

### See one, do one, teach one

Many attendees informally reported back that they had been training in their own areas and seen improvements in PPE use amongst colleagues. Participants were also observed delivering teaching to other staff members in their teams and displaying confidence doing so.

### Impact on PPE use

Following this programme, PPE use around the Trust improved. Common errors, such as wearing two aprons concurrently, were no longer observed. One session was recorded for future use in online training for Trust staff.

The second survey carried out four months after the intervention showed 22% of staff reported no training in PPE use. Whilst this highlighted an ongoing training gap, coverage had improved compared to the first survey (44%), with the difference reaching statistical significance. An 100% target was felt to be difficult to achieve as agency and temporary staff may not have ready access to training. However, as case numbers lessened, training may have been deprioritised. Prior to the second wave of cases, Trust management established a new ‘PPE advisory group’ and the training materials devised have been of ongoing use.

## Limitations

Quantitative data on how many HCWs each new trainer went on to train would have helped assess the impact of the approach, however this was not feasible at the time due to human resource constraints. Convenience sampling introduces a risk of selection bias. To mitigate against this, both surveys were carried out in a similar fashion with similar staff cadres of respondents. Employing a targeted approach to invite participants contributes a further risk of selection bias. However, given the clear need to rapidly disseminate information, we felt that inviting staff perceived to be influential was appropriate. Paired responses would improve statistical analysis, however forms were not paired to maintain confidentiality and for ease of running sessions. Participants were not obliged to complete the feedback questionnaires, which has led to missing data. The competency of those receiving training was not assessed, however formal assessment was not possible due to staffing pressures. IPC/ID staff formally assessed competence at the end of the pilot session and saw significant improvement.

## Conclusion

This article demonstrates how a train the trainers approach facilitated rapid IPC training throughout an NHS Hospital Trust in a pandemic context. Interdisciplinary team working, real time evaluation and the inclusion of all cadres of staff, including those in non-clinical roles, were key factors in the success of the programme. Additional work with train the trainers models, incorporating formal competence assessments, would help to further validate this approach. IPC training, whilst particularly pertinent in the midst of a global SARS-CoV-2 pandemic, is always relevant in prevention of nosocomial infection from other infectious pathogens. The results of this intervention show that, despite enhanced IPC education, training gaps persisted, demonstrating the challenge that IPC teams face in maintaining competencies amongst staff, especially in the face of evolving guidance. This model is recommended to facilitate rapid dissemination of IPC training in pandemic conditions.

## CRediT authorship contribution statement

**Kathryn A. Haigh:** Conceptualization, Methodology, Formal analysis, Investigation, Writing – original draft. **Francesca Liuzzi:** Investigation, Writing – review & editing. **Sharon Irvine:** Conceptualization, Methodology, Investigation, Writing – review & editing, Supervision. **Alison Thompson:** Conceptualization, Investigation, Writing – review & editing. **Edward Hepworth:** Investigation, Writing – review & editing. **Marie-Claire Hoyle:** Conceptualization, Methodology, Investigation, Writing – review & editing. **James Cruise:** Investigation, Writing – review & editing. **Paul Hine:** Methodology, Investigation, Writing – review & editing. **Naomi F. Walker:** Conceptualization, Methodology, Investigation, Writing – review & editing, Supervision.

## Declaration of Competing Interest

The authors declare that they have no known competing financial interests or personal relationships that could have appeared to influence the work reported in this paper.
