# Supplementary material for: A ‘train the trainers’ approach to infection prevention and control training in pandemic conditions
Source: Clin Infect Pract. 2023 Jul;19:100228. doi: 10.1016/j.clinpr.2023.100228 (PMC10148243; doi:10.1016/j.clinpr.2023.100228)

## Slide 1
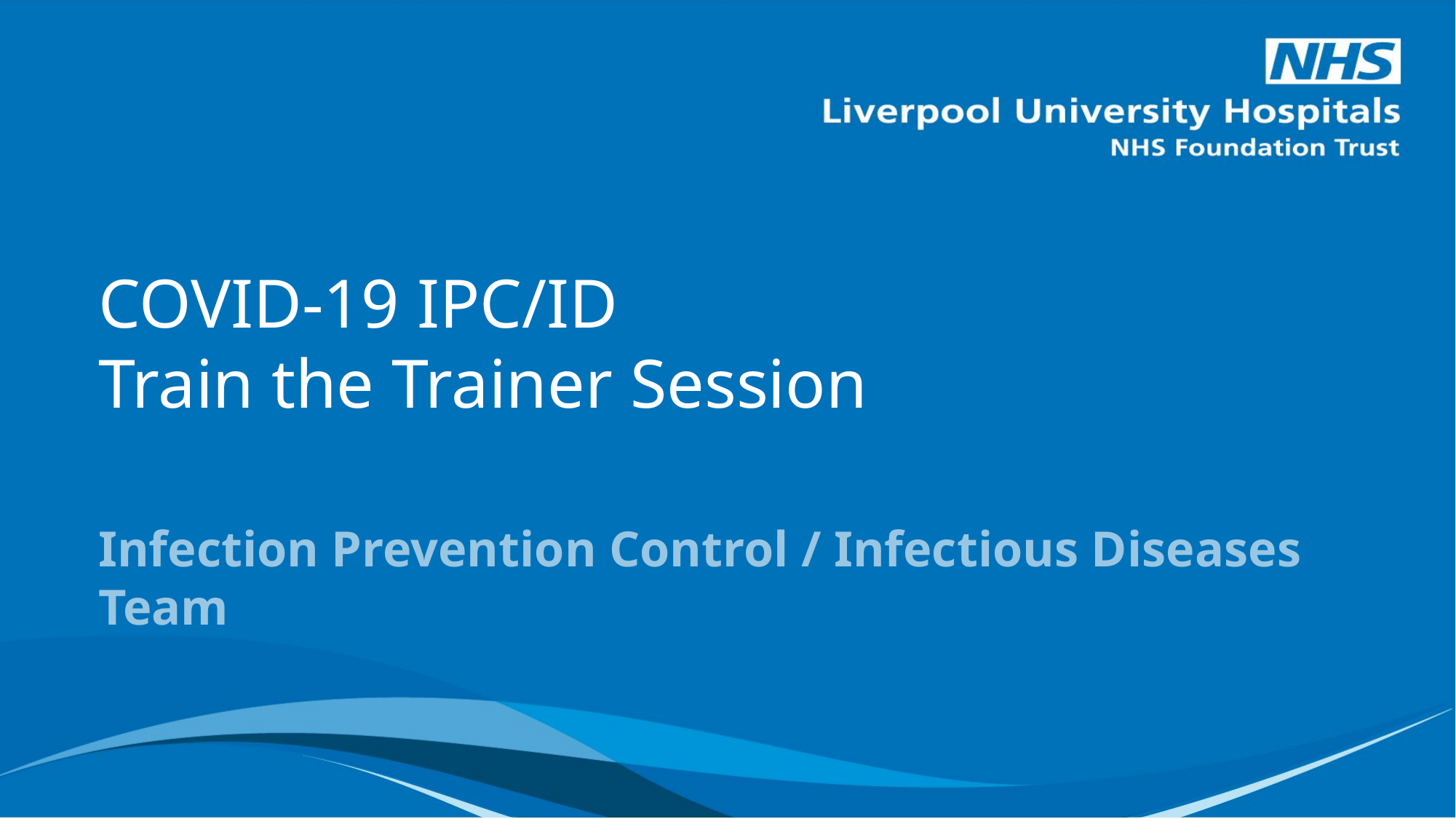

# COVID-19 IPC/ID Train the Trainer Session
Infection Prevention Control / Infectious Diseases Team

## Slide 2
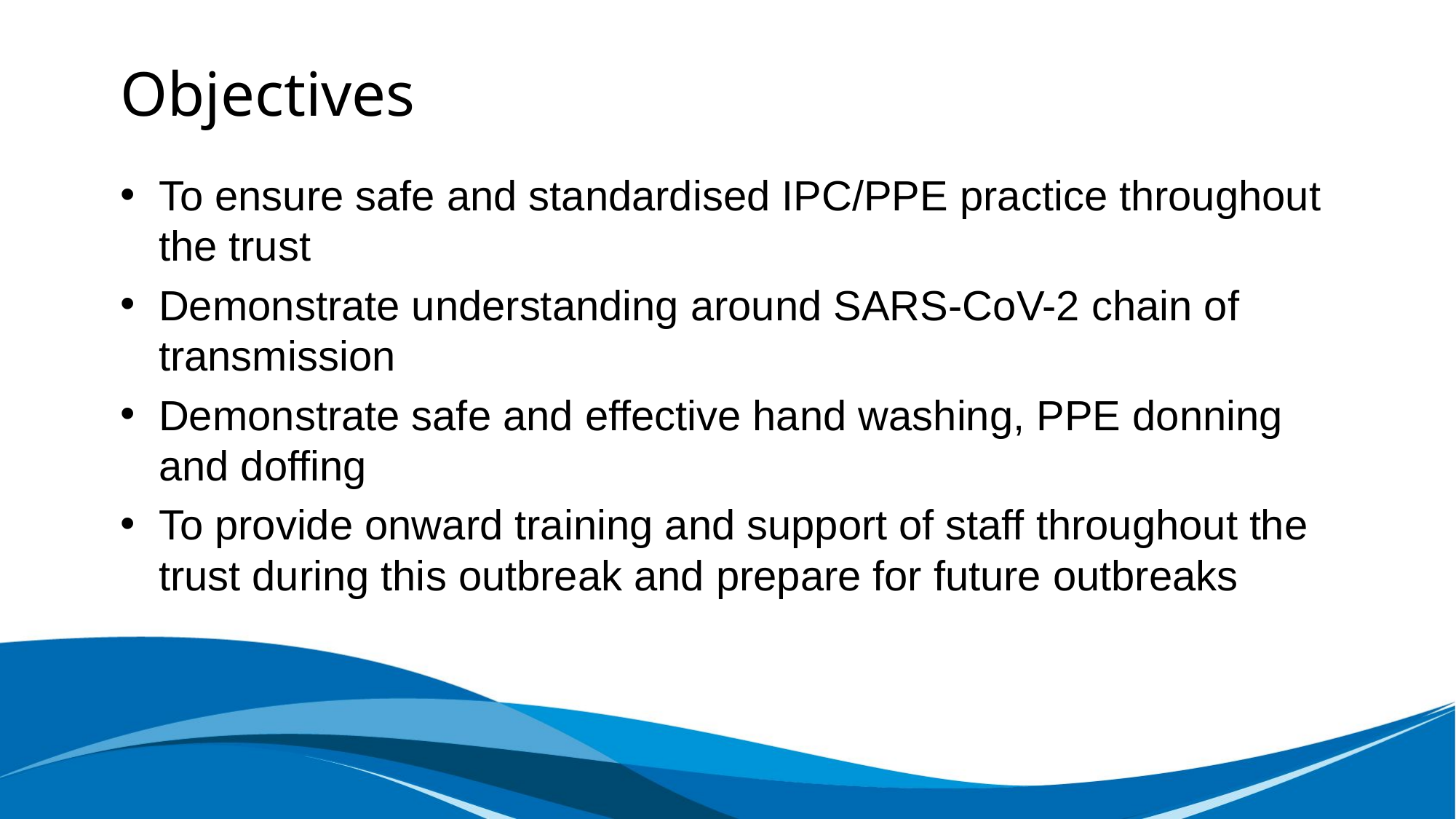

Objectives
To ensure safe and standardised IPC/PPE practice throughout the trust
Demonstrate understanding around SARS-CoV-2 chain of transmission
Demonstrate safe and effective hand washing, PPE donning and doffing
To provide onward training and support of staff throughout the trust during this outbreak and prepare for future outbreaks

## Slide 3
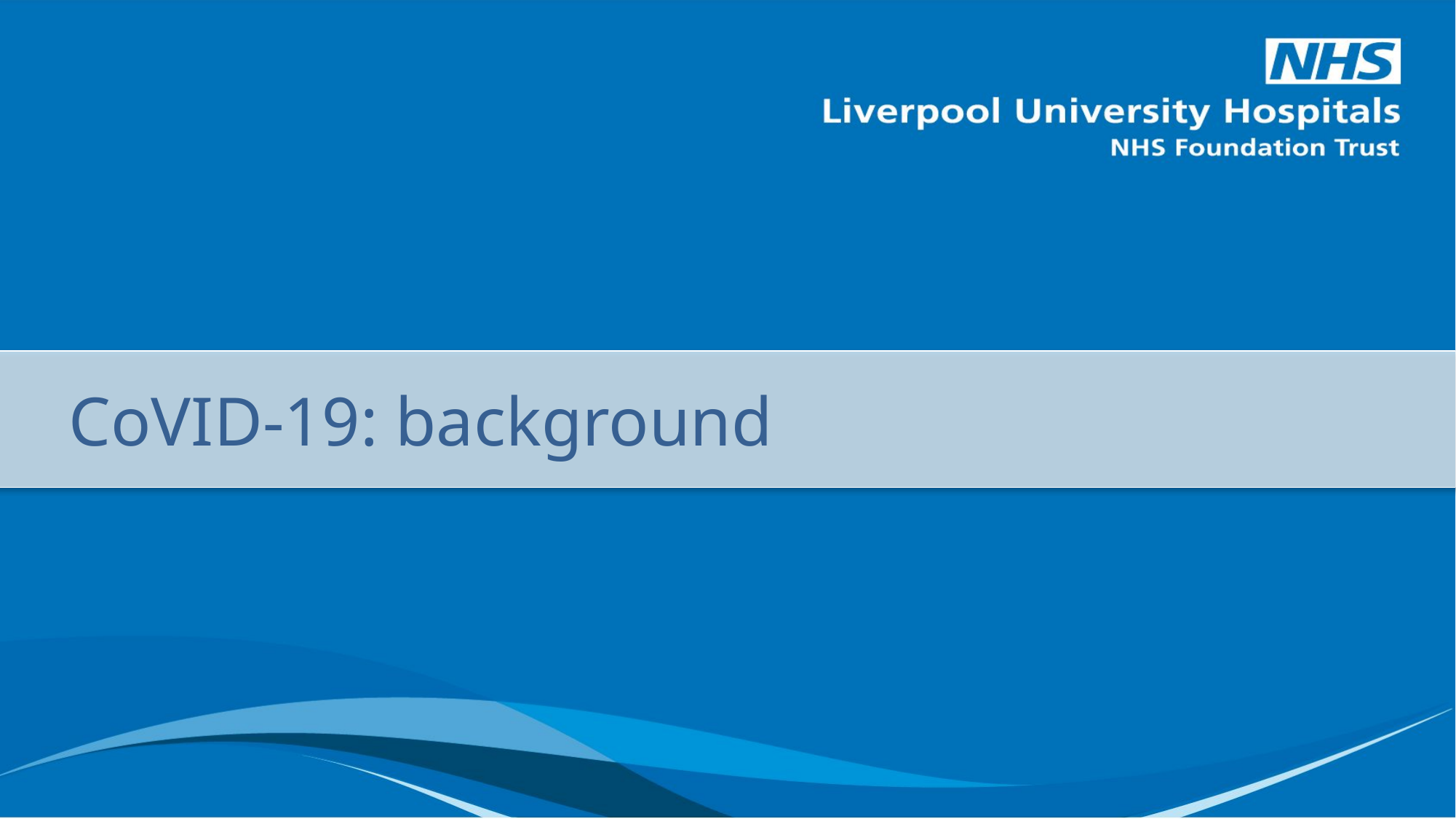

# CoVID-19: background

## Slide 4
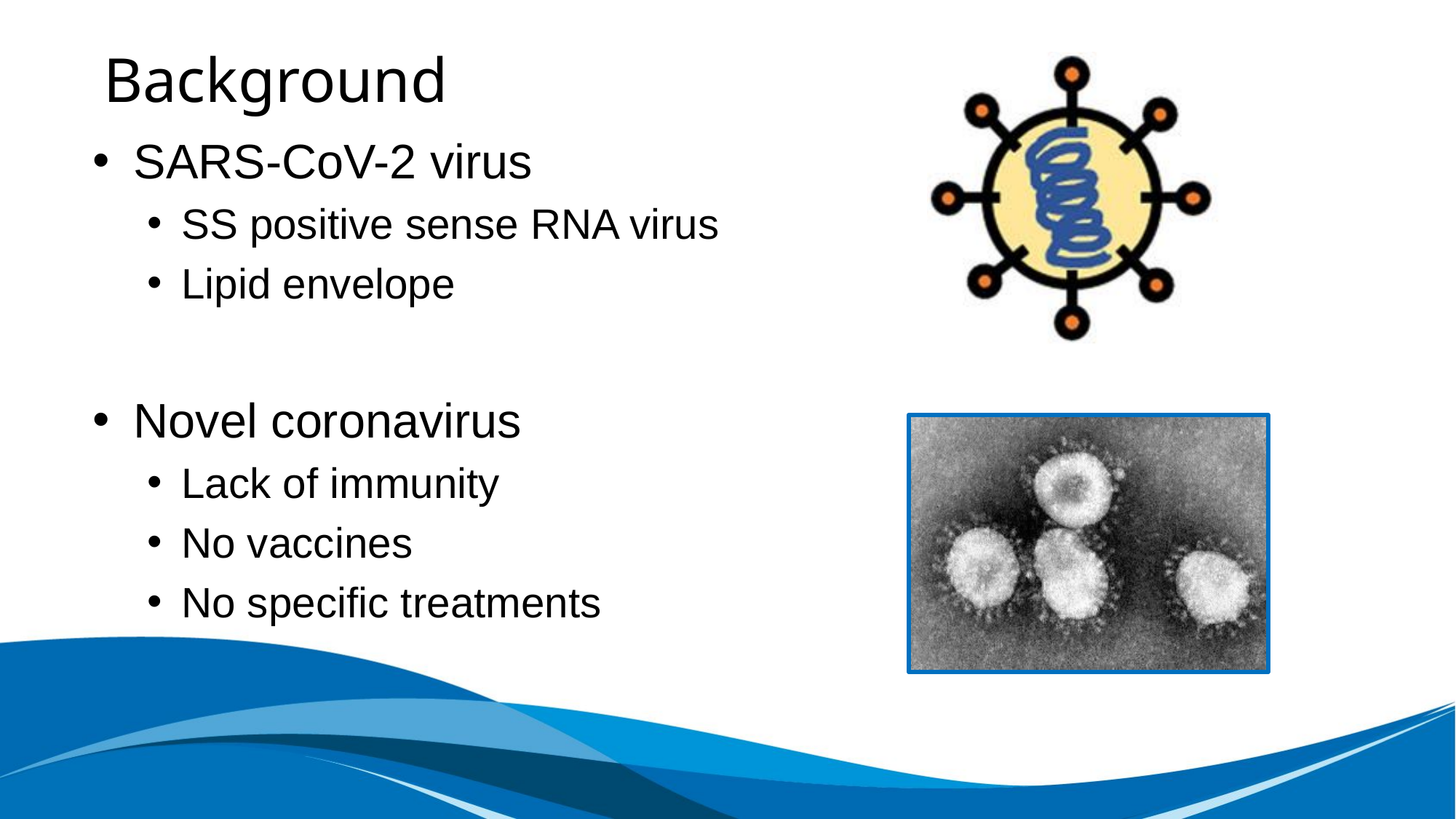

Background
SARS-CoV-2 virus
SS positive sense RNA virus
Lipid envelope
Novel coronavirus
Lack of immunity
No vaccines
No specific treatments

## Slide 5
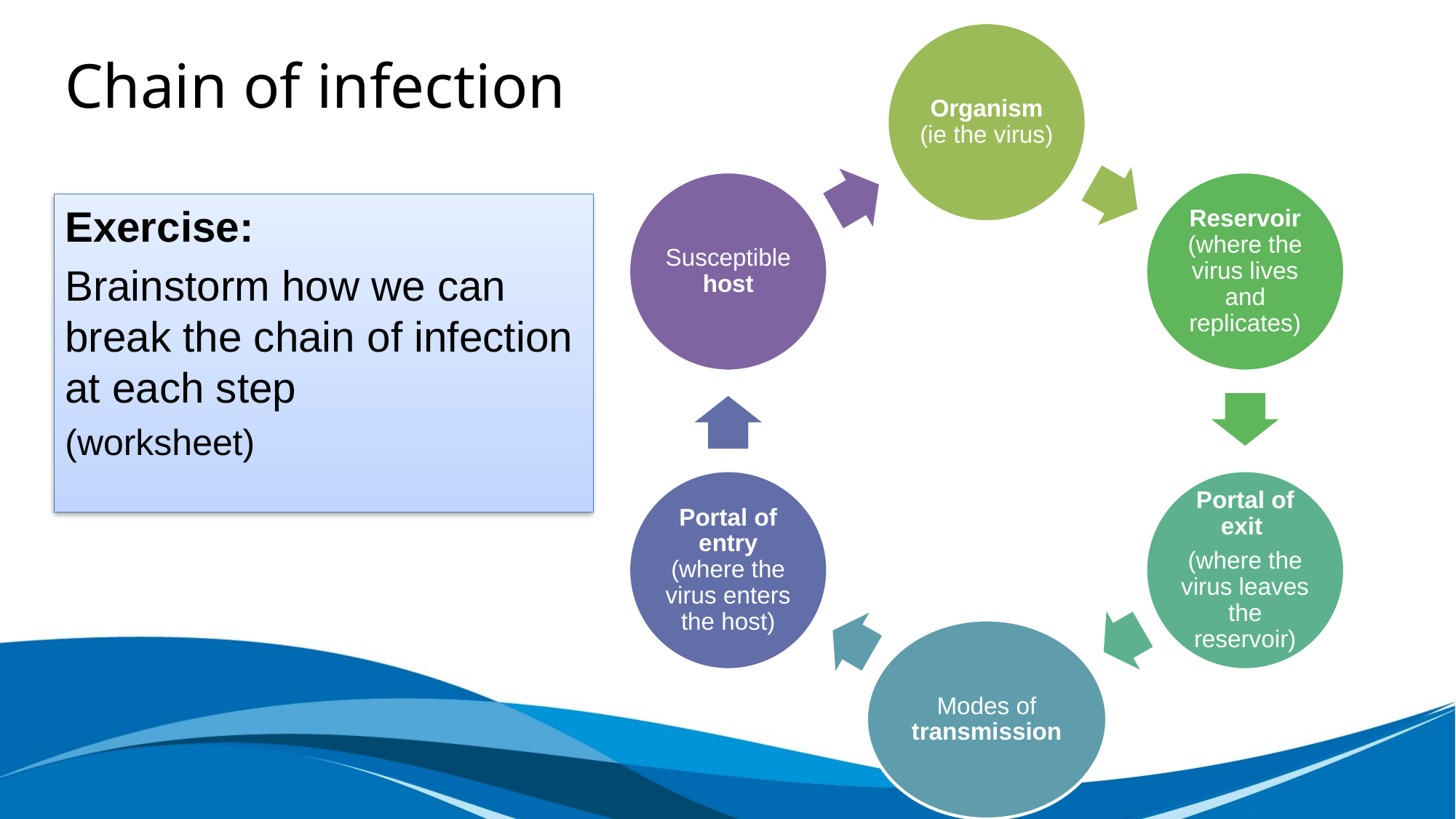

Chain of infection
Exercise:
Brainstorm how we can break the chain of infection at each step
(worksheet)

## Slide 6
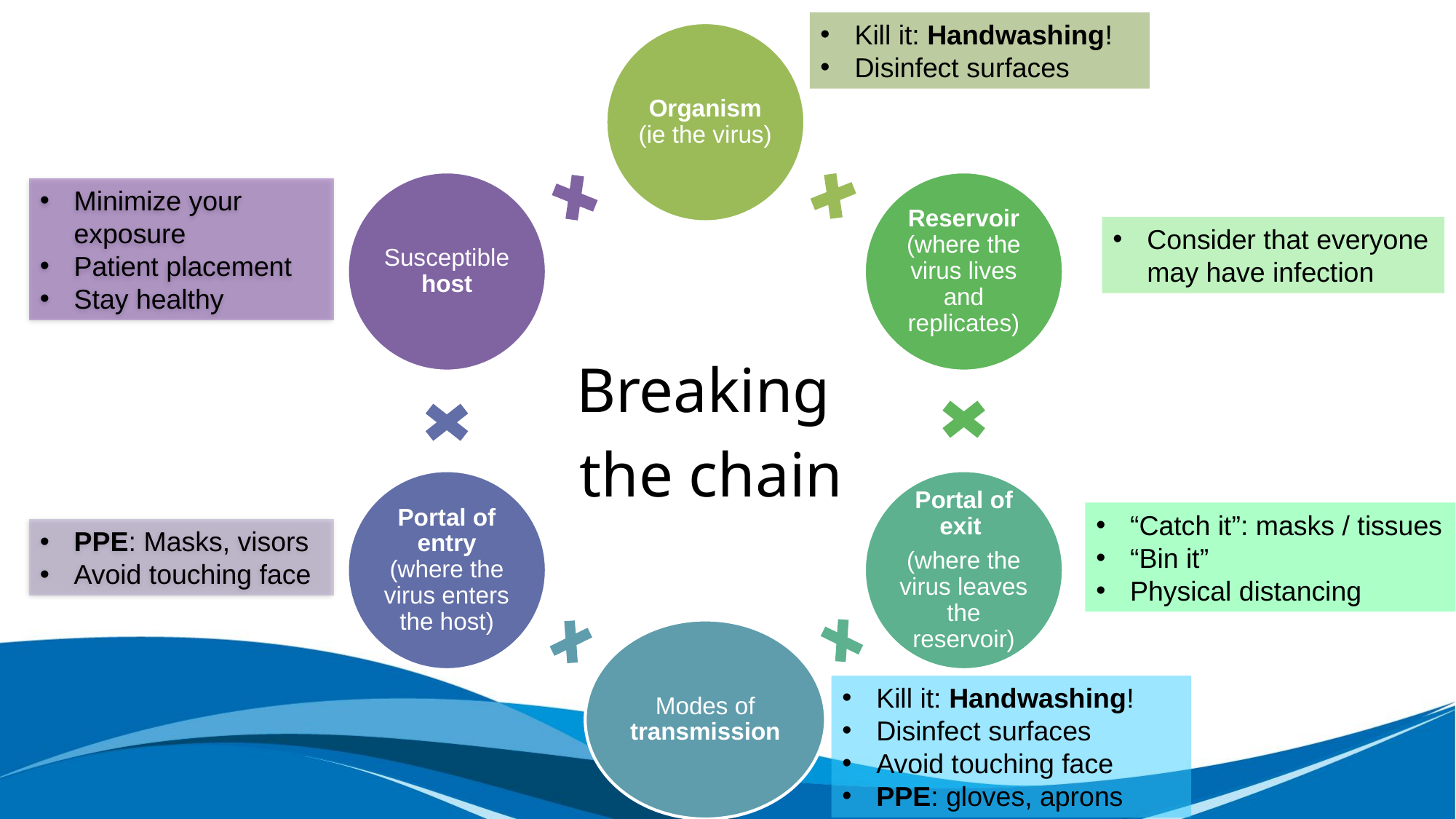

Kill it: Handwashing!
Disinfect surfaces
Minimize your exposure
Patient placement
Stay healthy
Consider that everyone may have infection
Breaking
 the chain
“Catch it”: masks / tissues
“Bin it”
Physical distancing
PPE: Masks, visors
Avoid touching face
Kill it: Handwashing!
Disinfect surfaces
Avoid touching face
PPE: gloves, aprons

## Slide 7
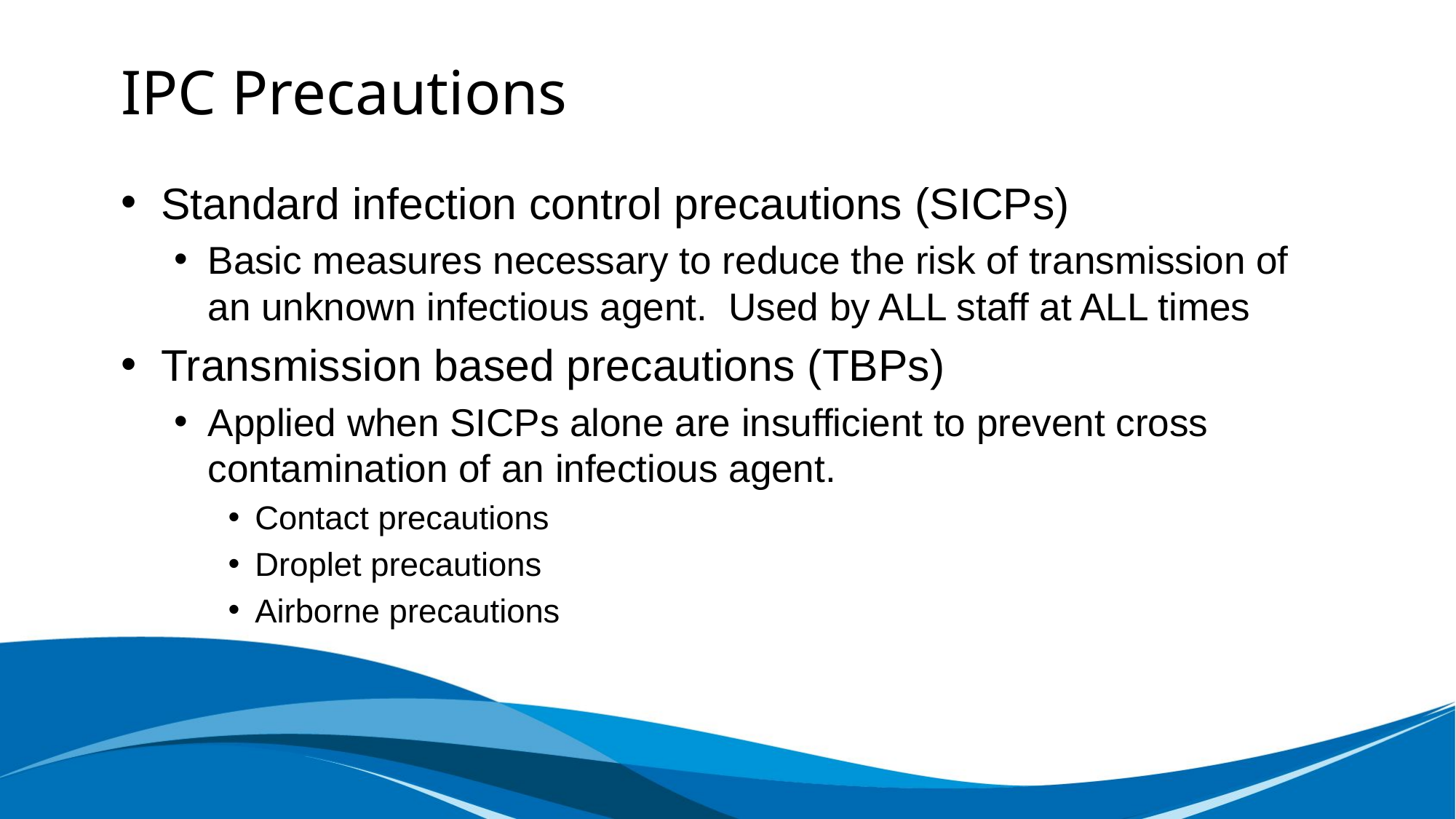

IPC Precautions
Standard infection control precautions (SICPs)
Basic measures necessary to reduce the risk of transmission of an unknown infectious agent. Used by ALL staff at ALL times
Transmission based precautions (TBPs)
Applied when SICPs alone are insufficient to prevent cross contamination of an infectious agent.
Contact precautions
Droplet precautions
Airborne precautions

## Slide 8
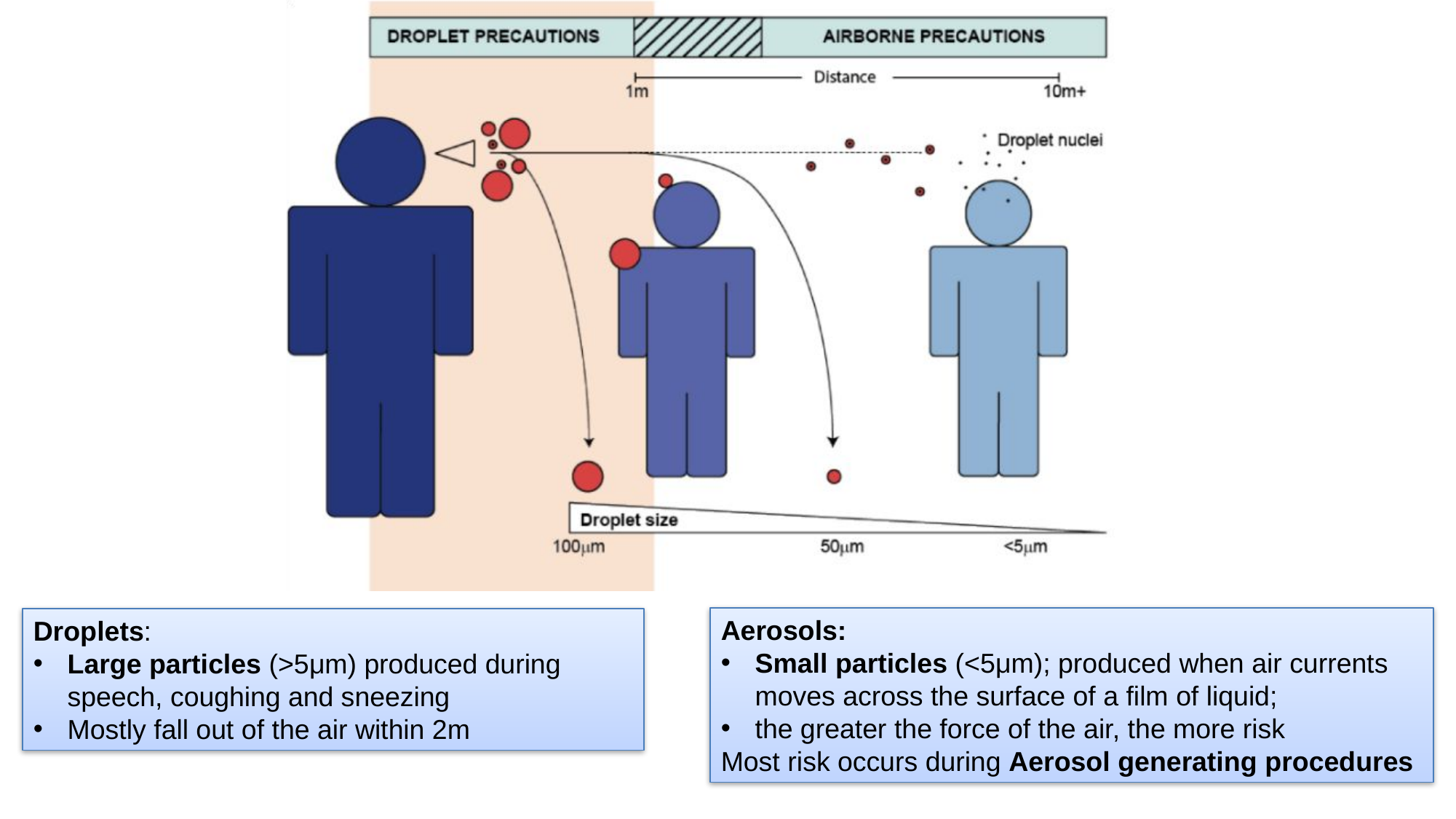

Aerosols:
Small particles (<5μm); produced when air currents moves across the surface of a film of liquid;
the greater the force of the air, the more risk
Most risk occurs during Aerosol generating procedures
Droplets:
Large particles (>5μm) produced during speech, coughing and sneezing
Mostly fall out of the air within 2m

## Slide 9
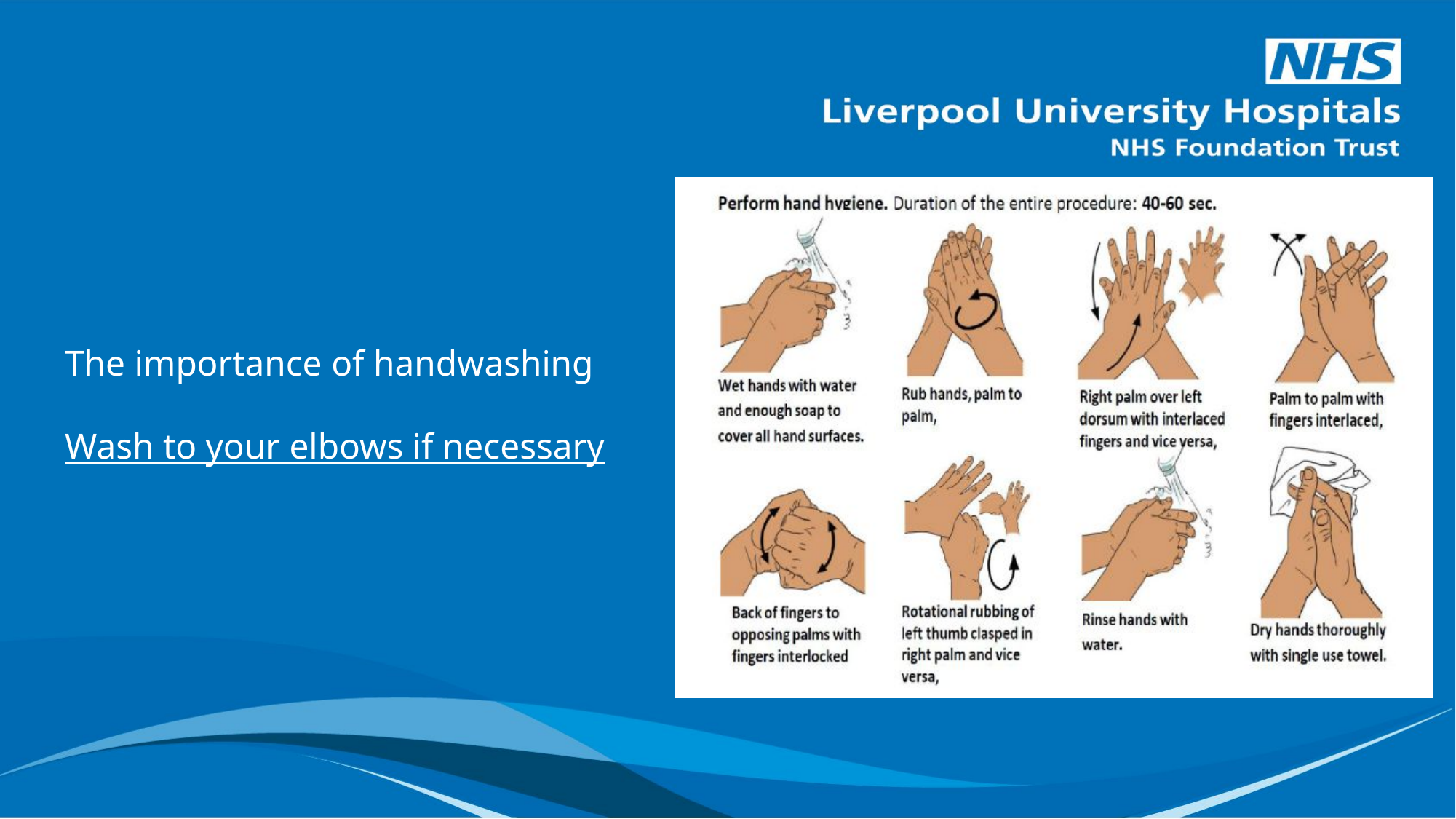

# The importance of handwashing Wash to your elbows if necessary

## Slide 10
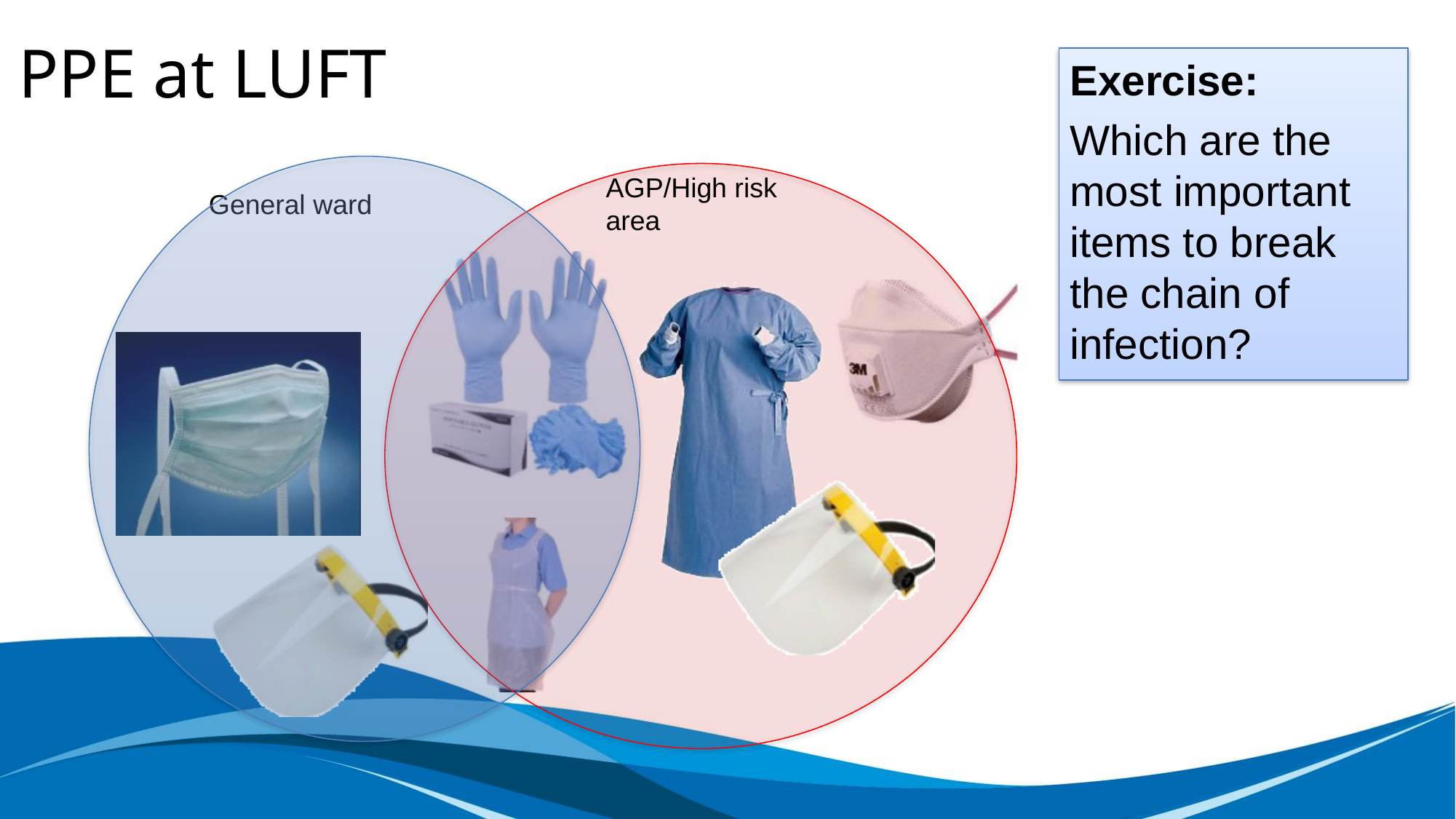

PPE at LUFT
Exercise:
Which are the most important items to break the chain of infection?
AGP/High risk area
General ward

## Slide 11
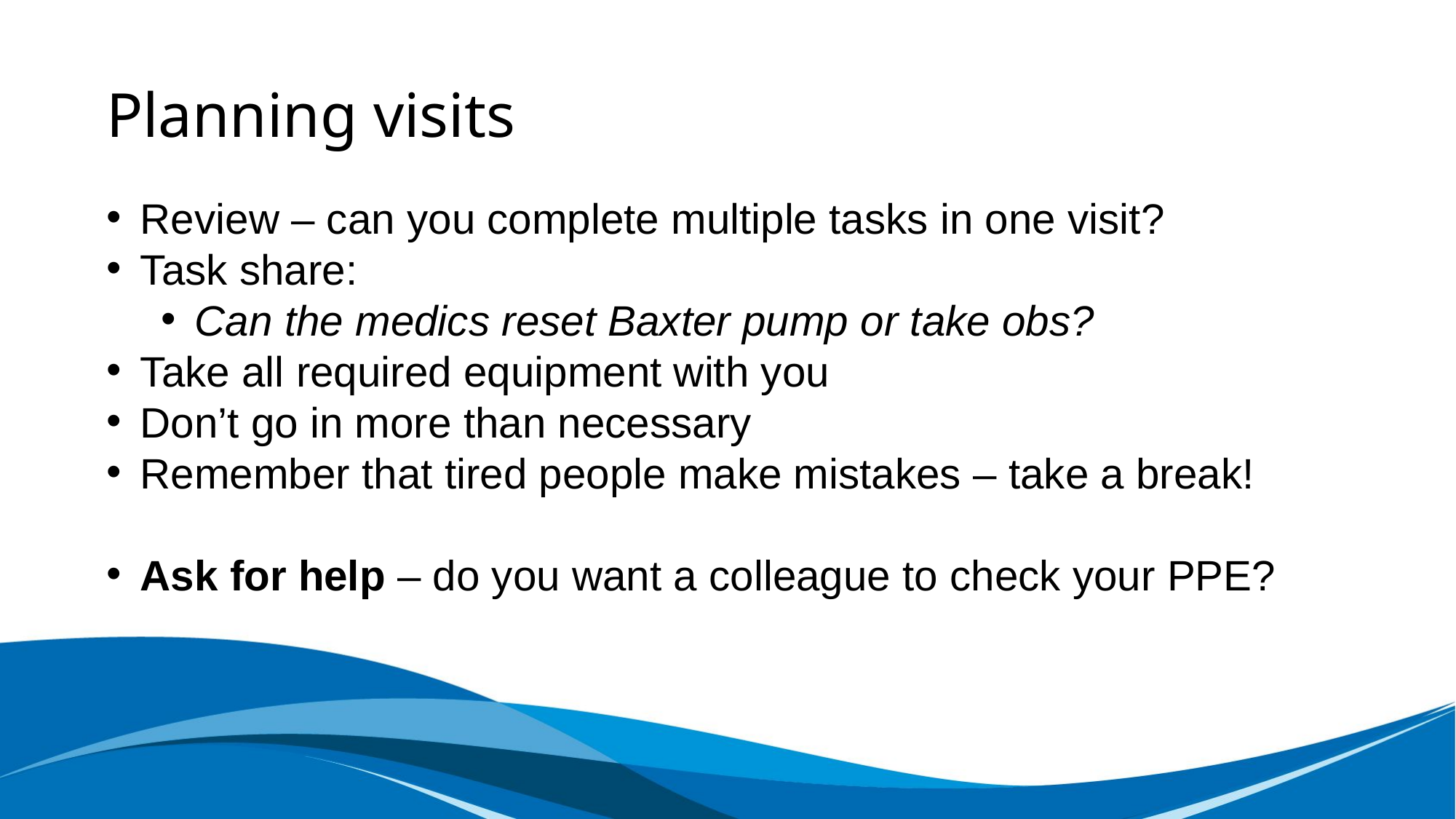

Planning visits
Review – can you complete multiple tasks in one visit?
Task share:
Can the medics reset Baxter pump or take obs?
Take all required equipment with you
Don’t go in more than necessary
Remember that tired people make mistakes – take a break!
Ask for help – do you want a colleague to check your PPE?

## Slide 12
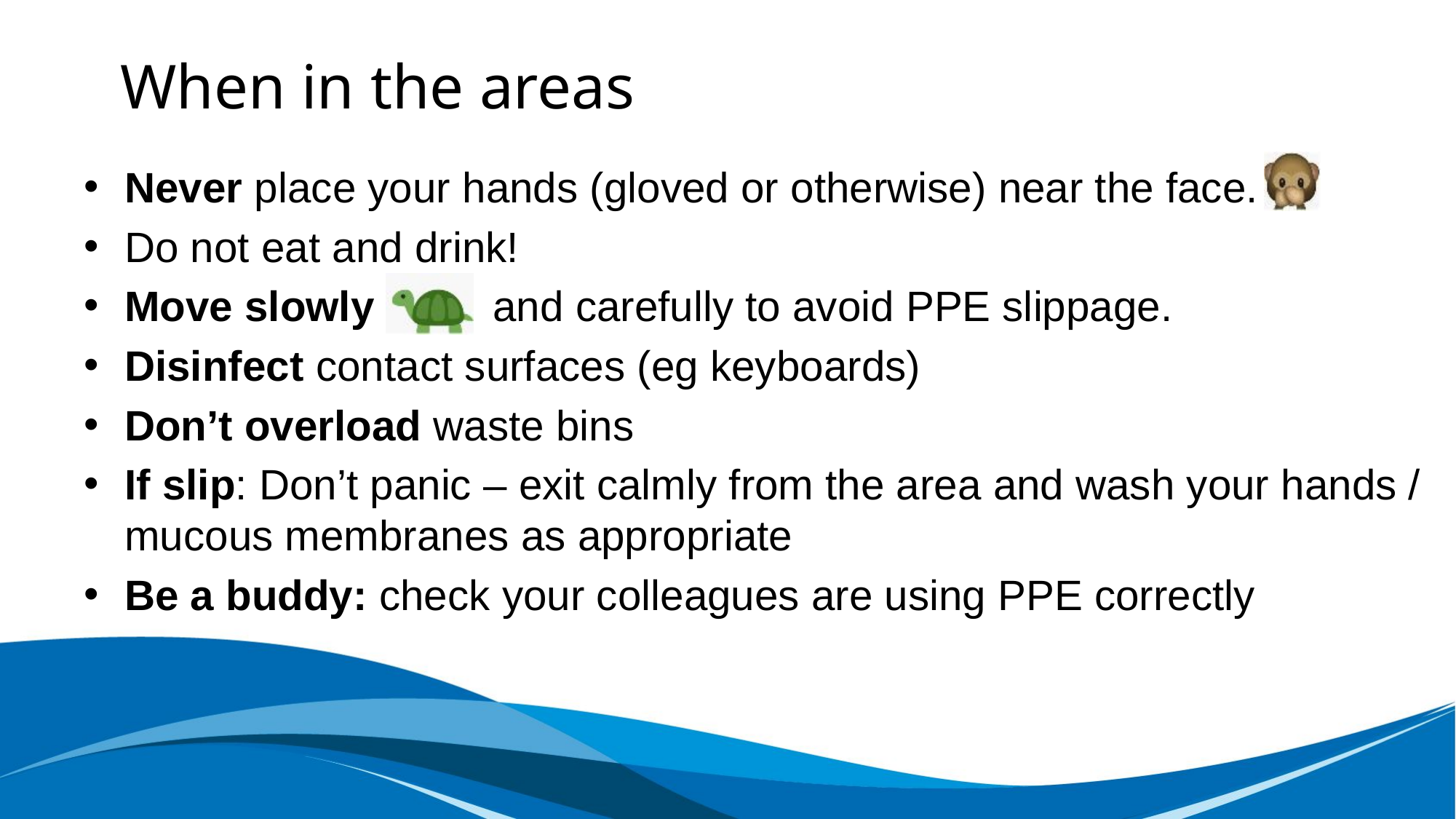

When in the areas
Never place your hands (gloved or otherwise) near the face.
Do not eat and drink!
Move slowly and carefully to avoid PPE slippage.
Disinfect contact surfaces (eg keyboards)
Don’t overload waste bins
If slip: Don’t panic – exit calmly from the area and wash your hands / mucous membranes as appropriate
Be a buddy: check your colleagues are using PPE correctly

## Slide 13
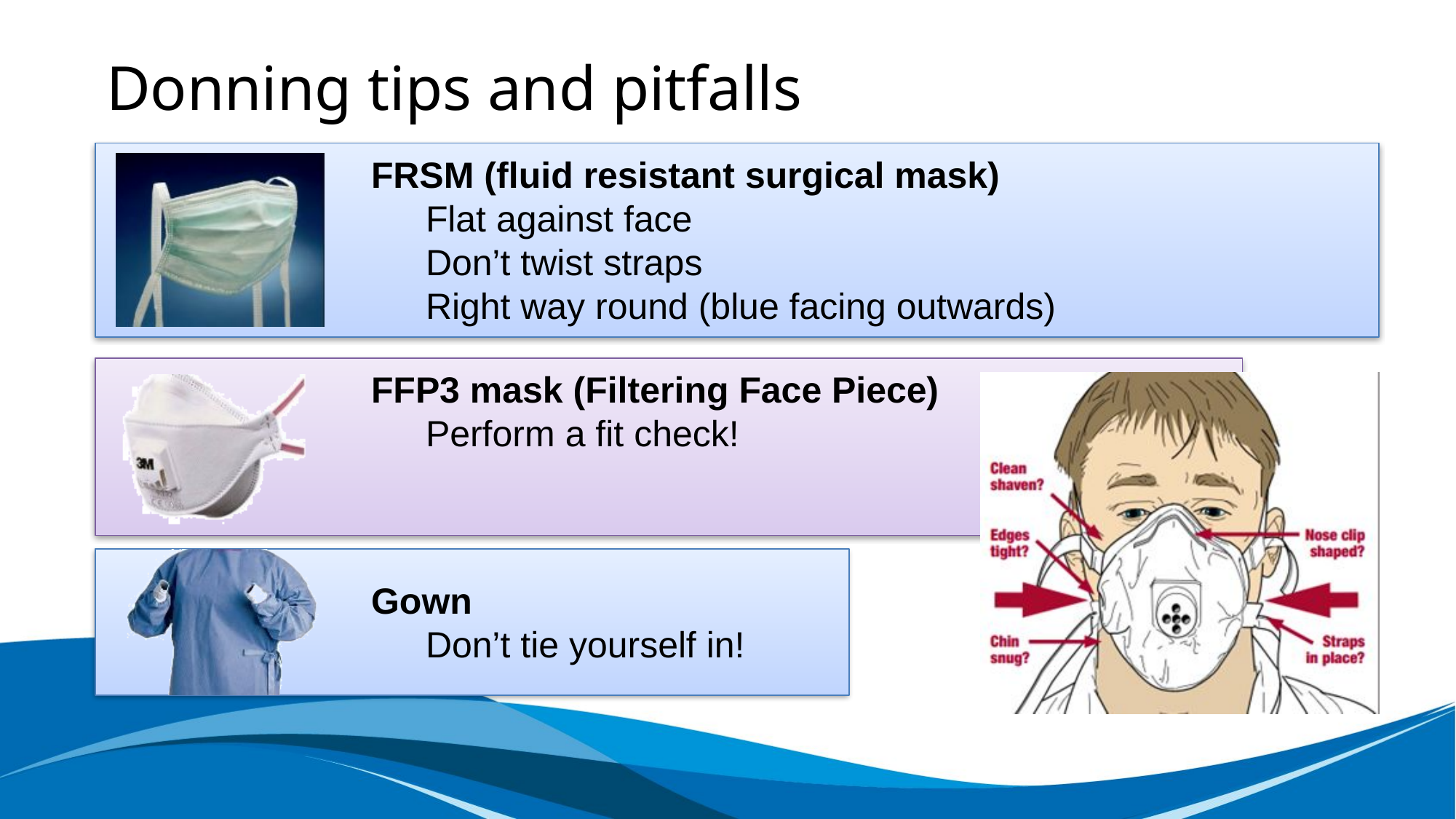

Donning tips and pitfalls
FRSM (fluid resistant surgical mask)
Flat against face
Don’t twist straps
Right way round (blue facing outwards)
FFP3 mask (Filtering Face Piece)
Perform a fit check!
Gown
Don’t tie yourself in!

## Slide 14
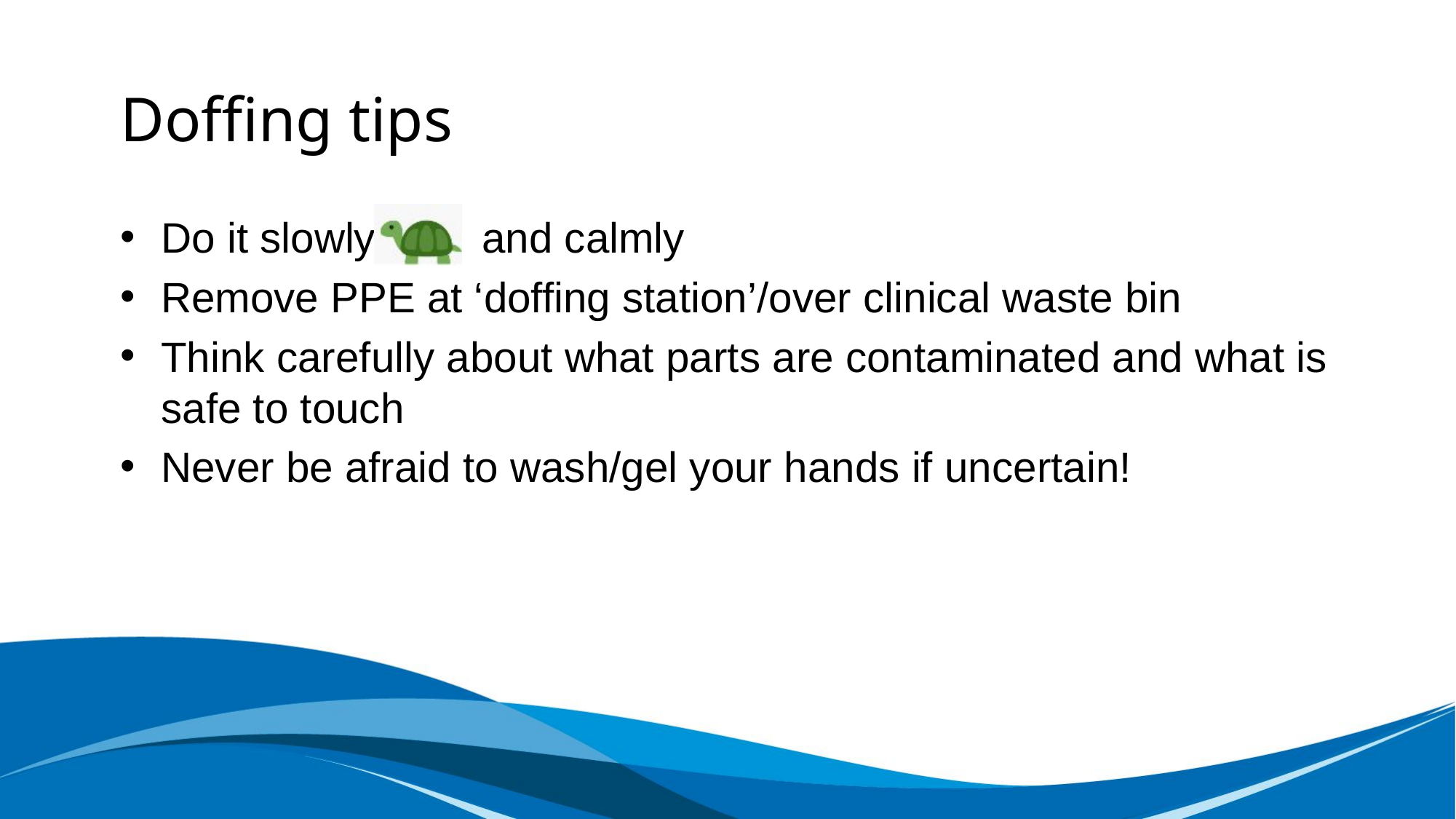

Doffing tips
Do it slowly and calmly
Remove PPE at ‘doffing station’/over clinical waste bin
Think carefully about what parts are contaminated and what is safe to touch
Never be afraid to wash/gel your hands if uncertain!

## Slide 15
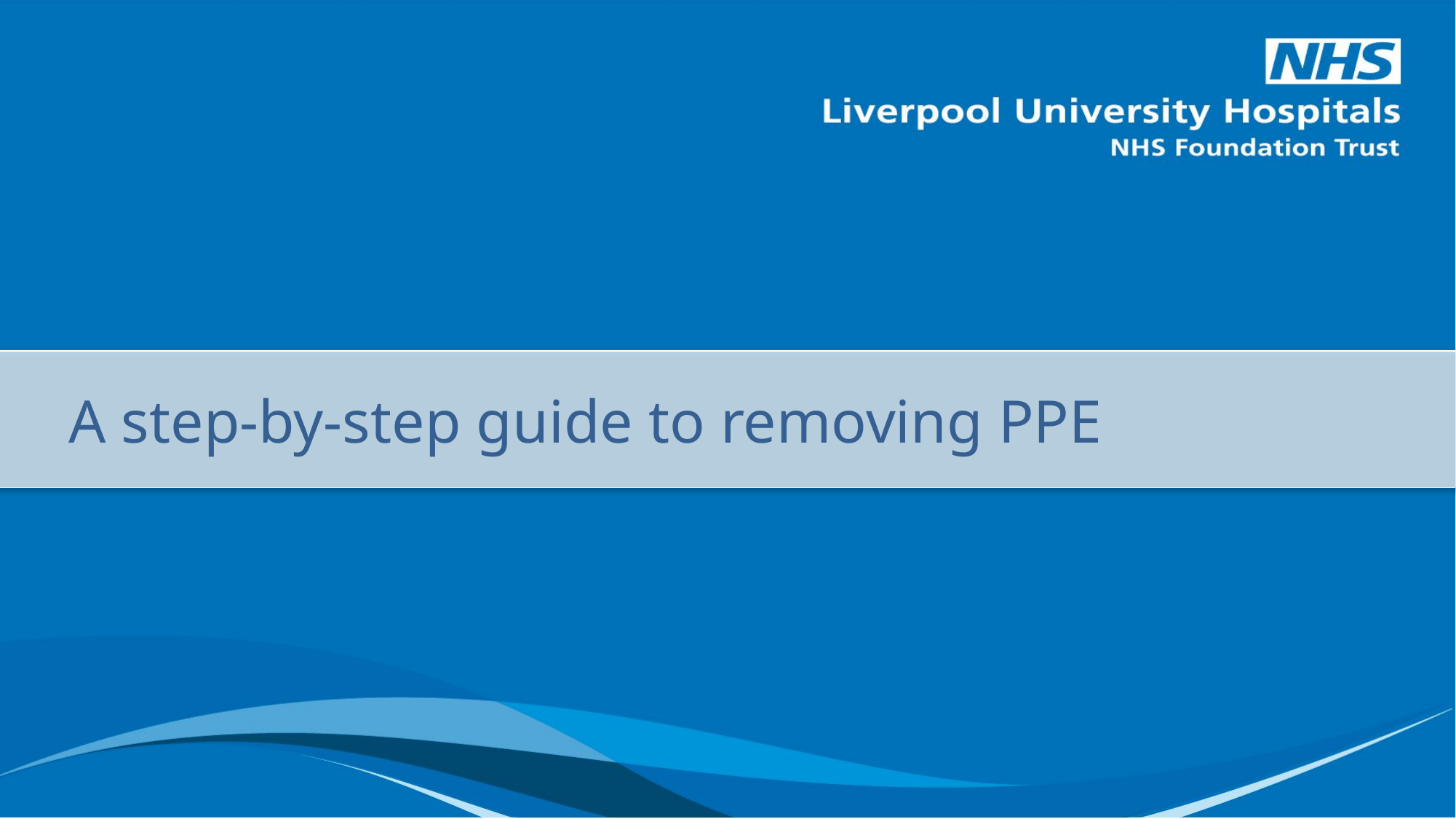

# A step-by-step guide to removing PPE

## Slide 16
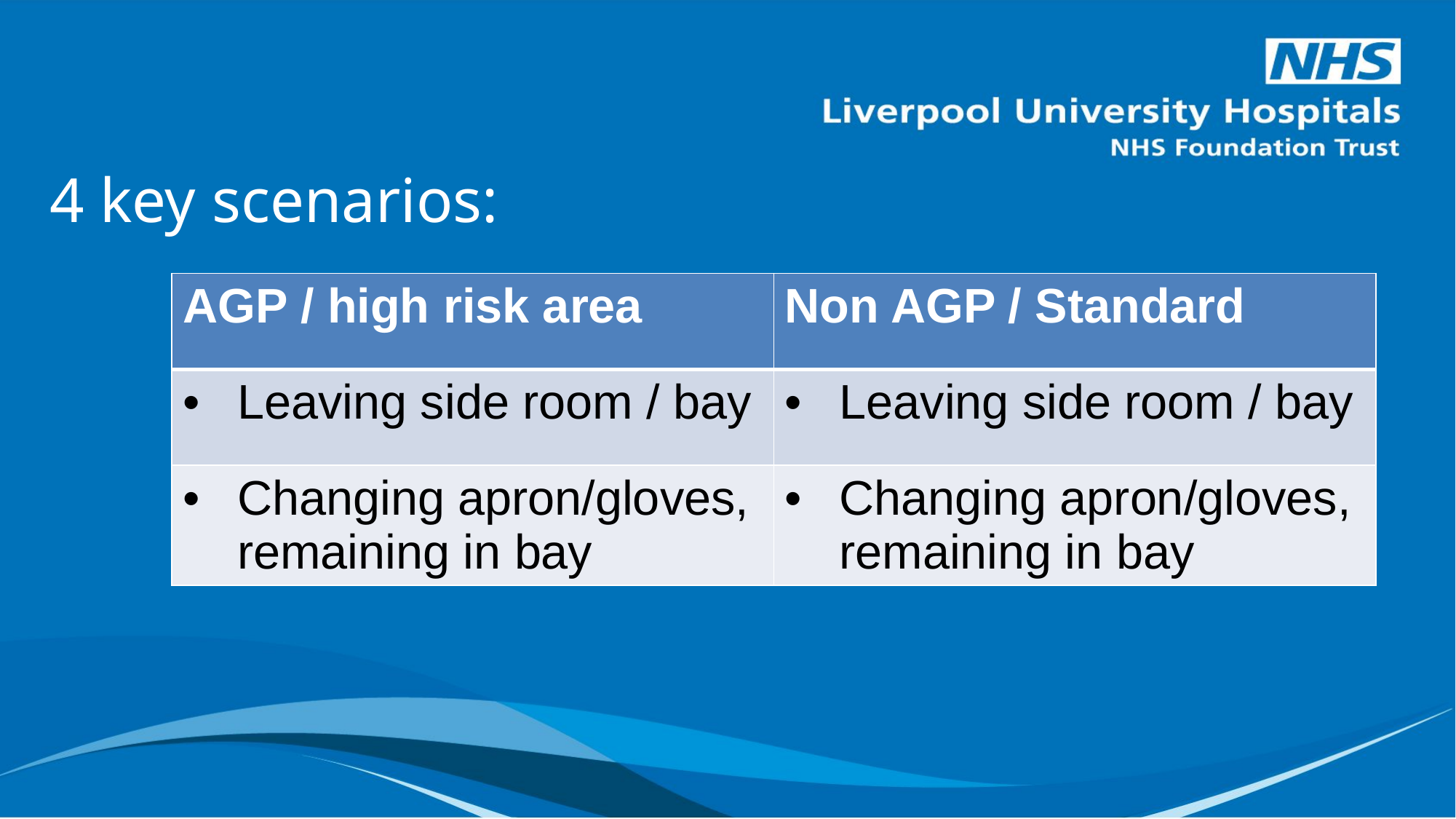

# 4 key scenarios:
| AGP / high risk area | Non AGP / Standard |
| --- | --- |
| Leaving side room / bay | Leaving side room / bay |
| Changing apron/gloves, remaining in bay | Changing apron/gloves, remaining in bay |

## Slide 17
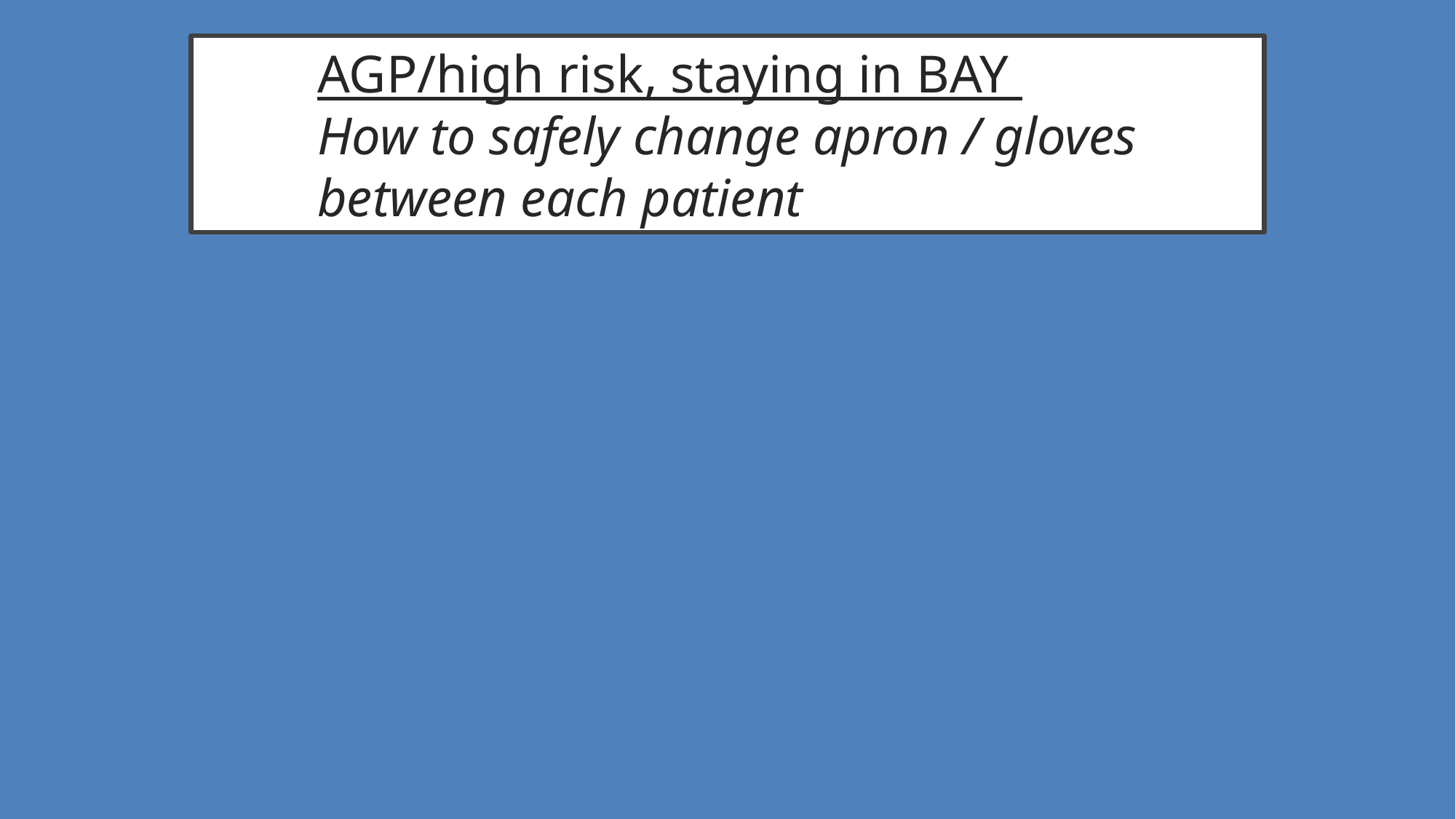

# AGP/high risk, staying in BAY How to safely change apron / gloves between each patient

## Slide 18
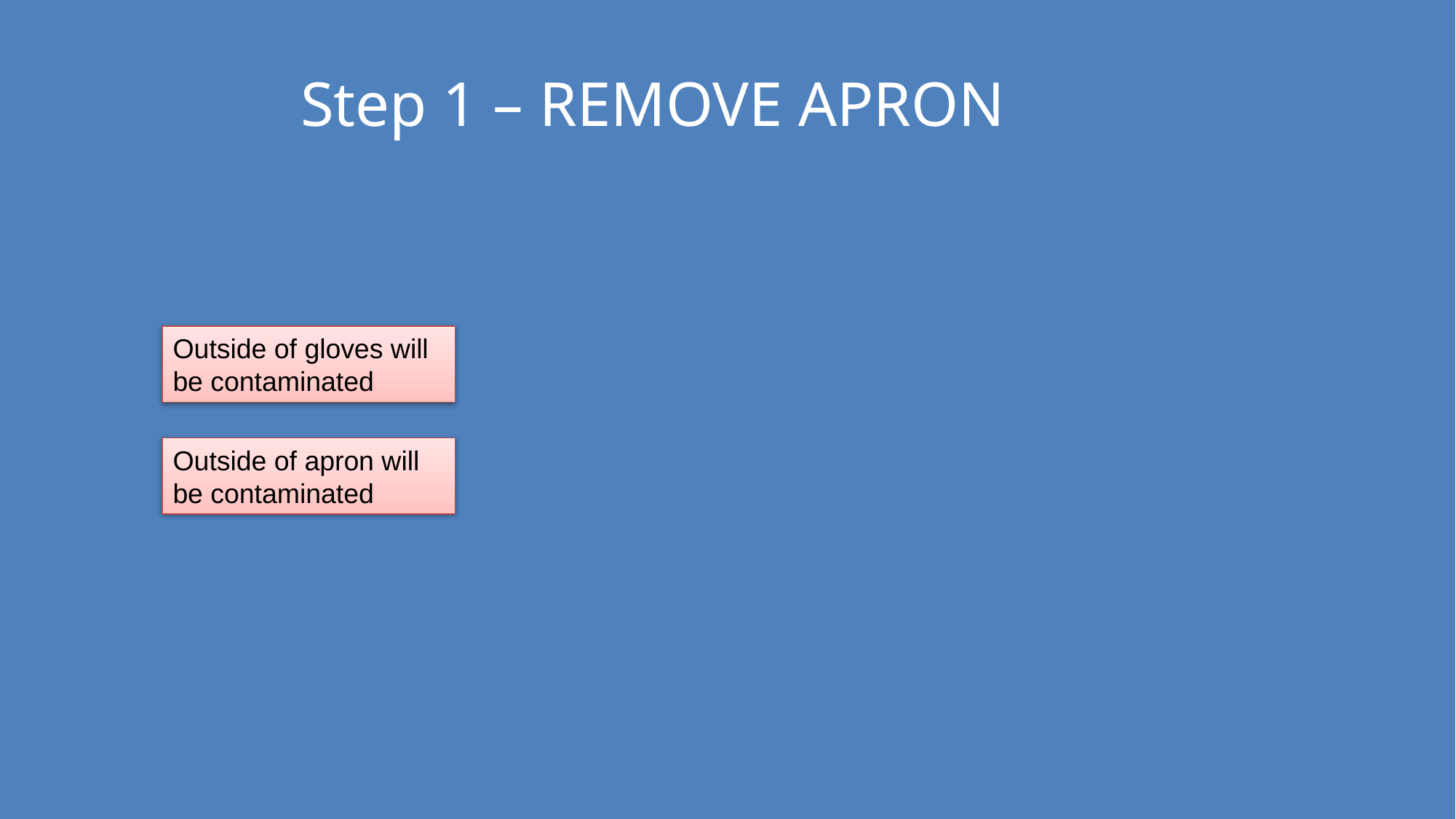

# Step 1 – REMOVE APRON
Outside of gloves will be contaminated
Outside of apron will be contaminated

## Slide 19
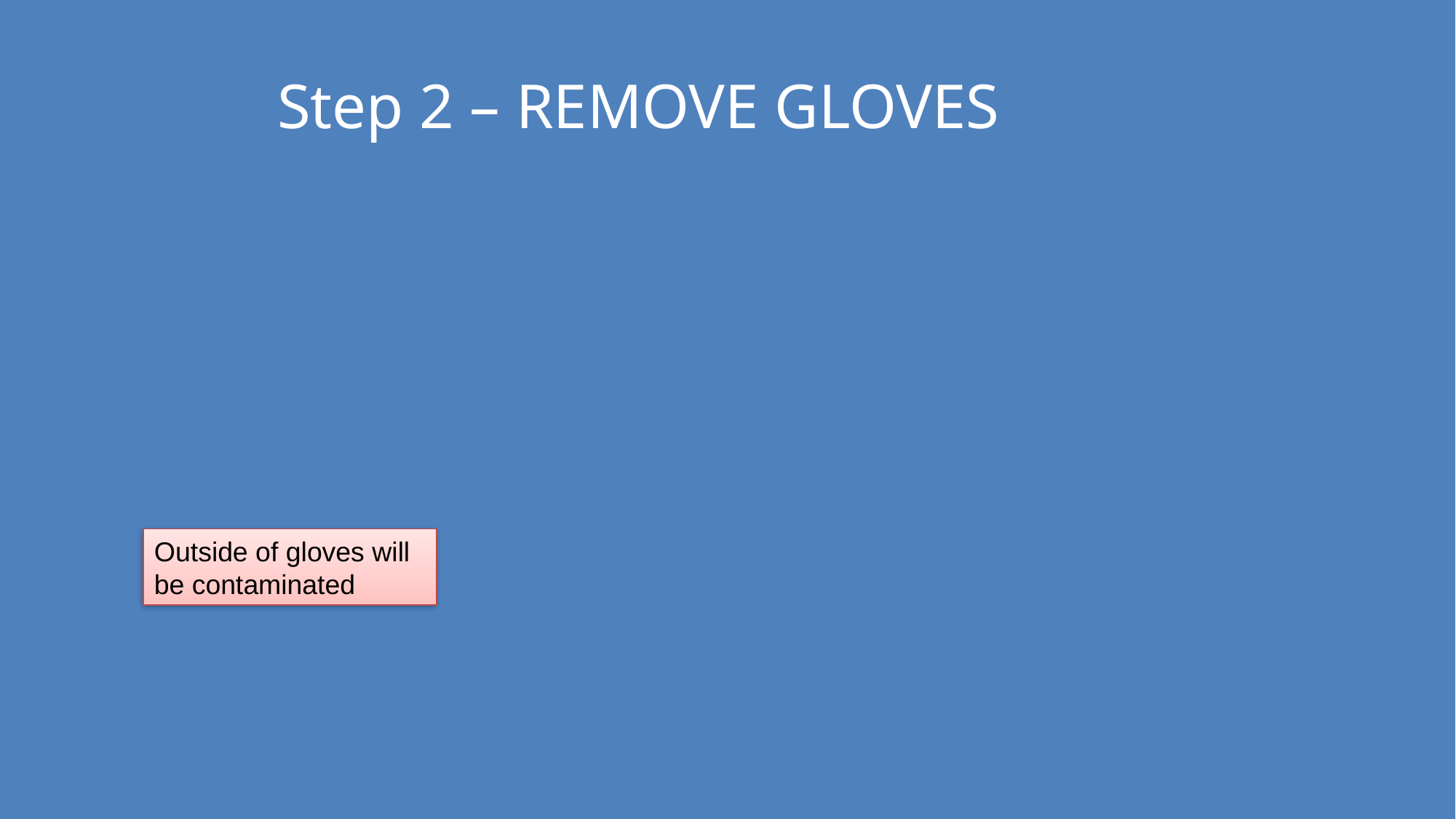

# Step 2 – REMOVE GLOVES
Outside of gloves will be contaminated

## Slide 20
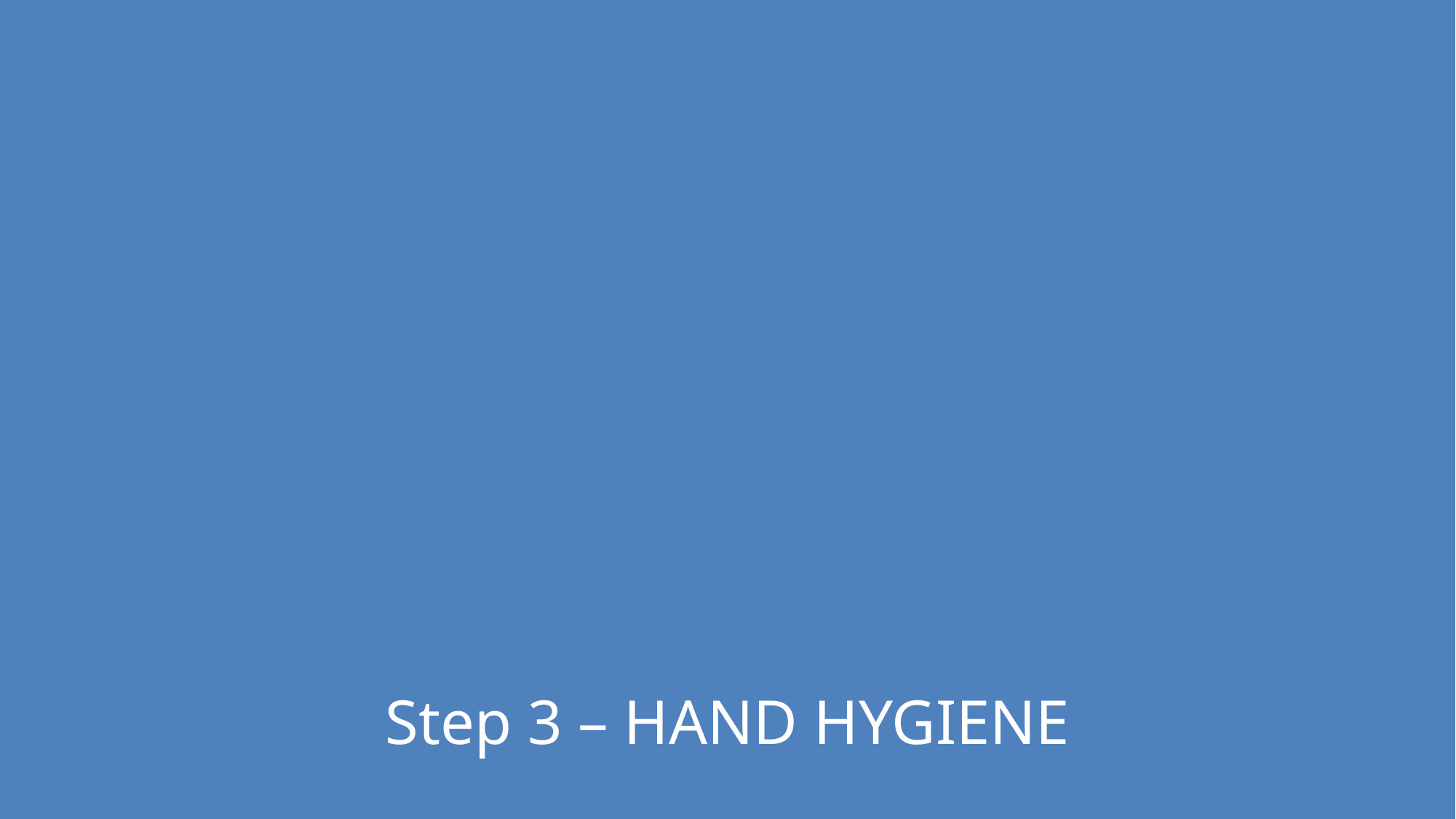

# Step 3 – HAND HYGIENE

## Slide 21
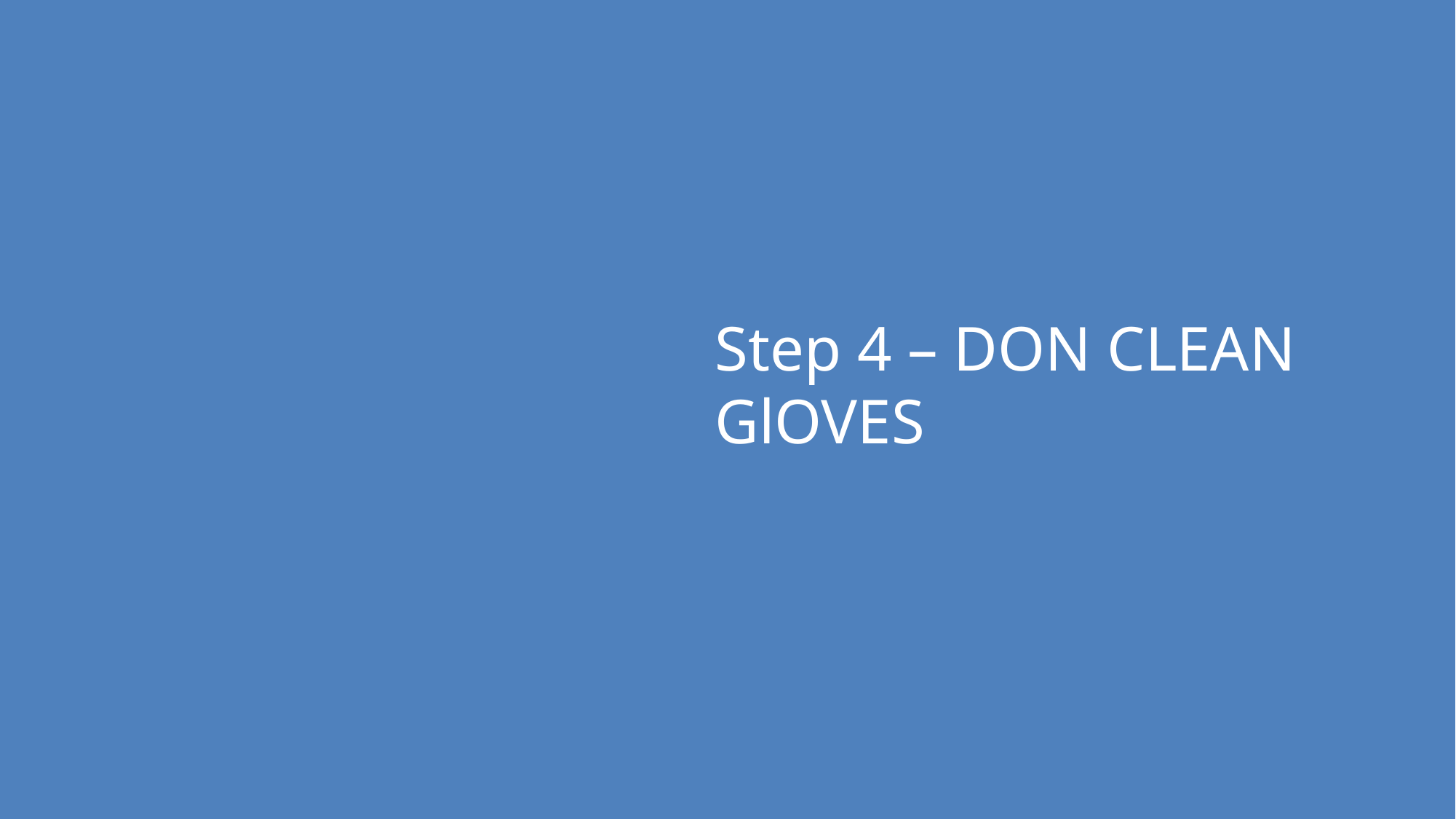

# Step 4 – DON CLEAN GlOVES

## Slide 22
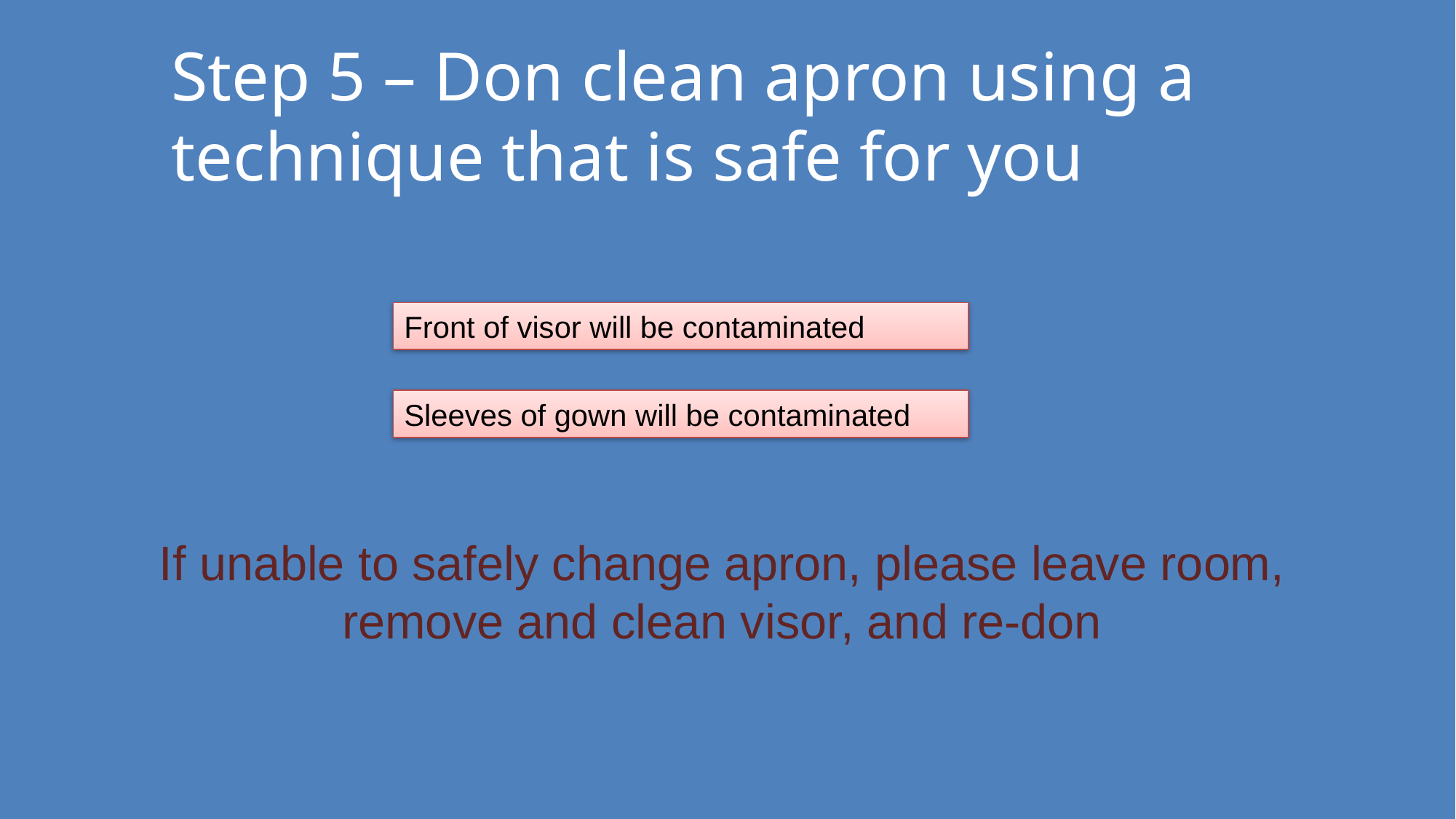

# Step 5 – Don clean apron using a technique that is safe for you
Front of visor will be contaminated
Sleeves of gown will be contaminated
If unable to safely change apron, please leave room, remove and clean visor, and re-don

## Slide 23
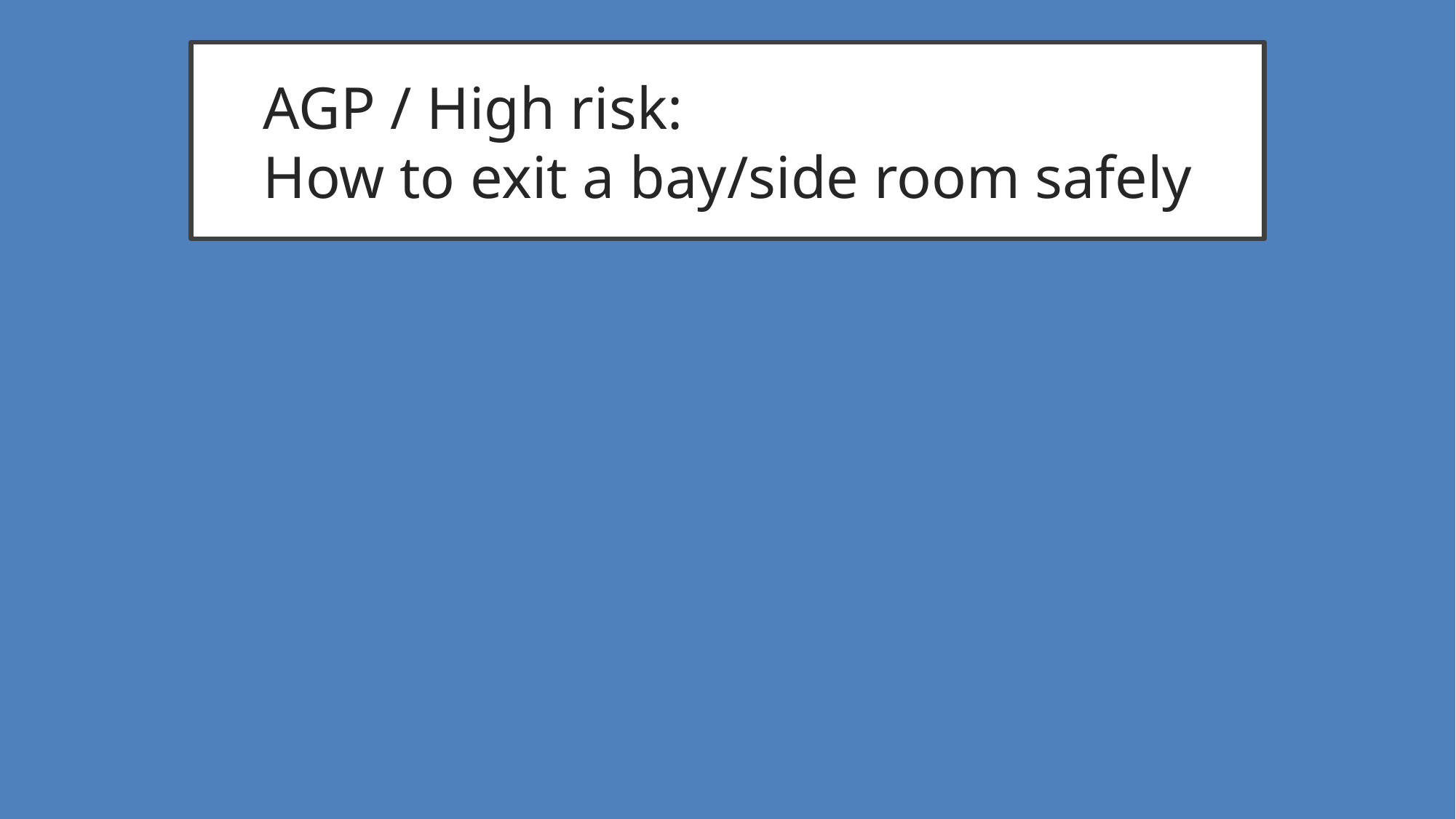

# AGP / High risk: How to exit a bay/side room safely

## Slide 24
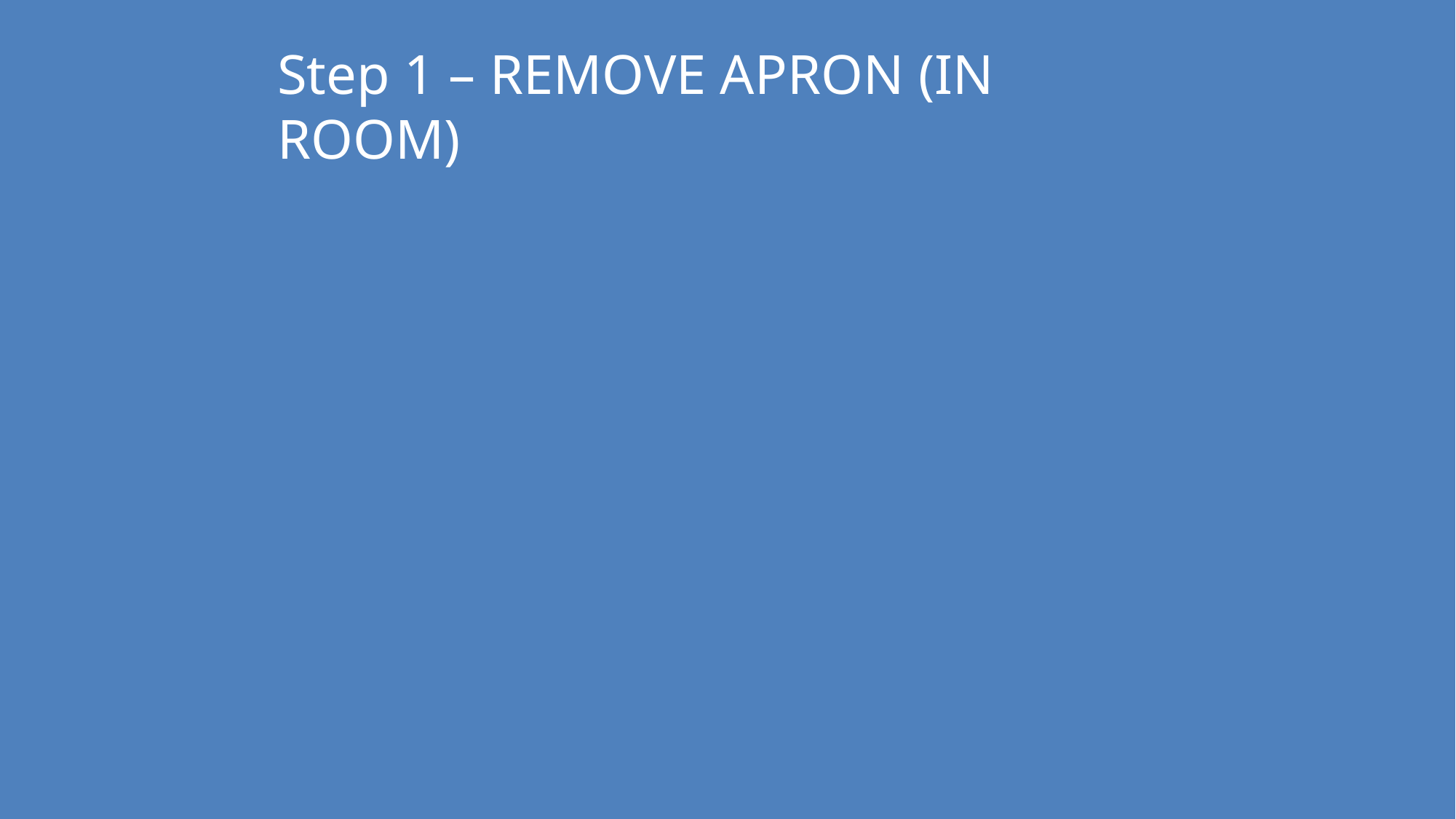

# Step 1 – REMOVE APRON (IN ROOM)

## Slide 25
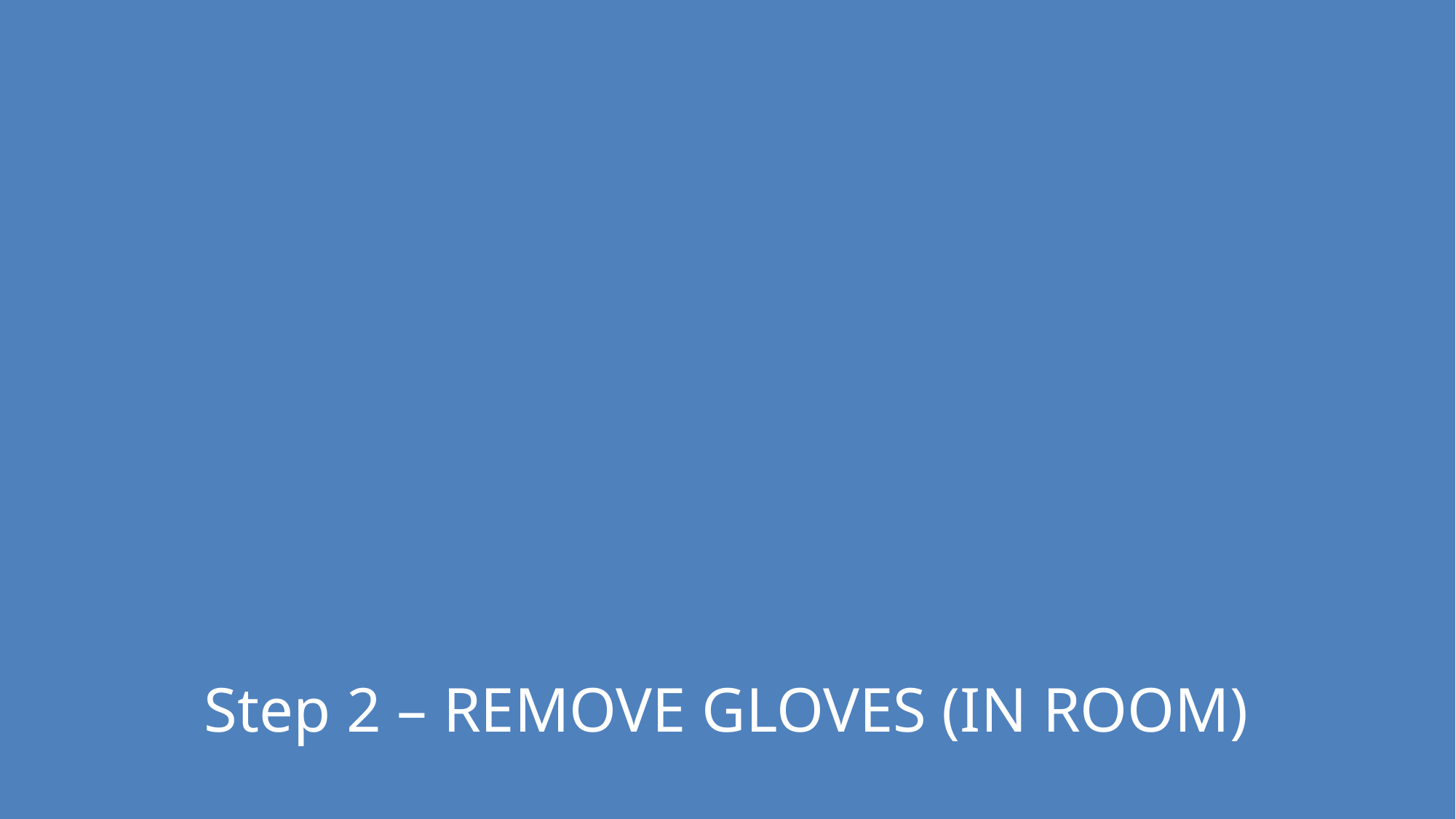

# Step 2 – REMOVE GLOVES (IN ROOM)

## Slide 26
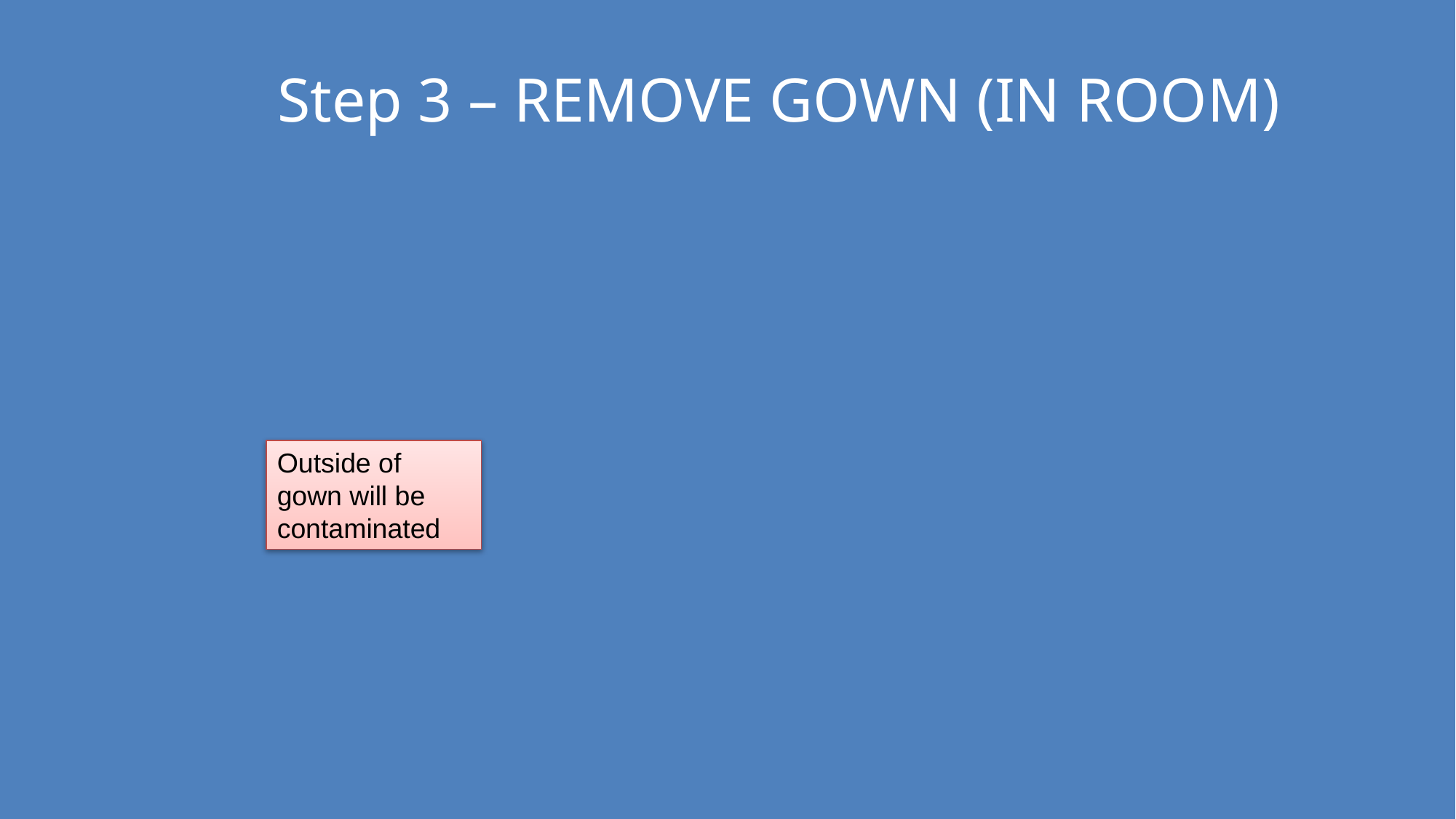

# Step 3 – REMOVE GOWN (IN ROOM)
Outside of gown will be contaminated

## Slide 27
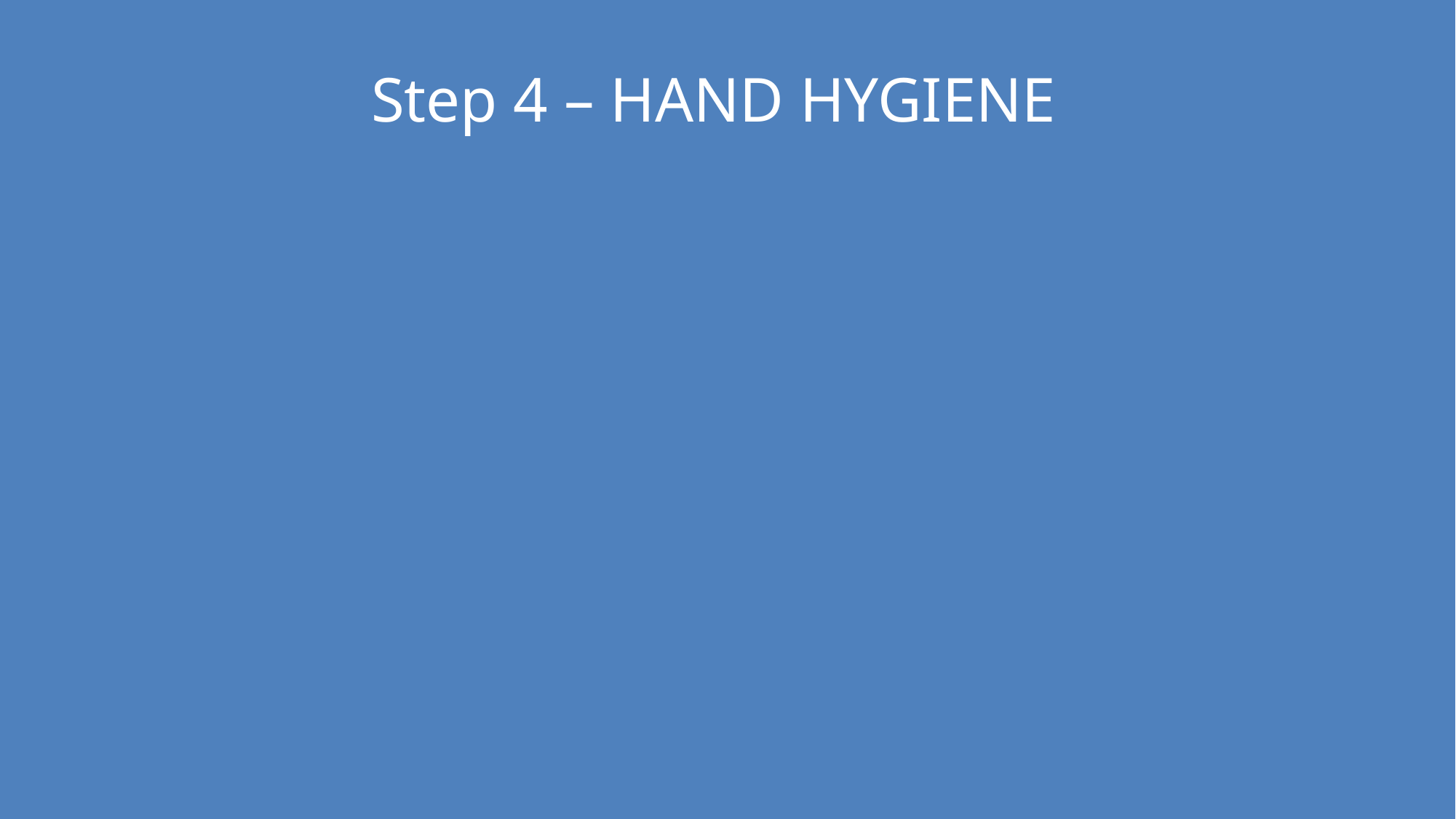

# Step 4 – HAND HYGIENE

## Slide 28
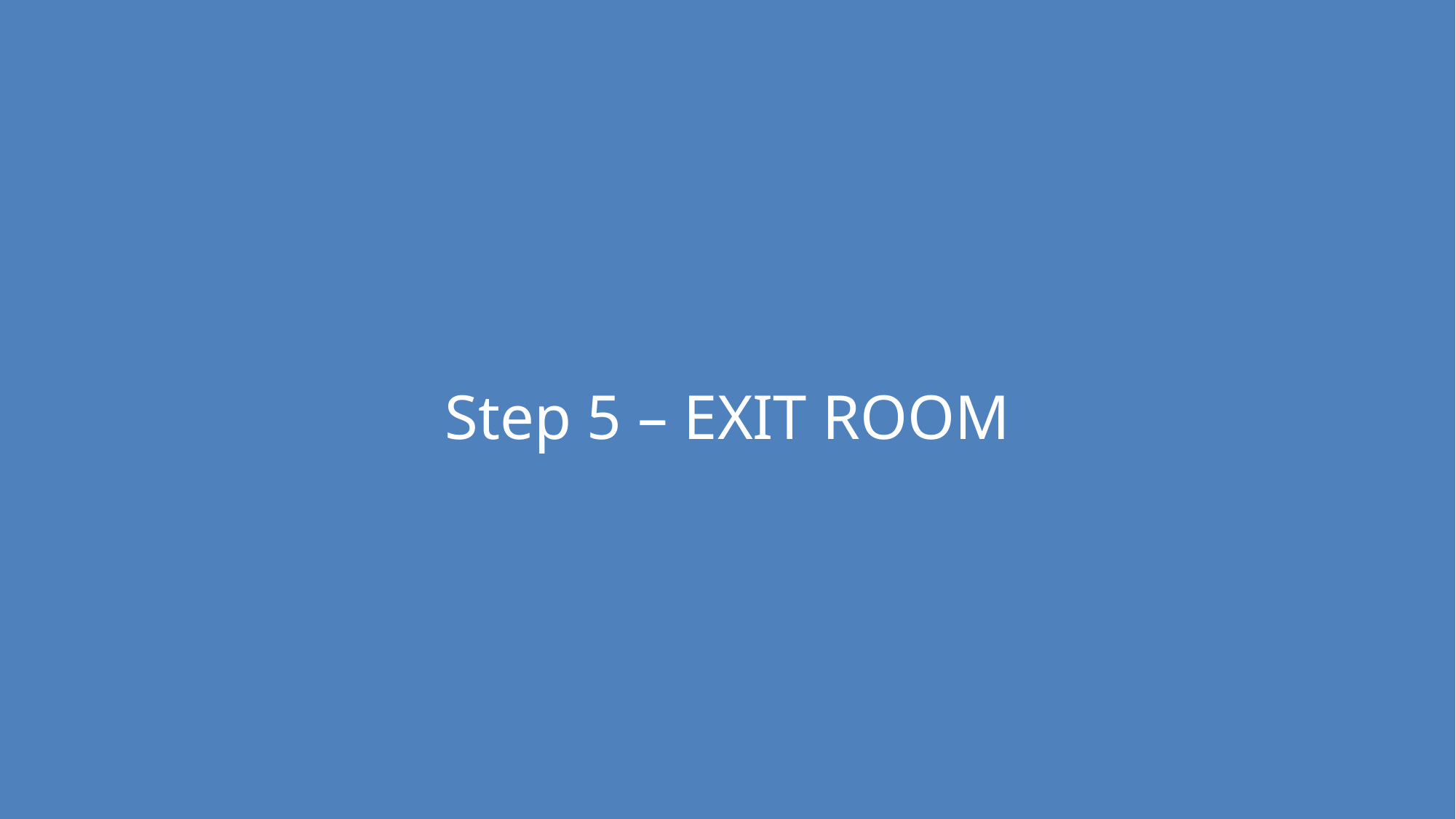

# Step 5 – EXIT ROOM

## Slide 29
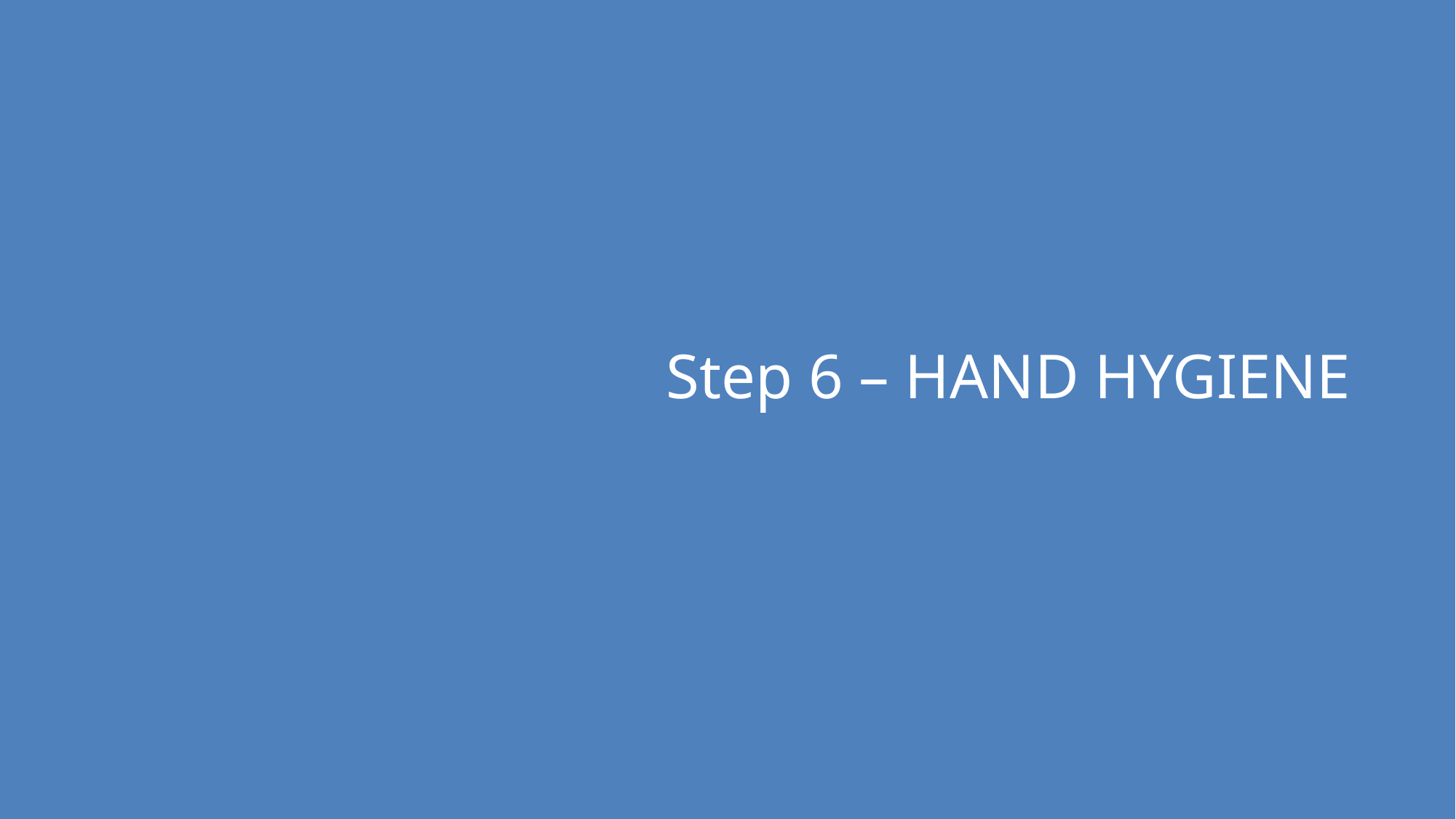

# Step 6 – HAND HYGIENE

## Slide 30
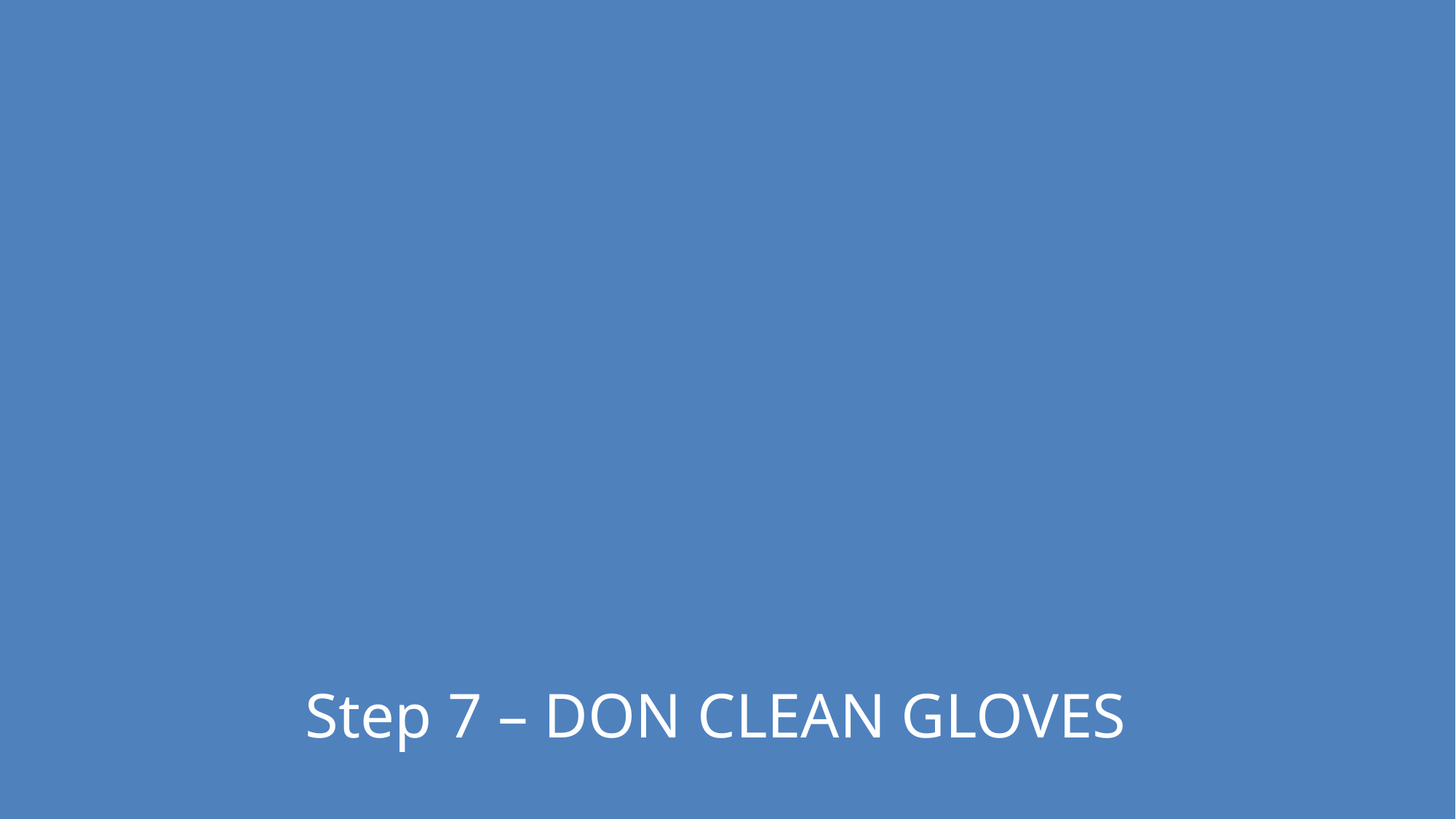

# Step 7 – DON CLEAN GLOVES

## Slide 31
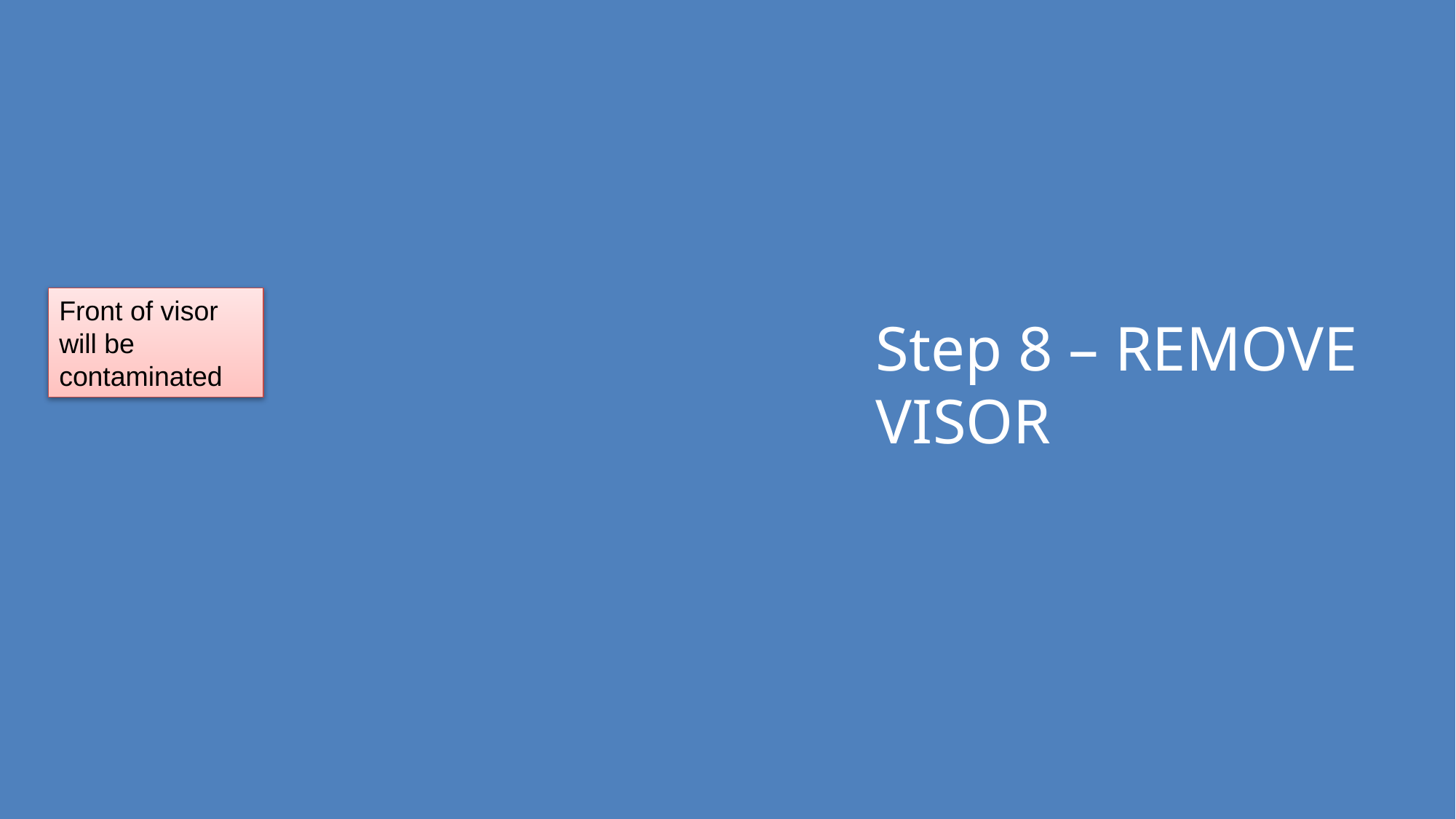

# Step 8 – REMOVE VISOR
Front of visor will be contaminated

## Slide 32
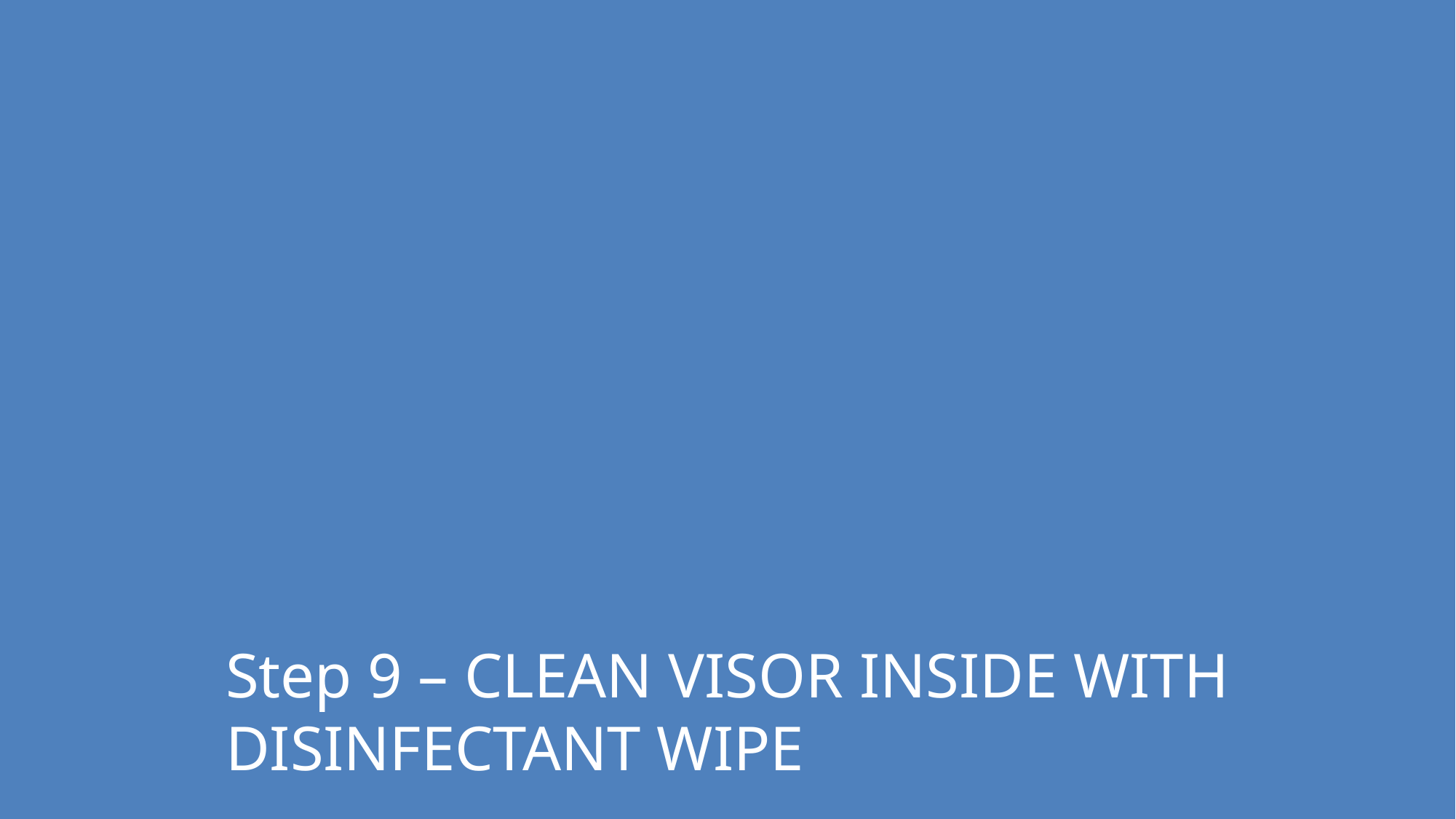

# Step 9 – CLEAN VISOR INSIDE WITH DISINFECTANT WIPE

## Slide 33
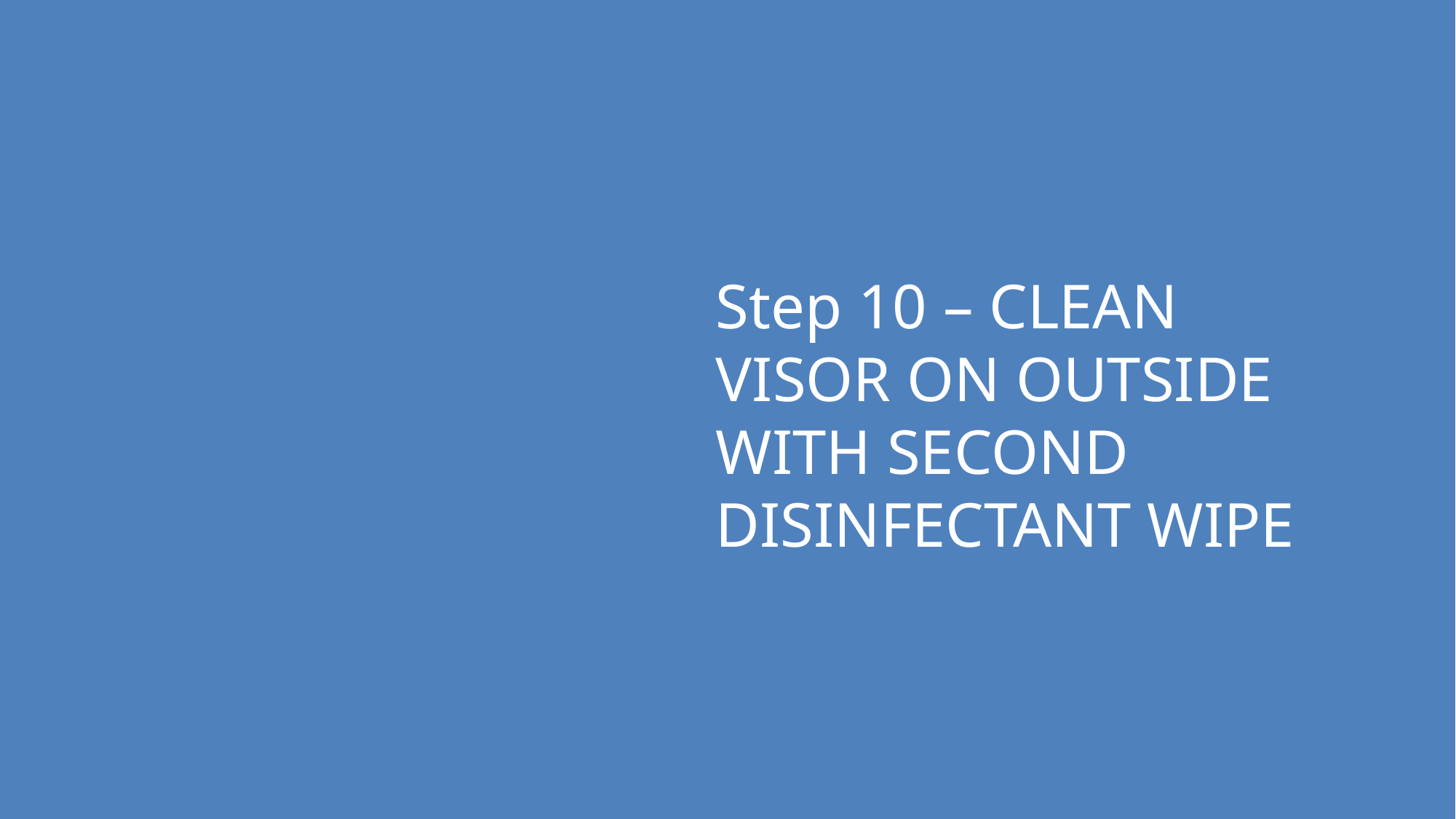

# Step 10 – CLEAN VISOR ON OUTSIDE WITH SECOND DISINFECTANT WIPE

## Slide 34
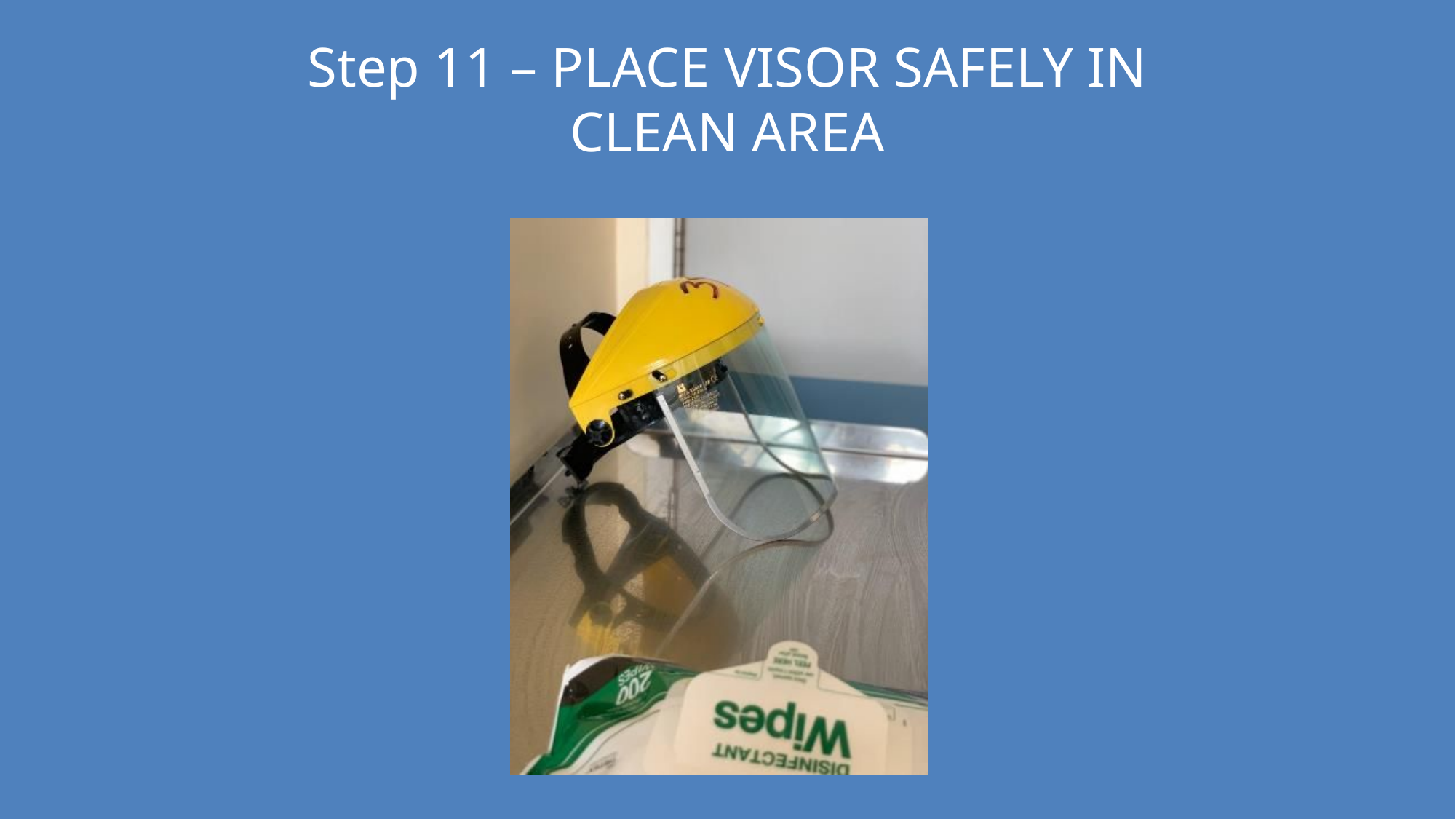

# Step 11 – PLACE VISOR SAFELY IN CLEAN AREA

## Slide 35
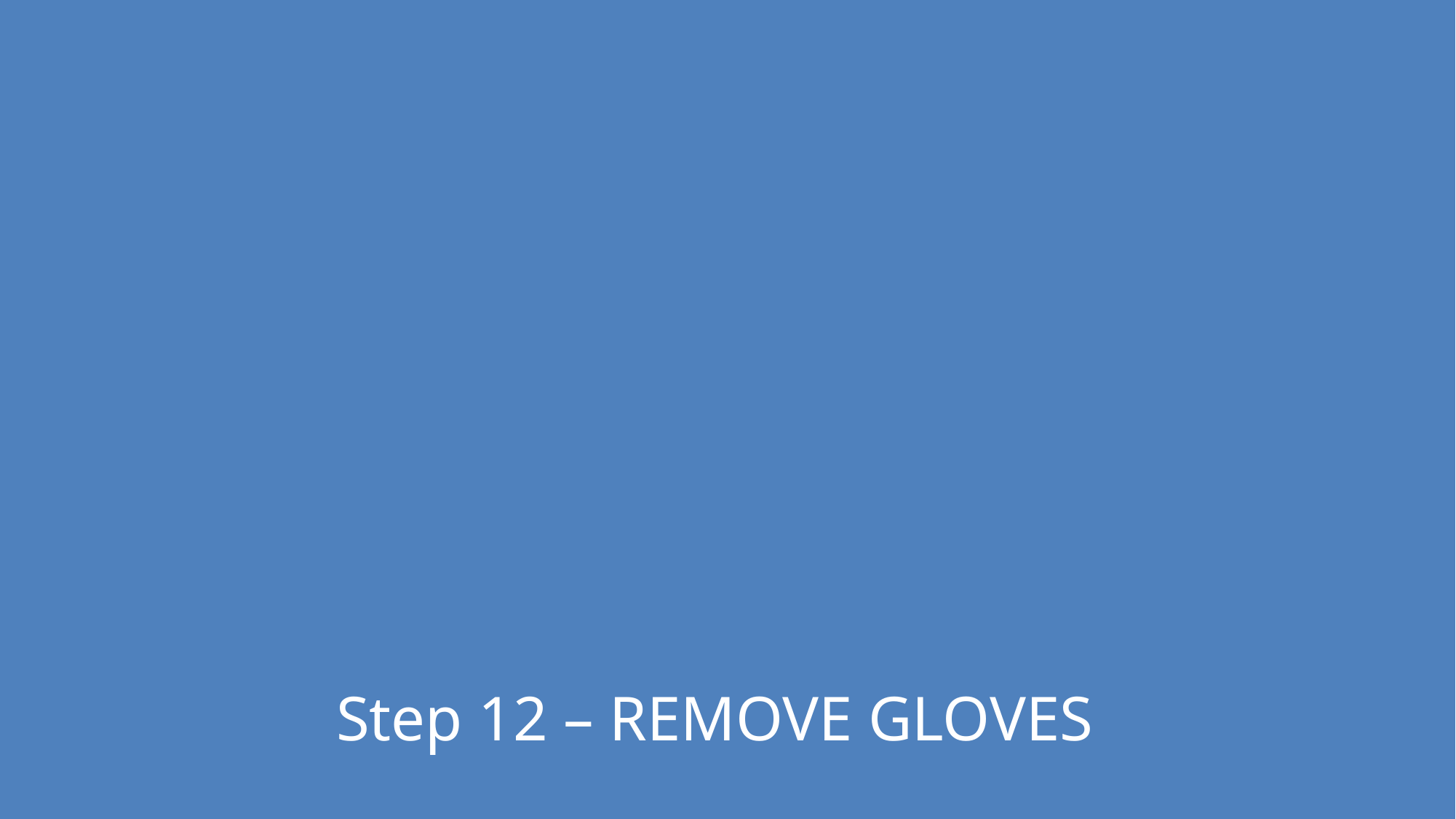

# Step 12 – REMOVE GLOVES

## Slide 36
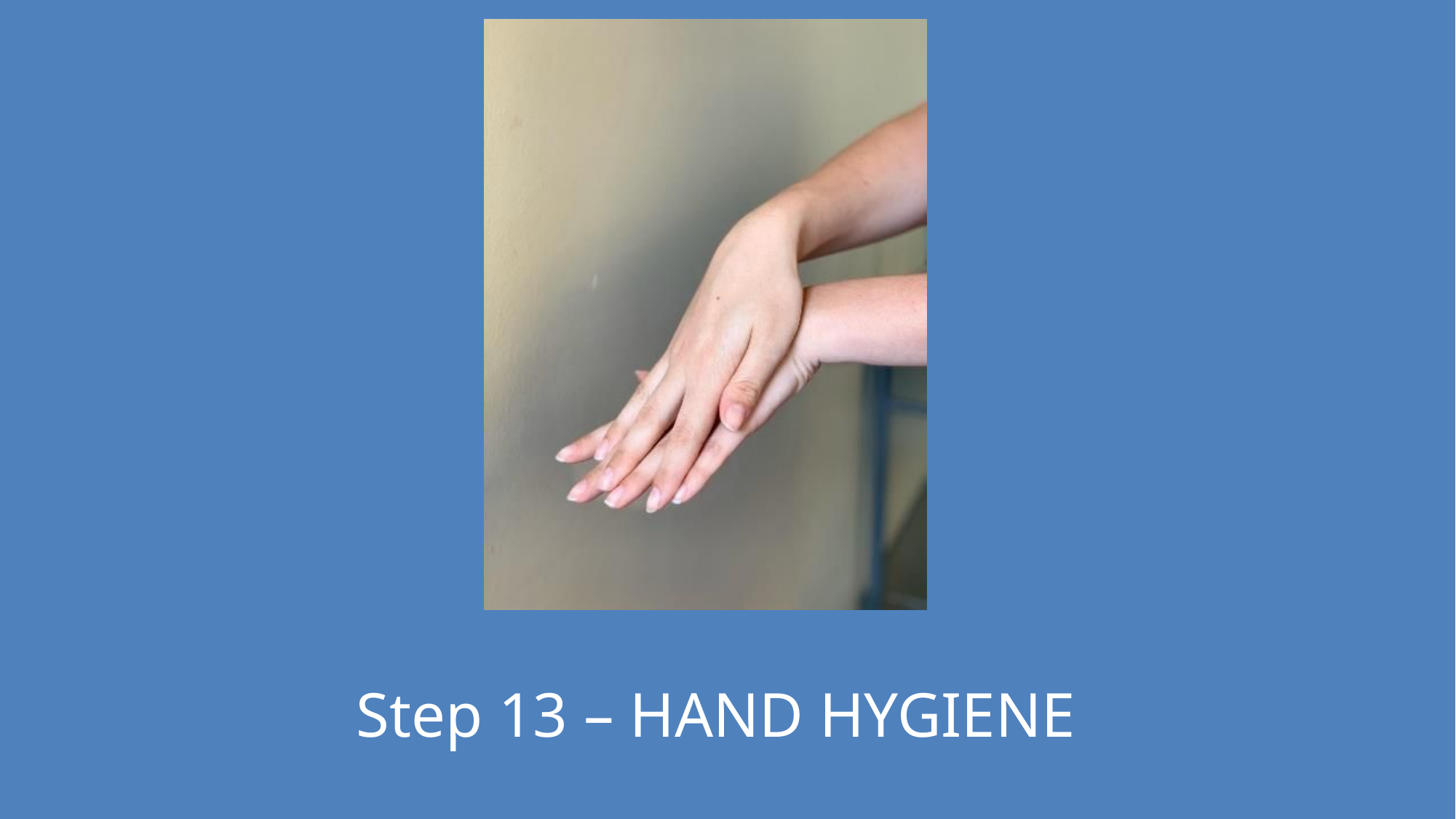

# Step 13 – HAND HYGIENE

## Slide 37
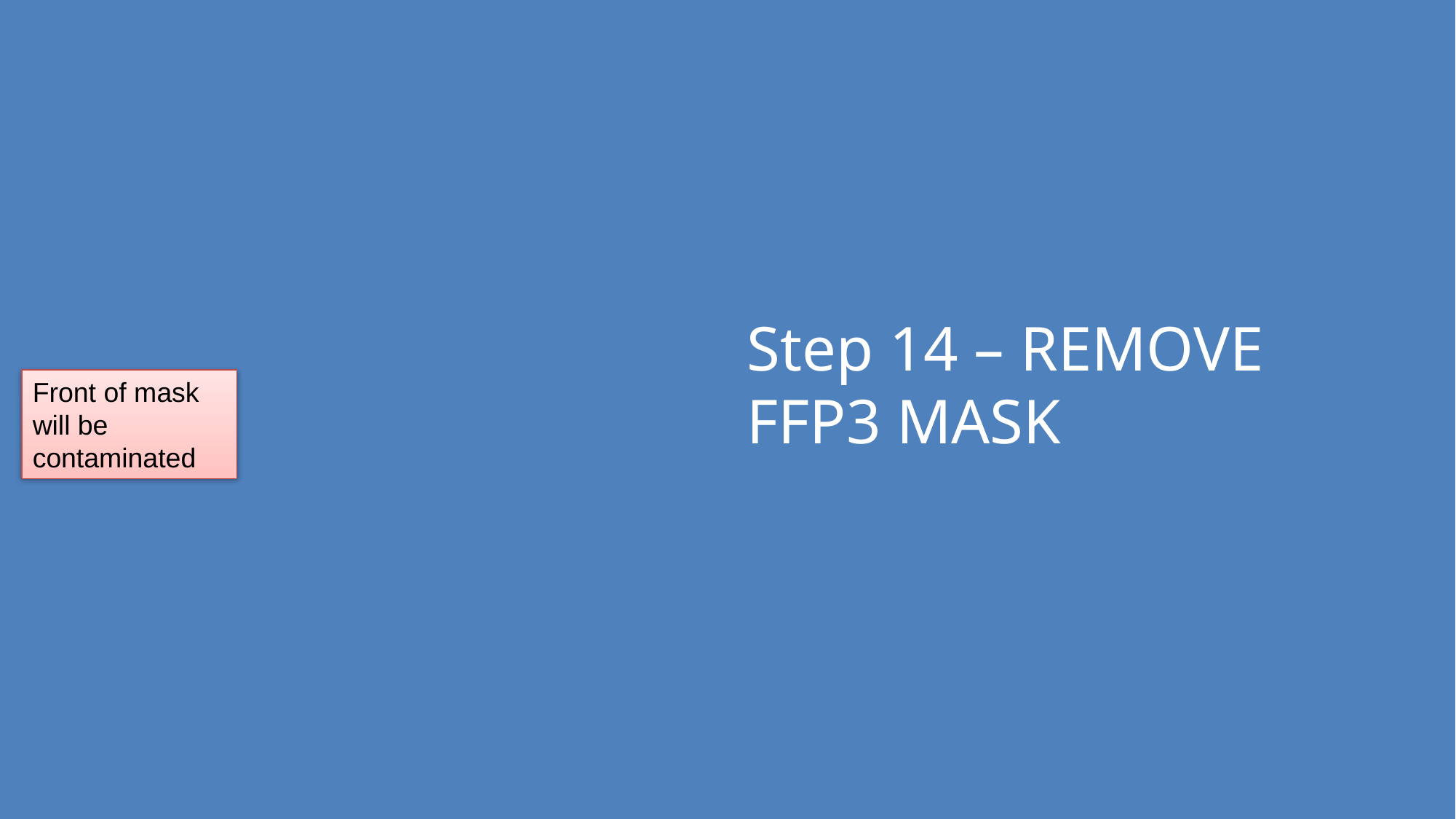

# Step 14 – REMOVE FFP3 MASK
Front of mask will be contaminated

## Slide 38
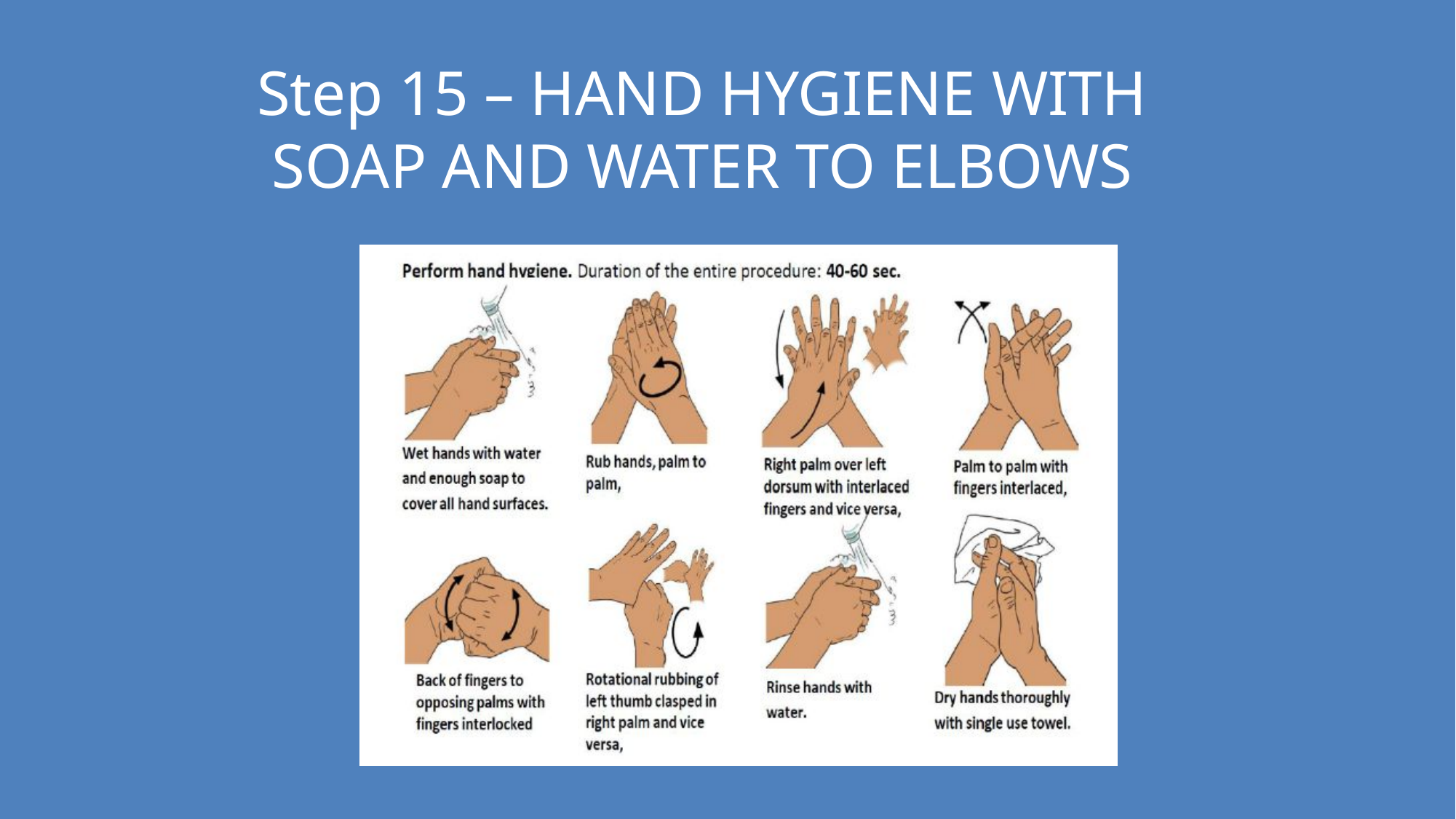

# Step 15 – HAND HYGIENE WITH SOAP AND WATER TO ELBOWS

## Slide 39
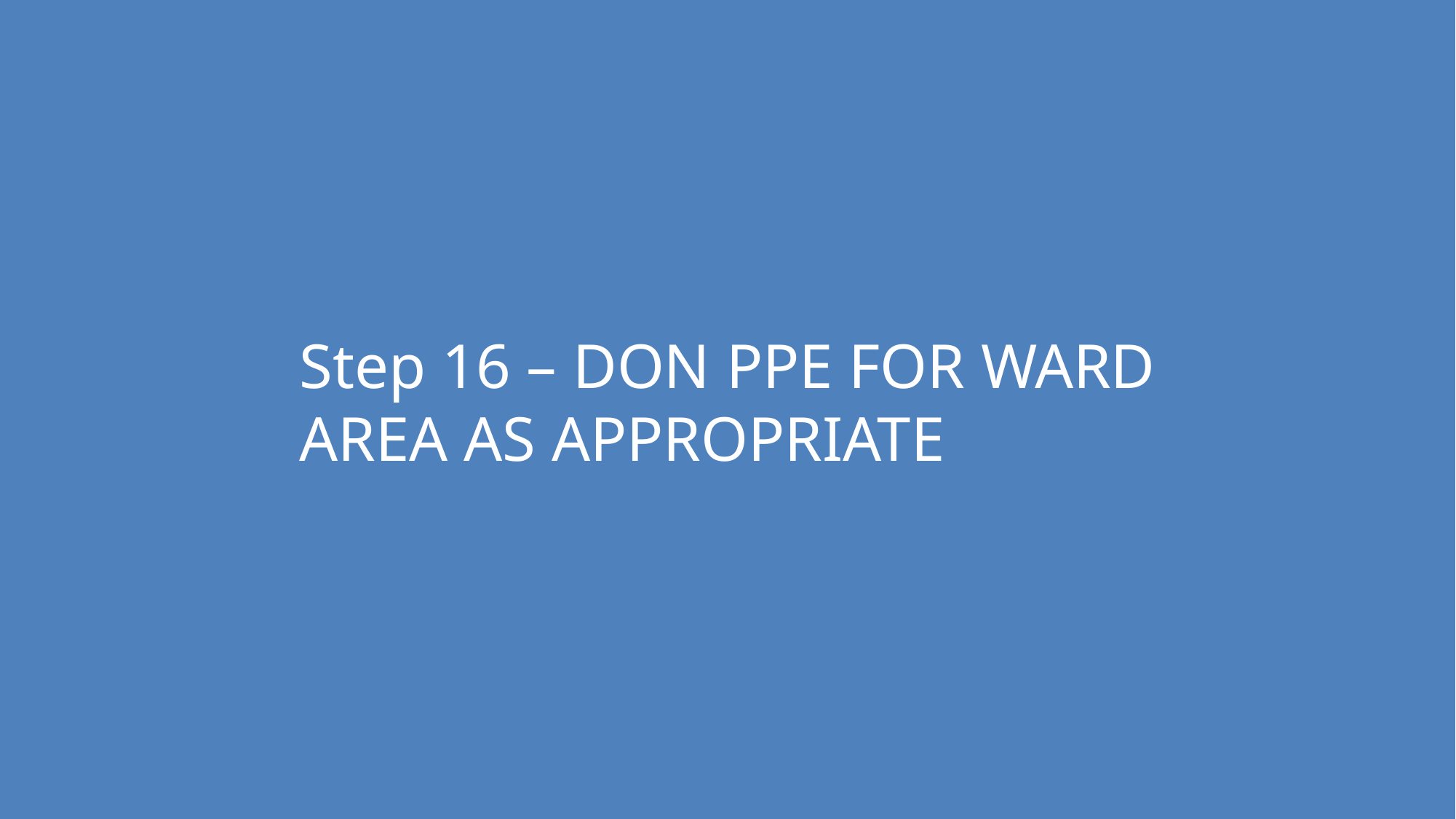

# Step 16 – DON PPE FOR WARD AREA AS APPROPRIATE

## Slide 40
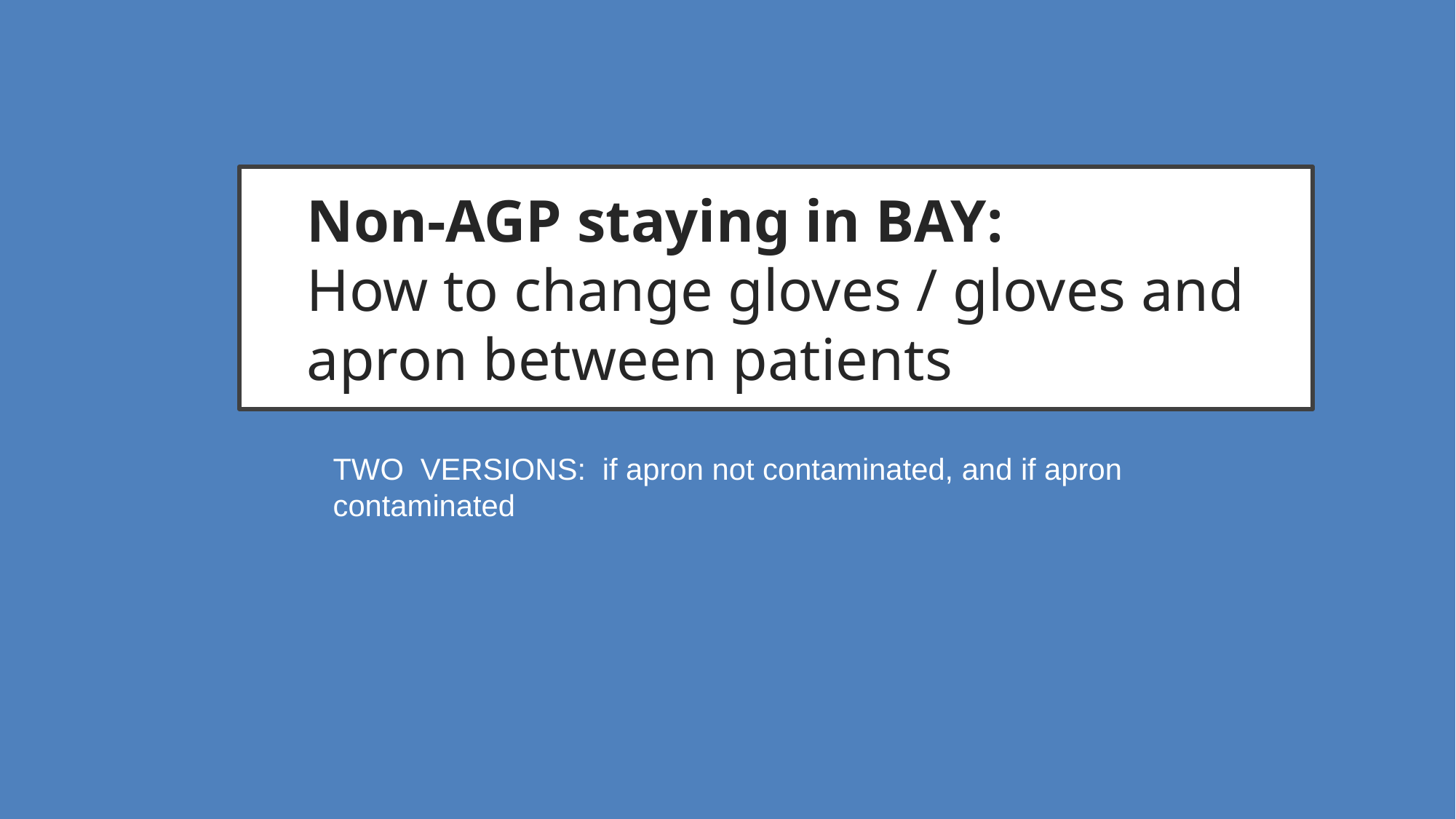

# Non-AGP staying in BAY: How to change gloves / gloves and apron between patients
TWO VERSIONS: if apron not contaminated, and if apron contaminated

## Slide 41
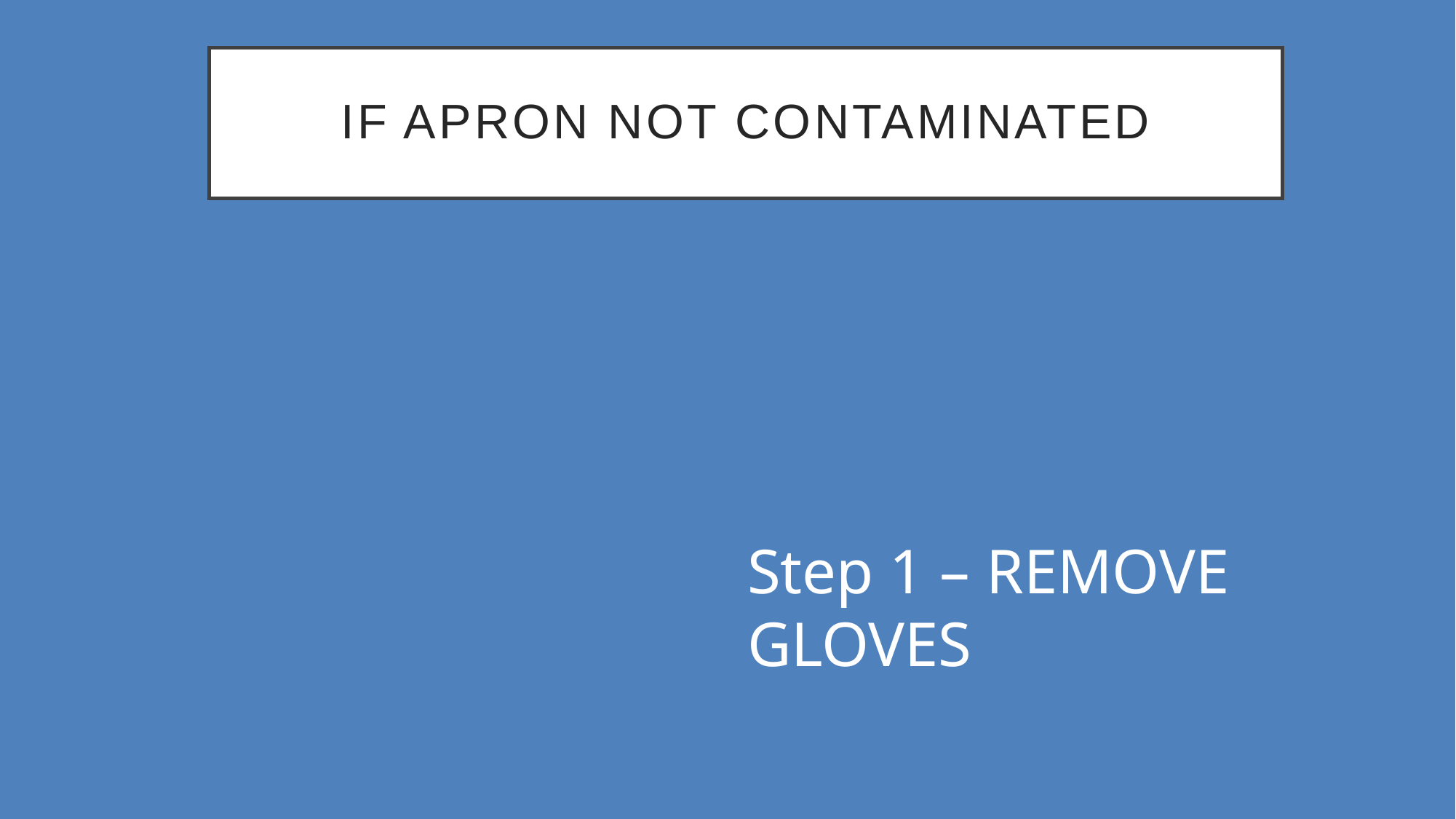

IF APRON NOT CONTAMINATED
# Step 1 – REMOVE GLOVES

## Slide 42
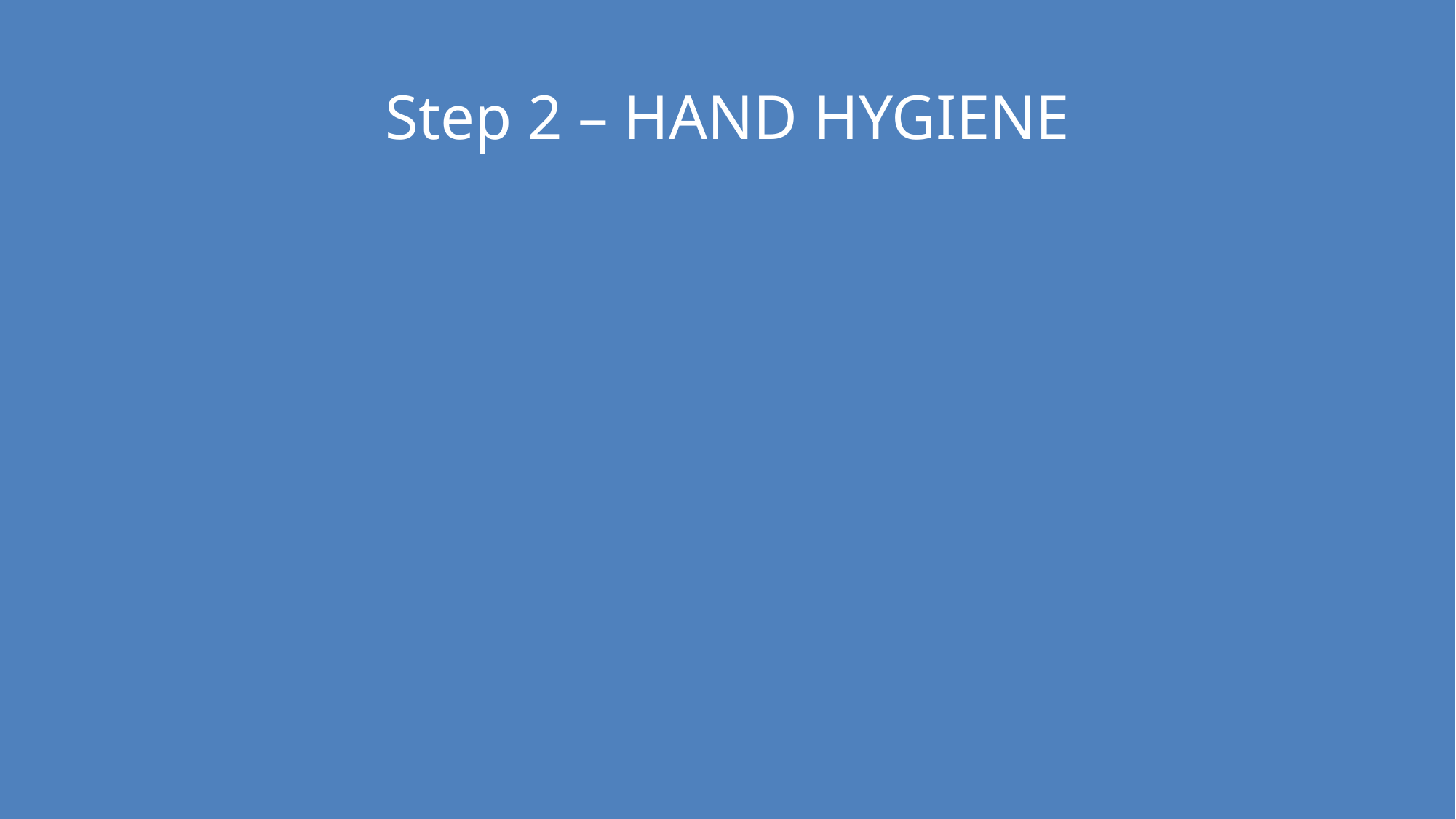

# Step 2 – HAND HYGIENE

## Slide 43
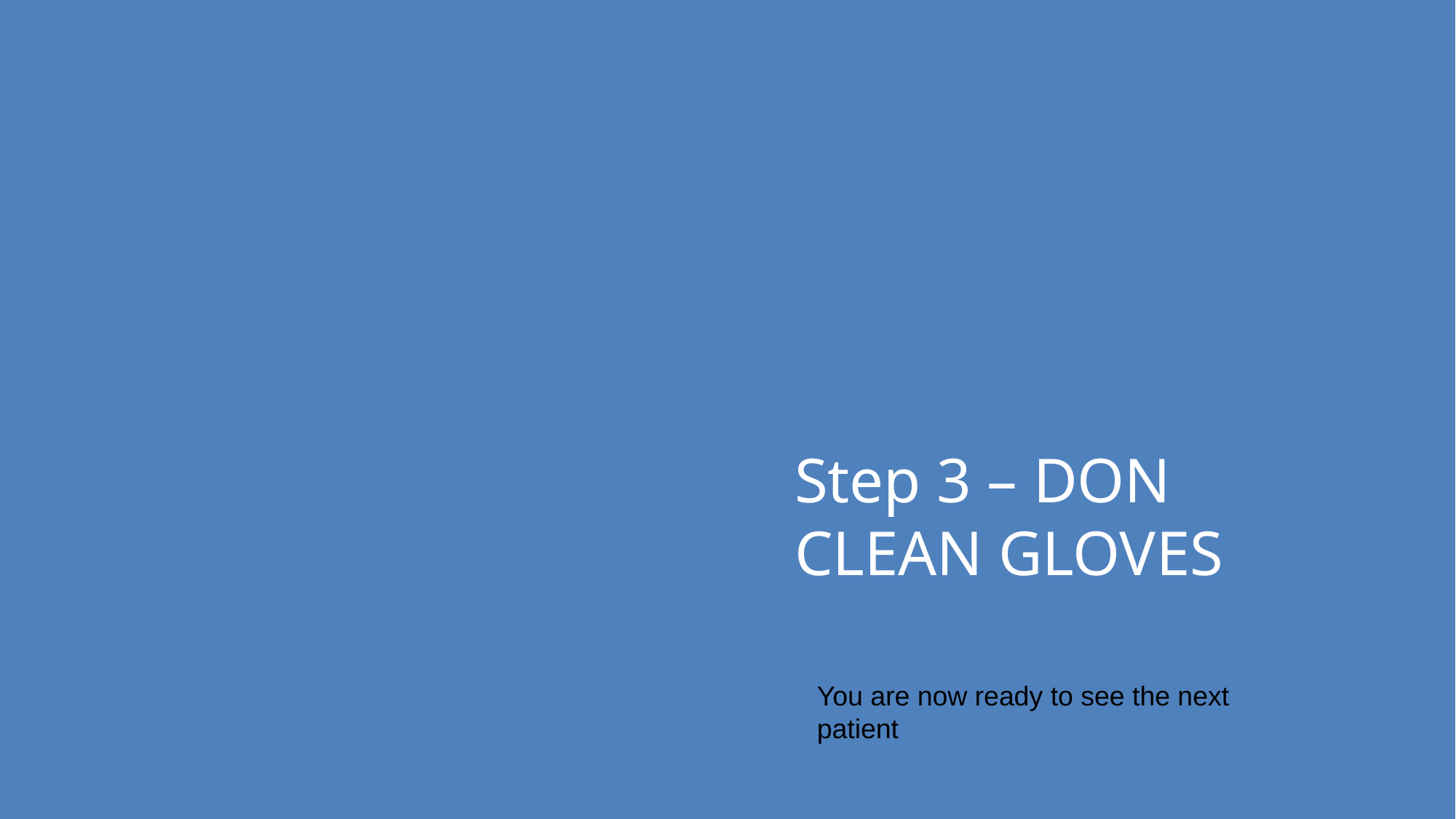

# Step 3 – DON CLEAN GLOVES
You are now ready to see the next patient

## Slide 44
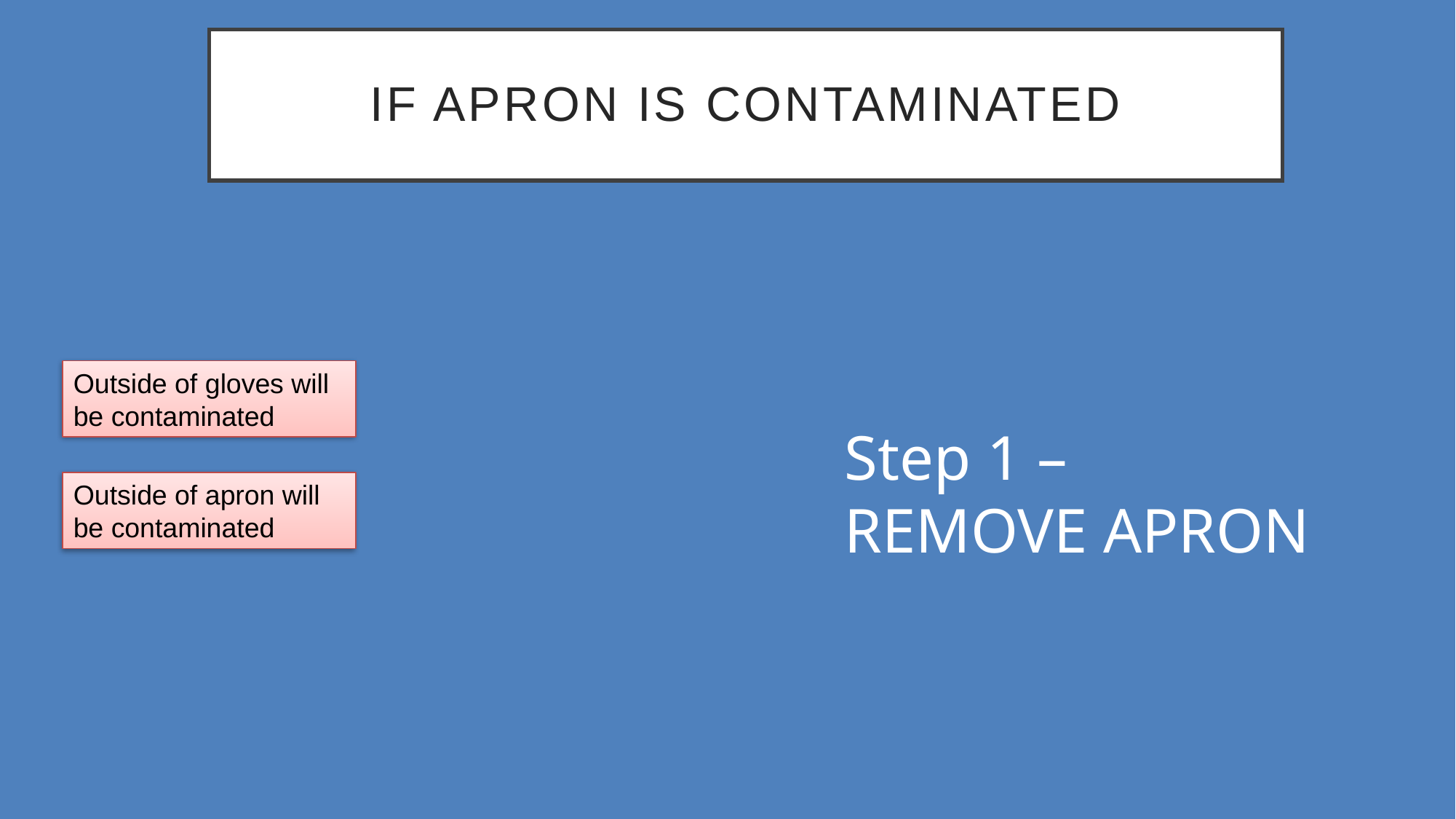

IF APRON IS CONTAMINATED
Outside of gloves will be contaminated
# Step 1 – REMOVE APRON
Outside of apron will be contaminated

## Slide 45
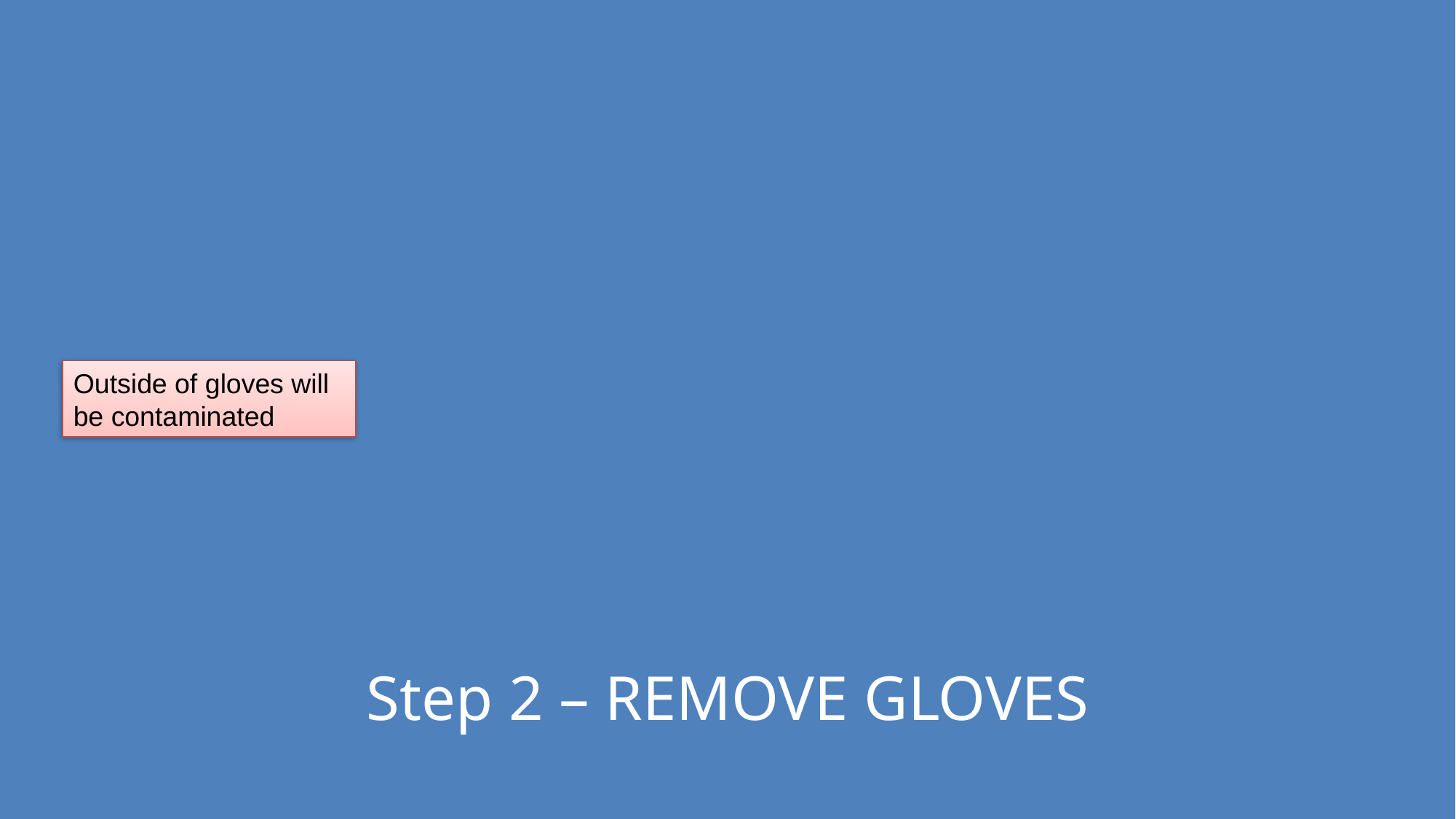

Outside of gloves will be contaminated
# Step 2 – REMOVE GLOVES

## Slide 46
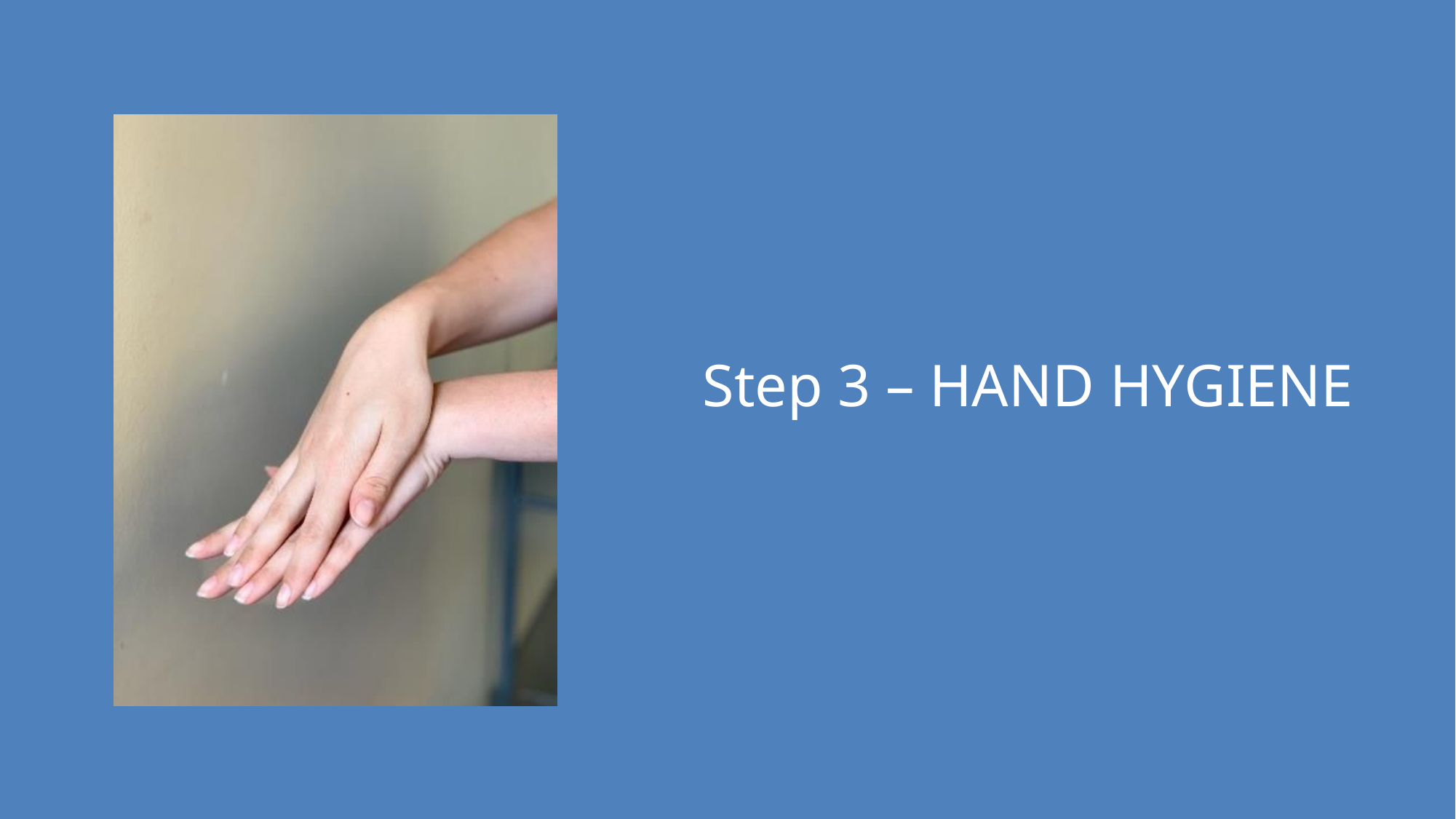

# Step 3 – HAND HYGIENE

## Slide 47
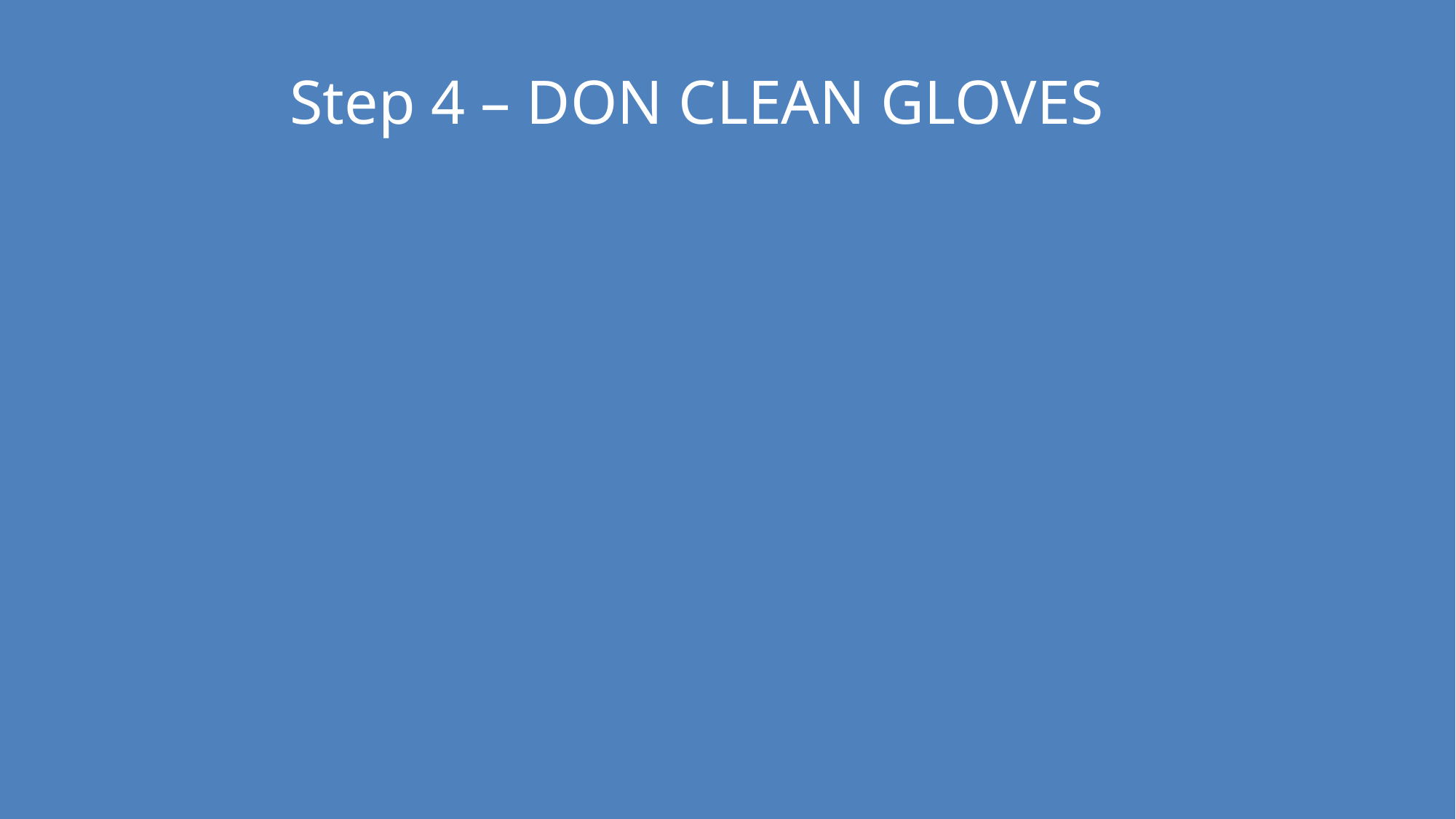

# Step 4 – DON CLEAN GLOVES

## Slide 48
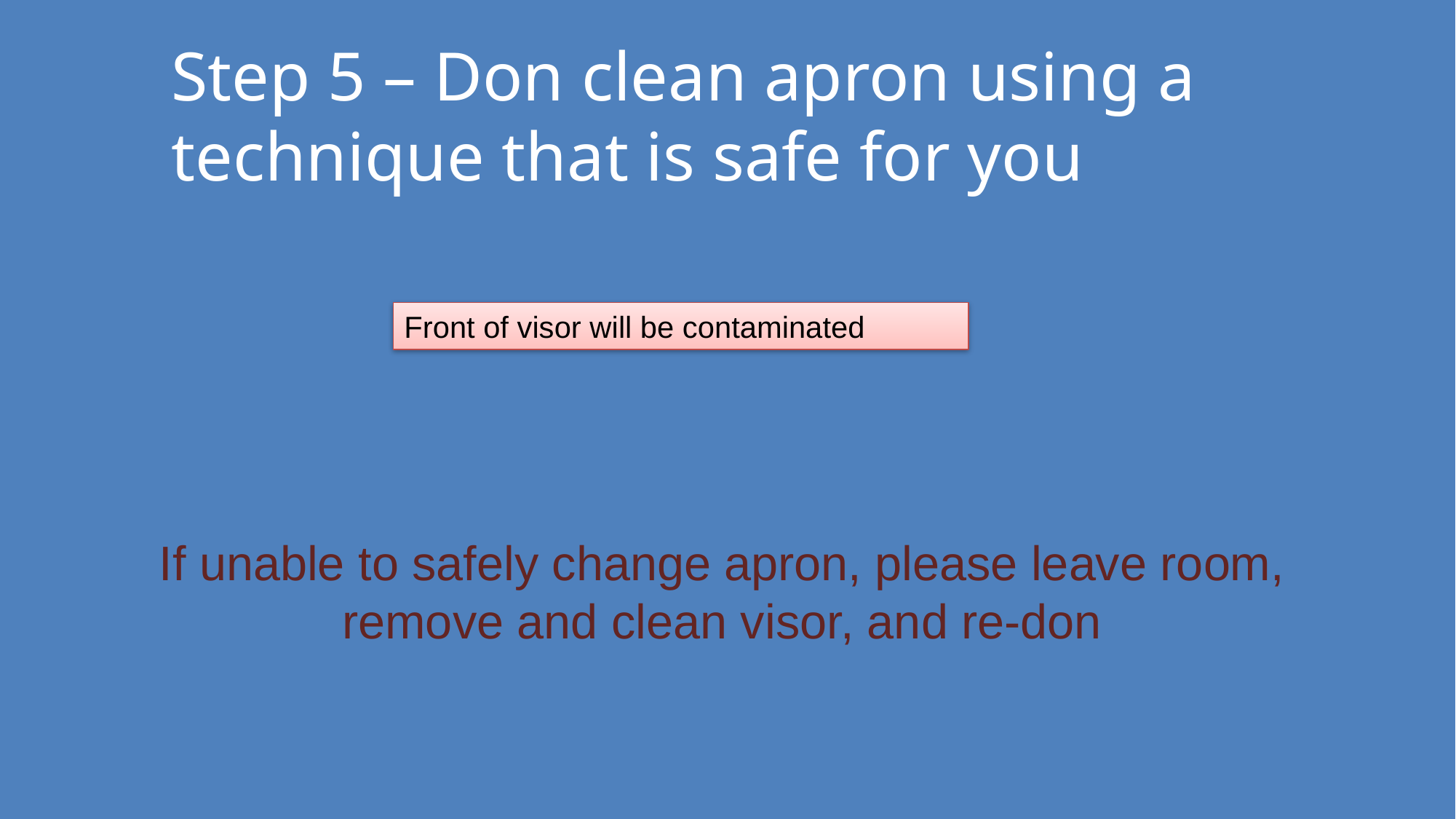

# Step 5 – Don clean apron using a technique that is safe for you
Front of visor will be contaminated
If unable to safely change apron, please leave room, remove and clean visor, and re-don

## Slide 49
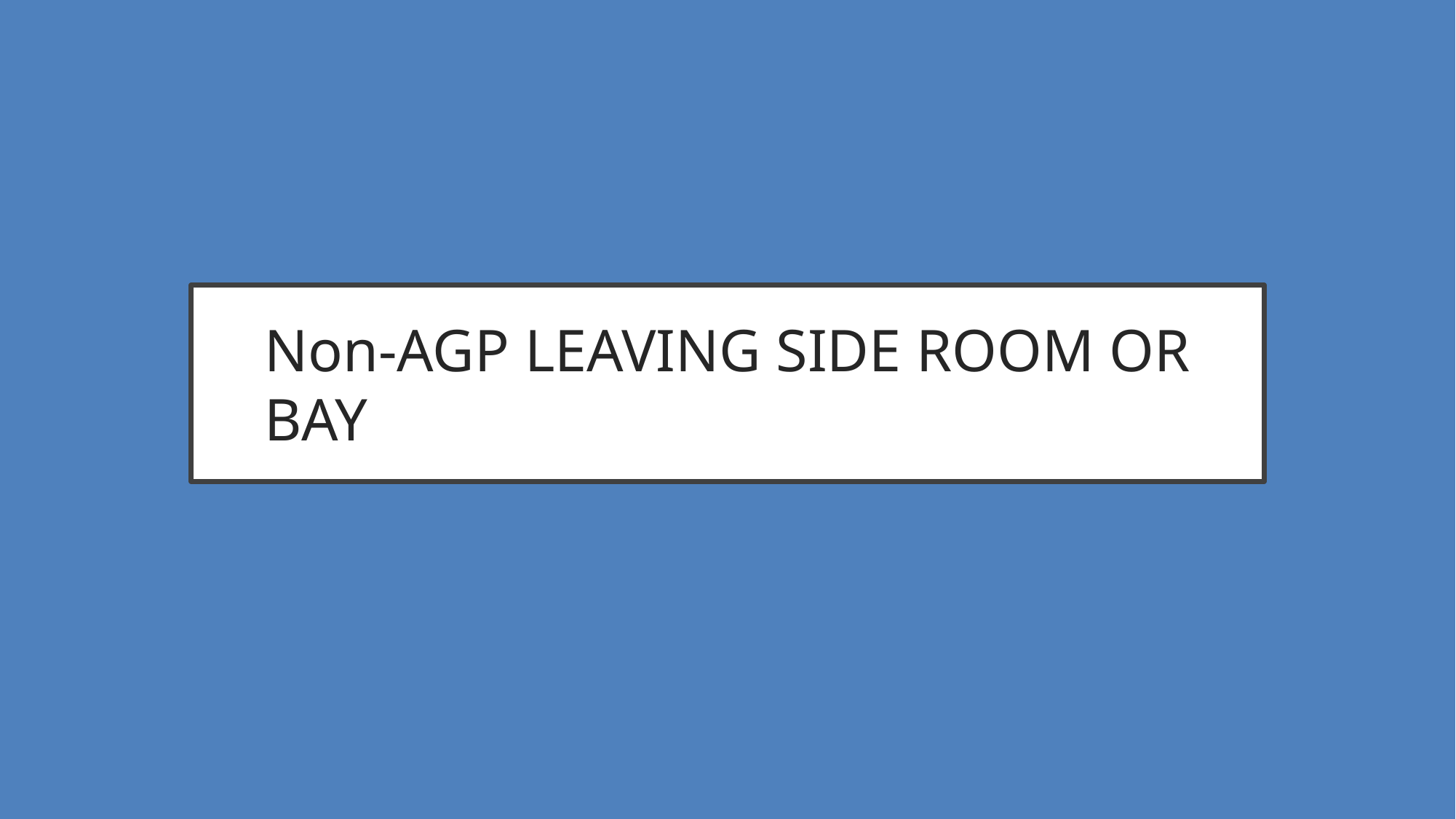

# Non-AGP LEAVING SIDE ROOM OR BAY

## Slide 50
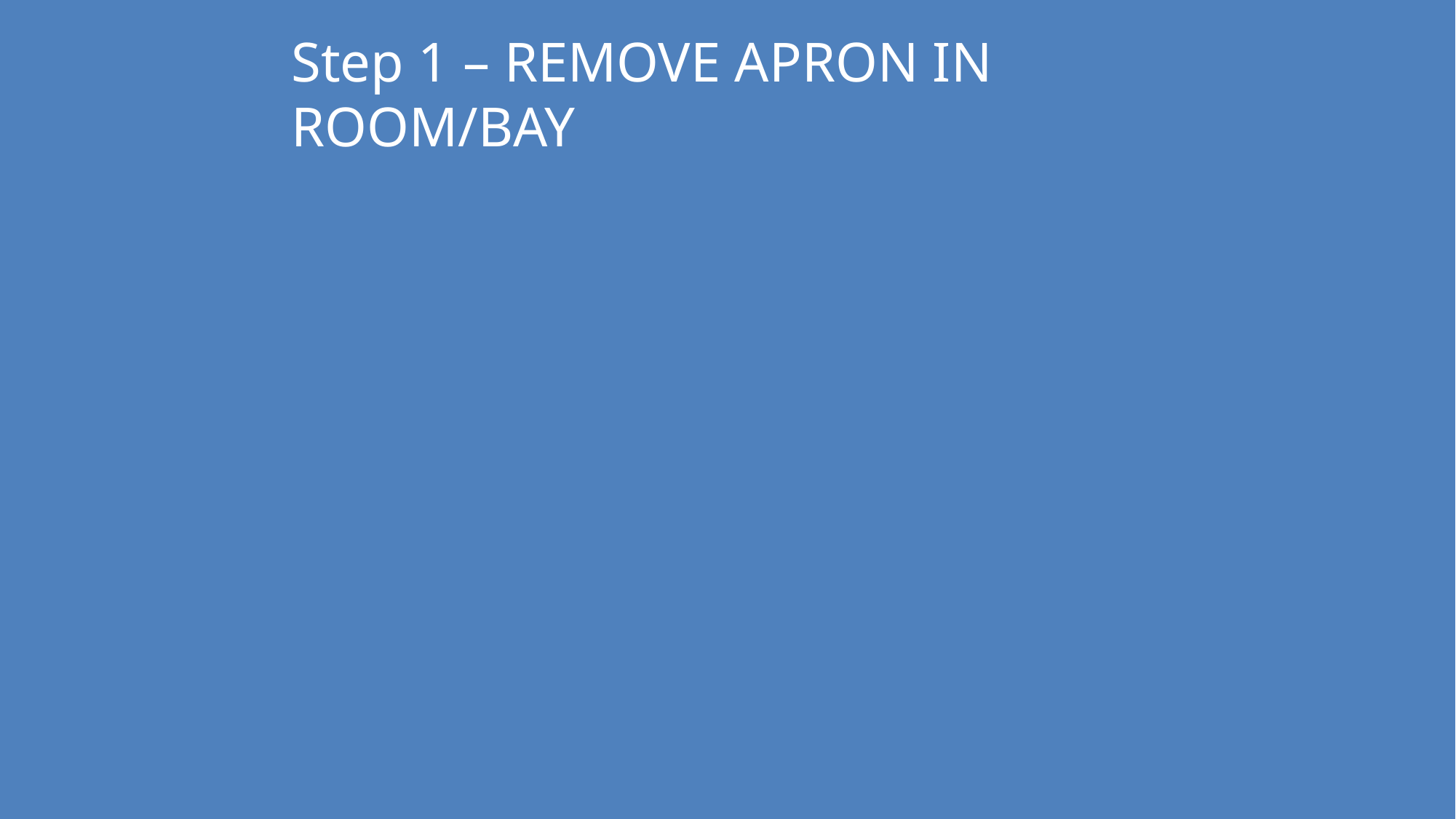

# Step 1 – REMOVE APRON IN ROOM/BAY

## Slide 51
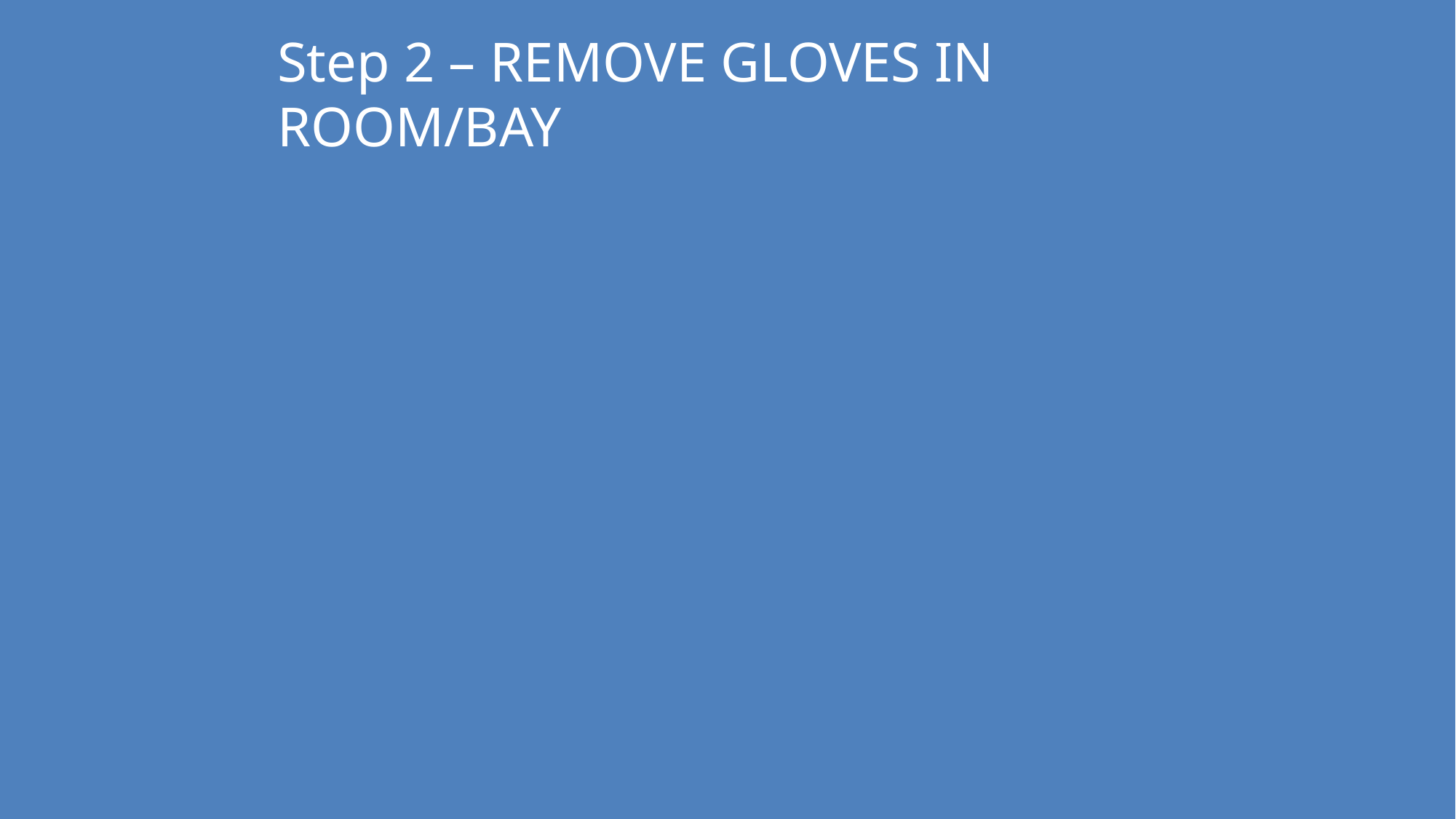

# Step 2 – REMOVE GLOVES IN ROOM/BAY

## Slide 52
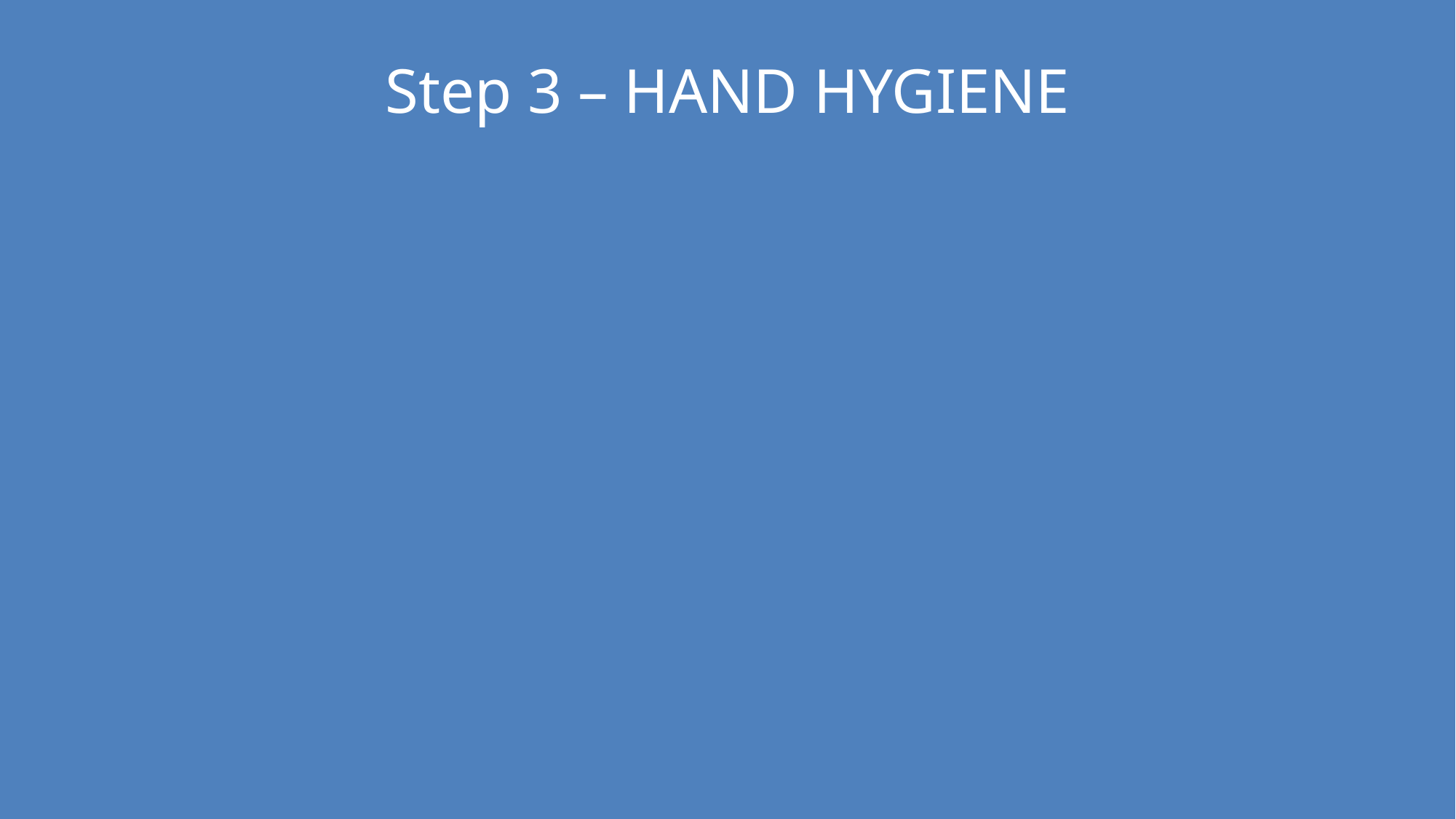

# Step 3 – HAND HYGIENE

## Slide 53
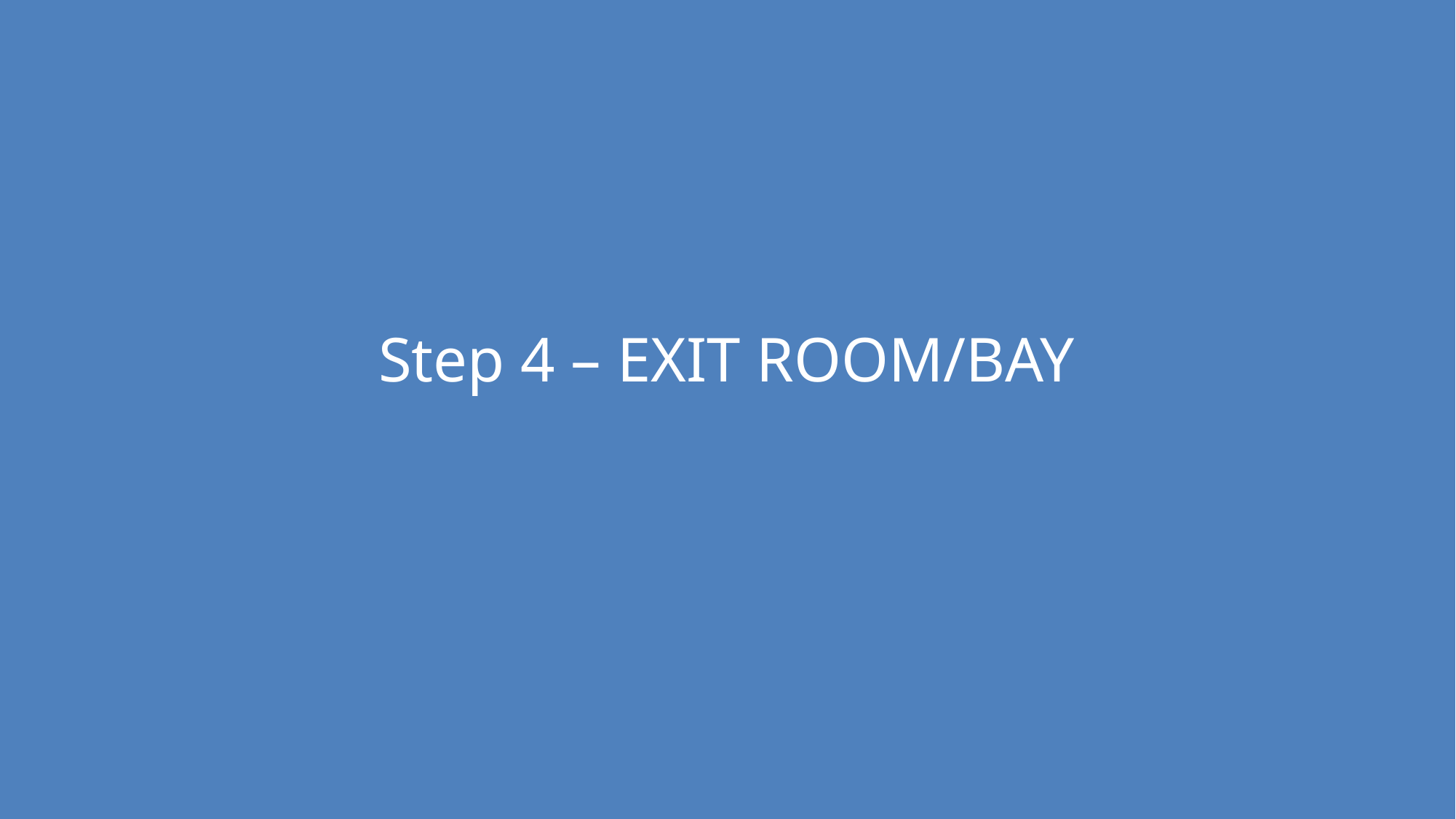

# Step 4 – EXIT ROOM/BAY

## Slide 54
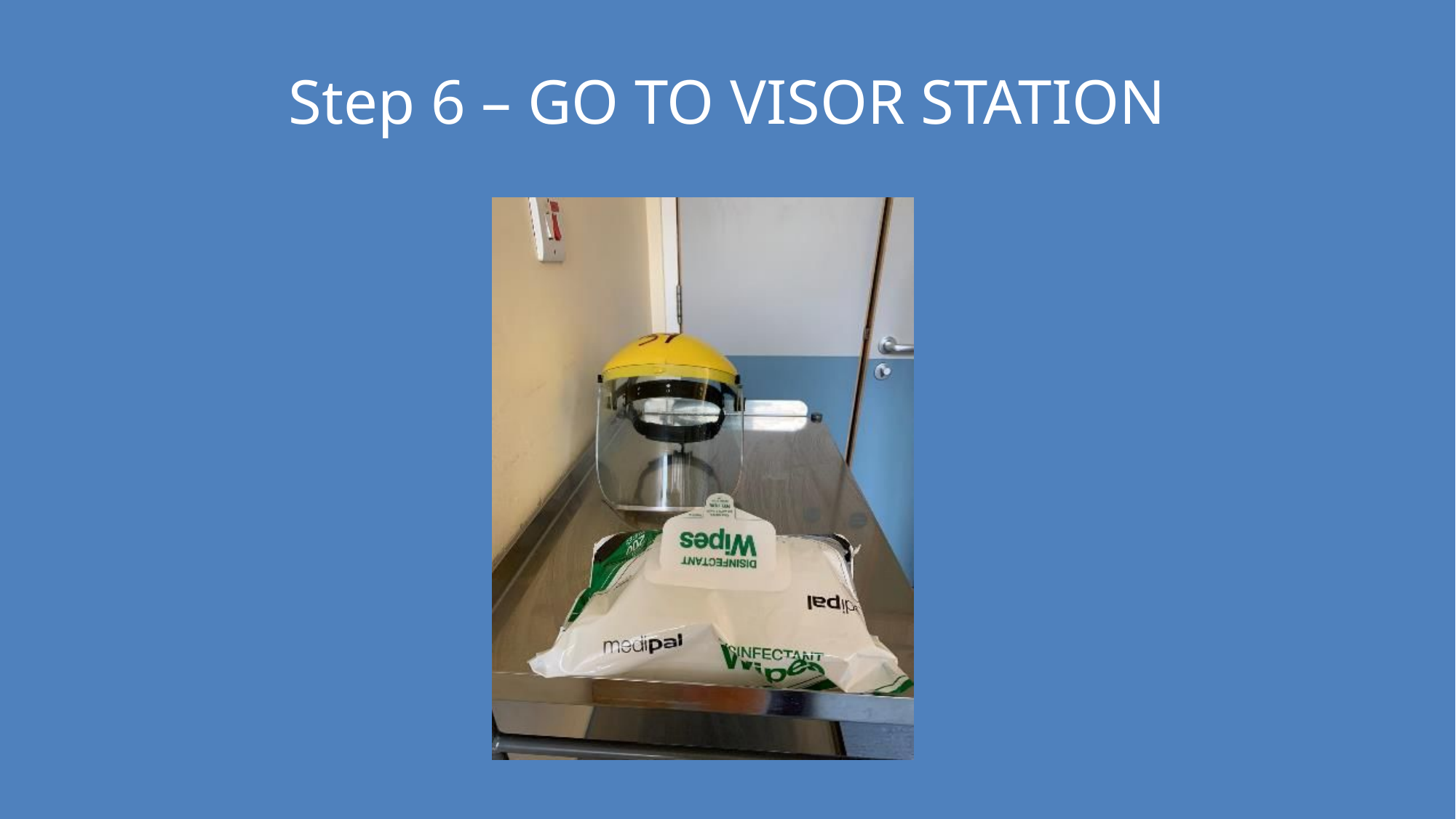

# Step 6 – GO TO VISOR STATION

## Slide 55
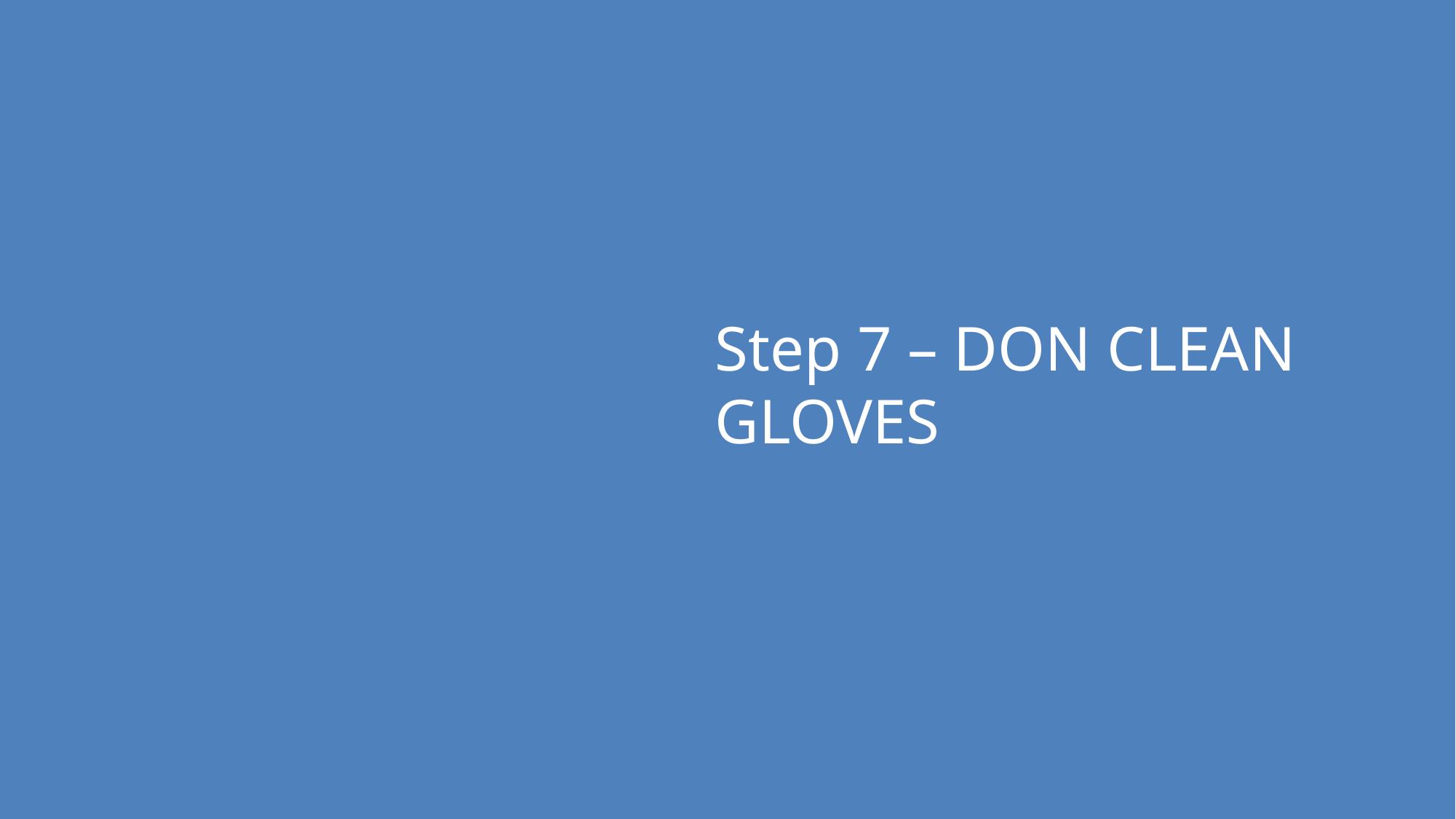

# Step 7 – DON CLEAN GLOVES

## Slide 56
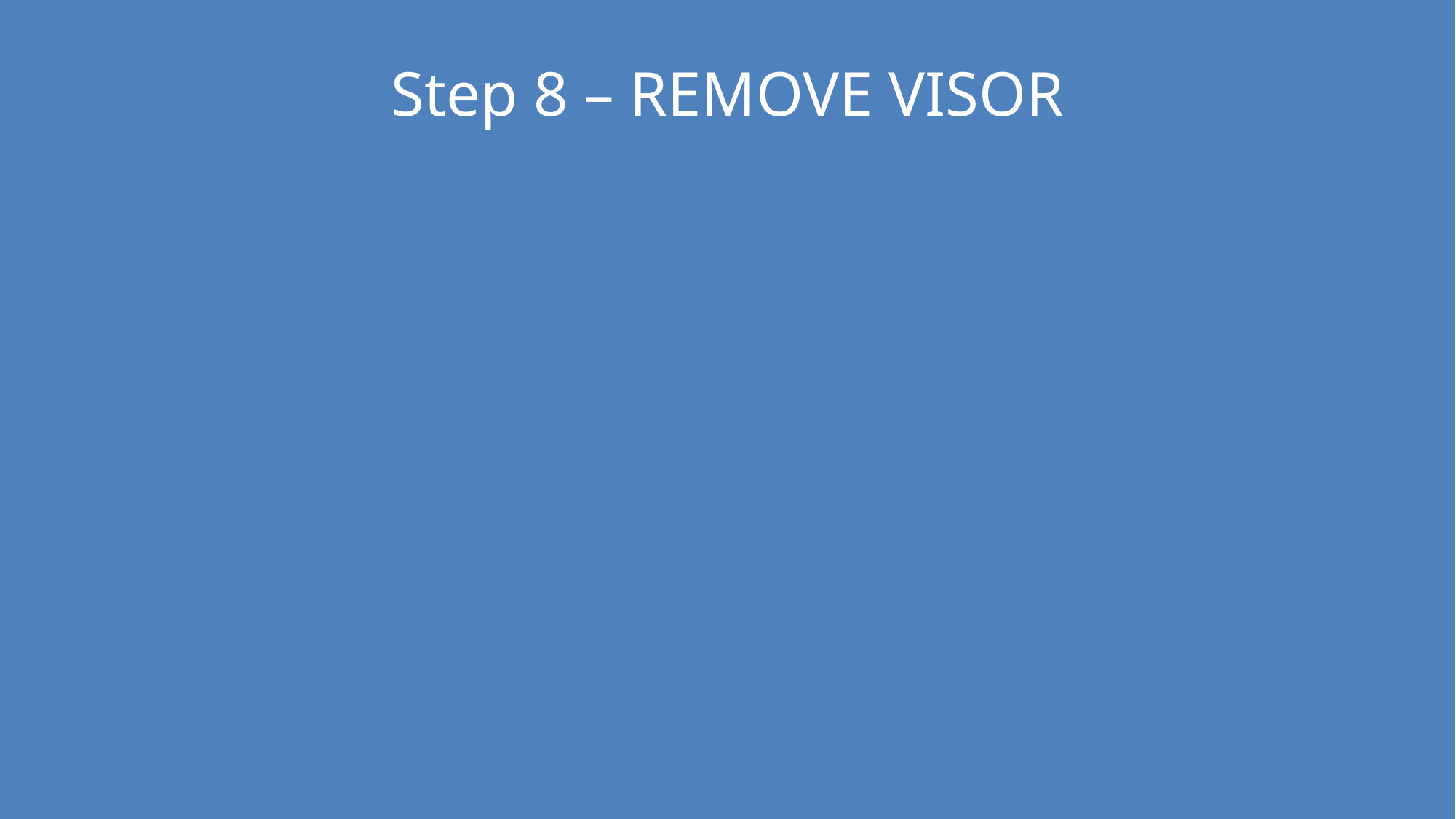

# Step 8 – REMOVE VISOR

## Slide 57
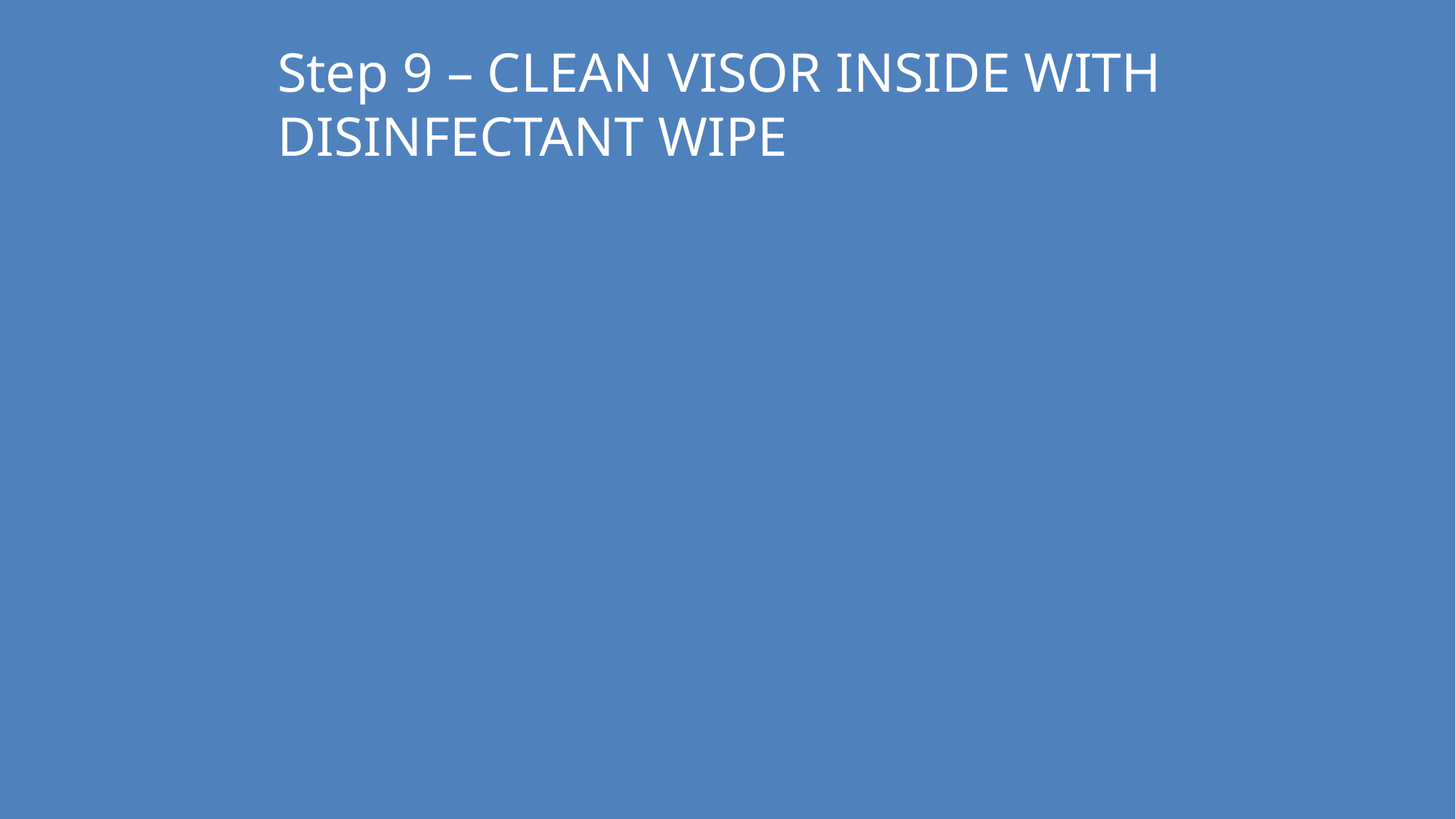

# Step 9 – CLEAN VISOR INSIDE WITH DISINFECTANT WIPE

## Slide 58
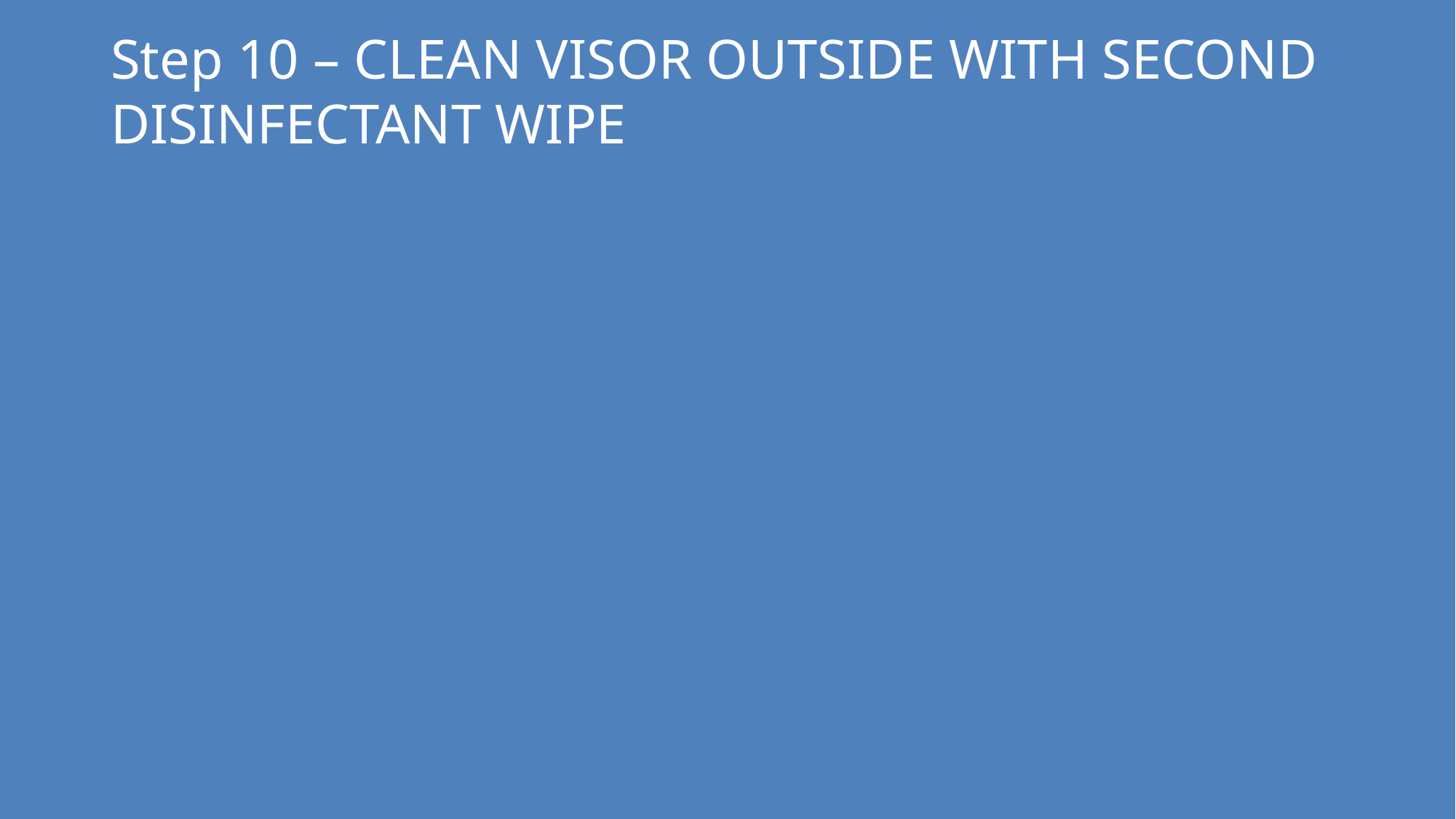

# Step 10 – CLEAN VISOR OUTSIDE WITH SECOND DISINFECTANT WIPE

## Slide 59
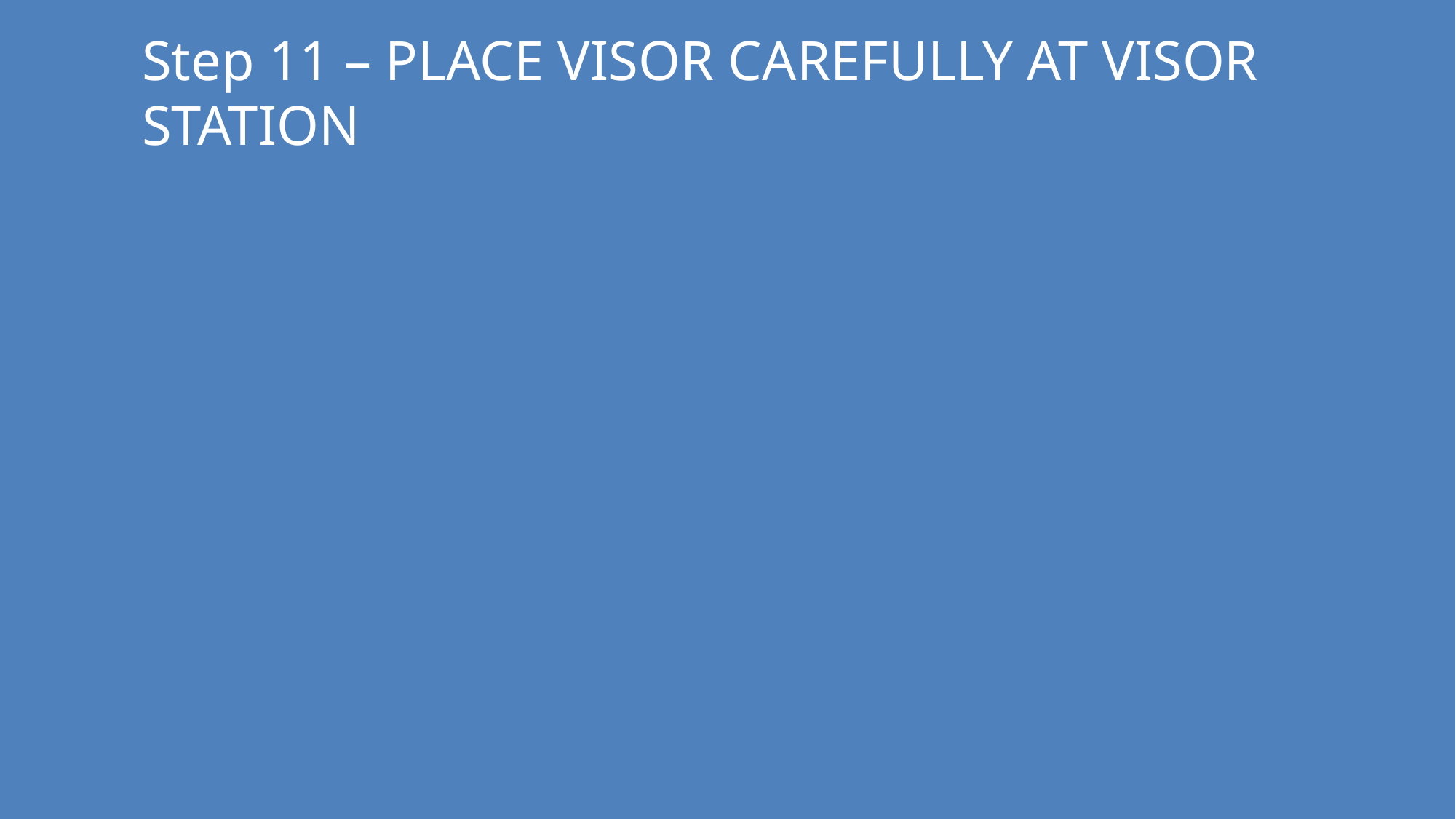

# Step 11 – PLACE VISOR CAREFULLY AT VISOR STATION

## Slide 60
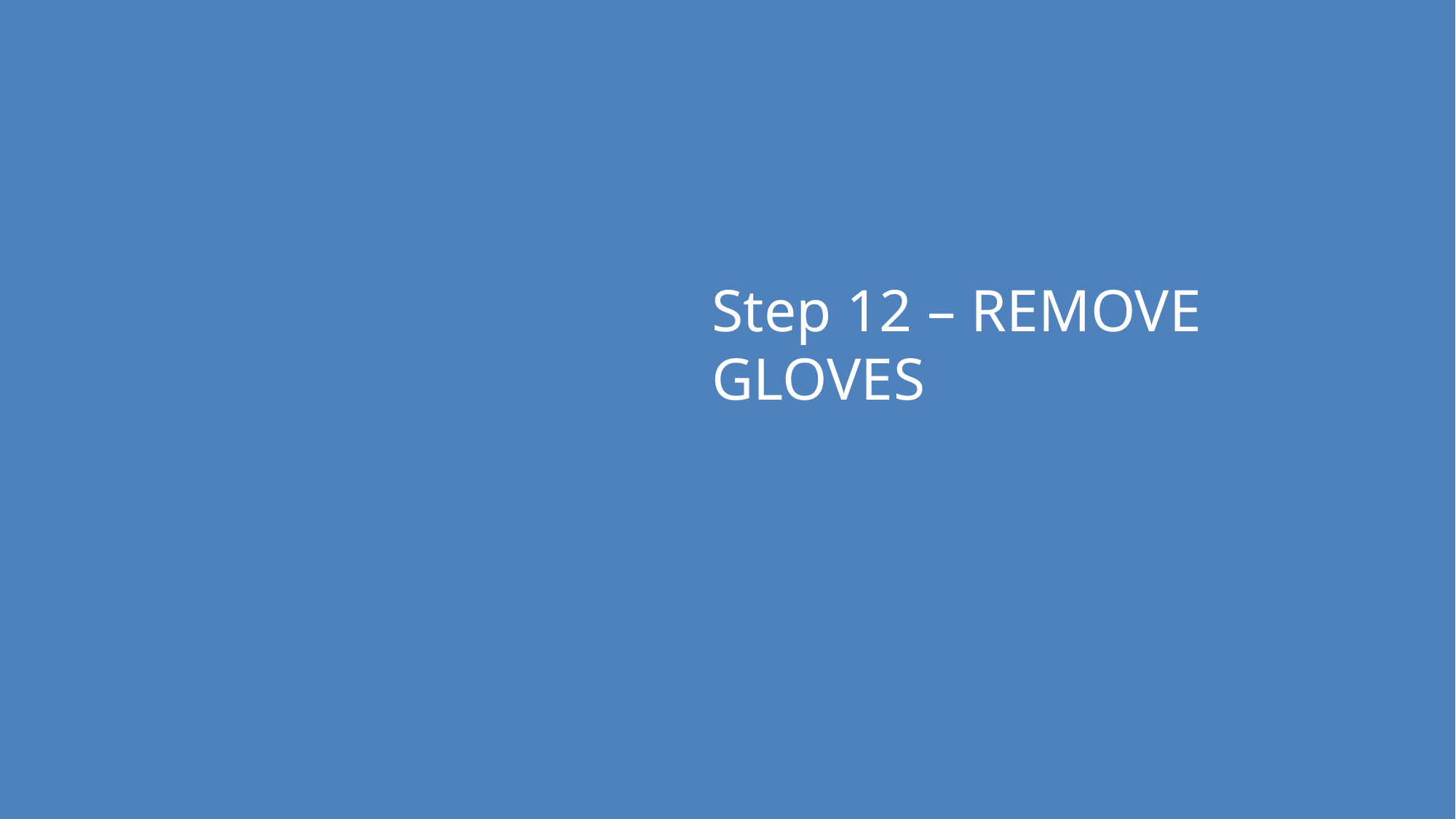

# Step 12 – REMOVE GLOVES

## Slide 61
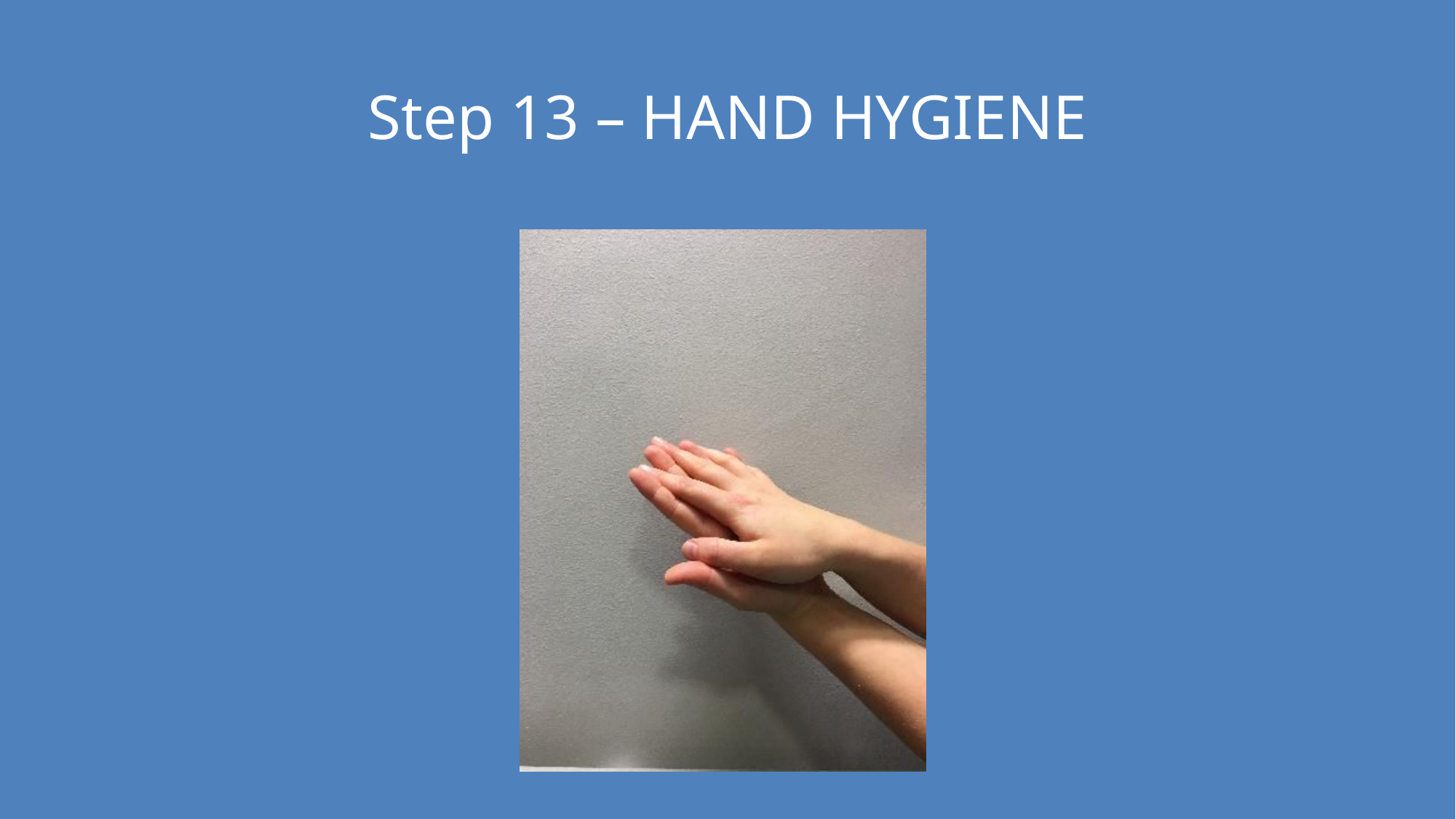

# Step 13 – HAND HYGIENE

## Slide 62
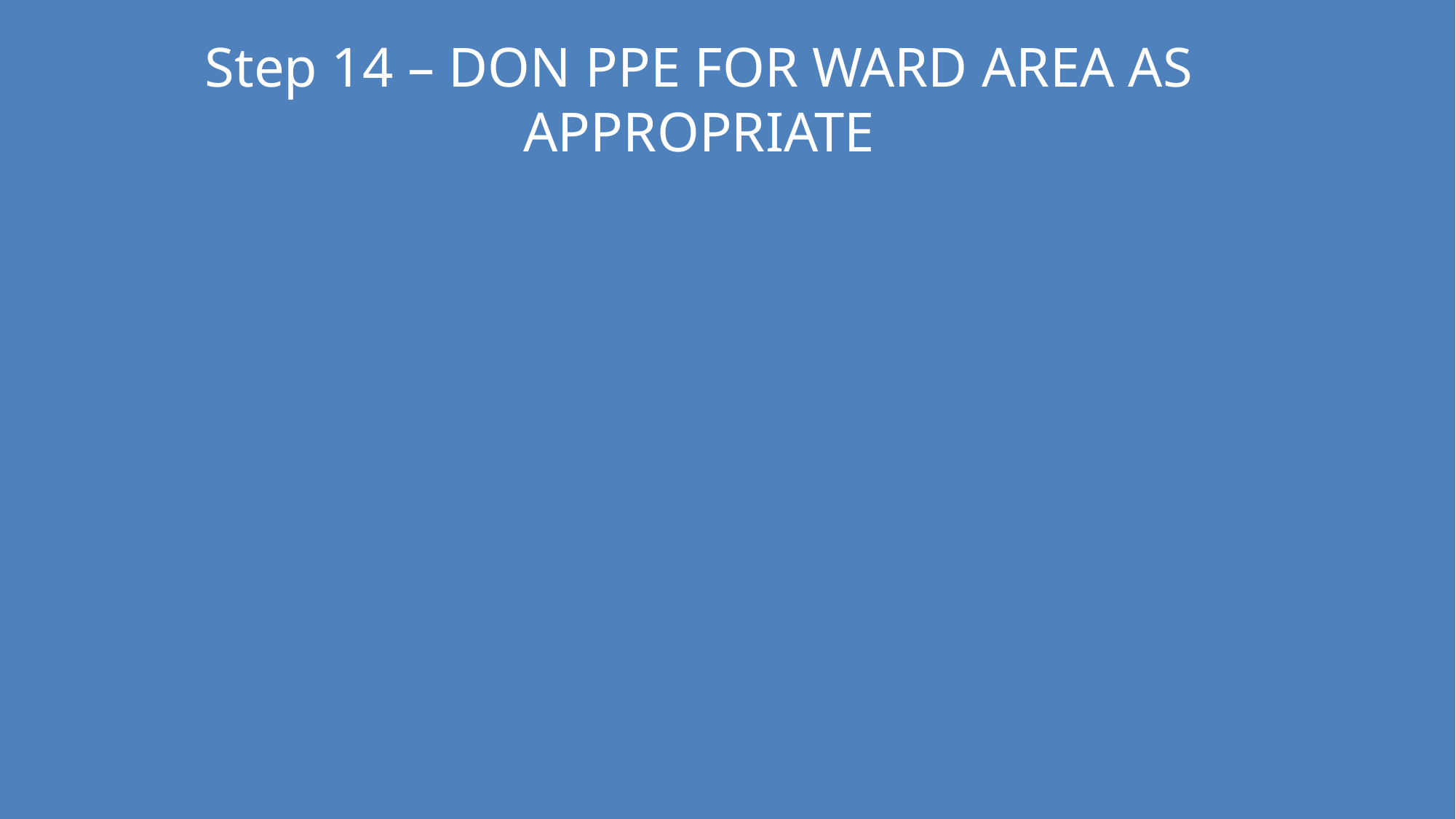

# Step 14 – DON PPE FOR WARD AREA AS APPROPRIATE

## Slide 63
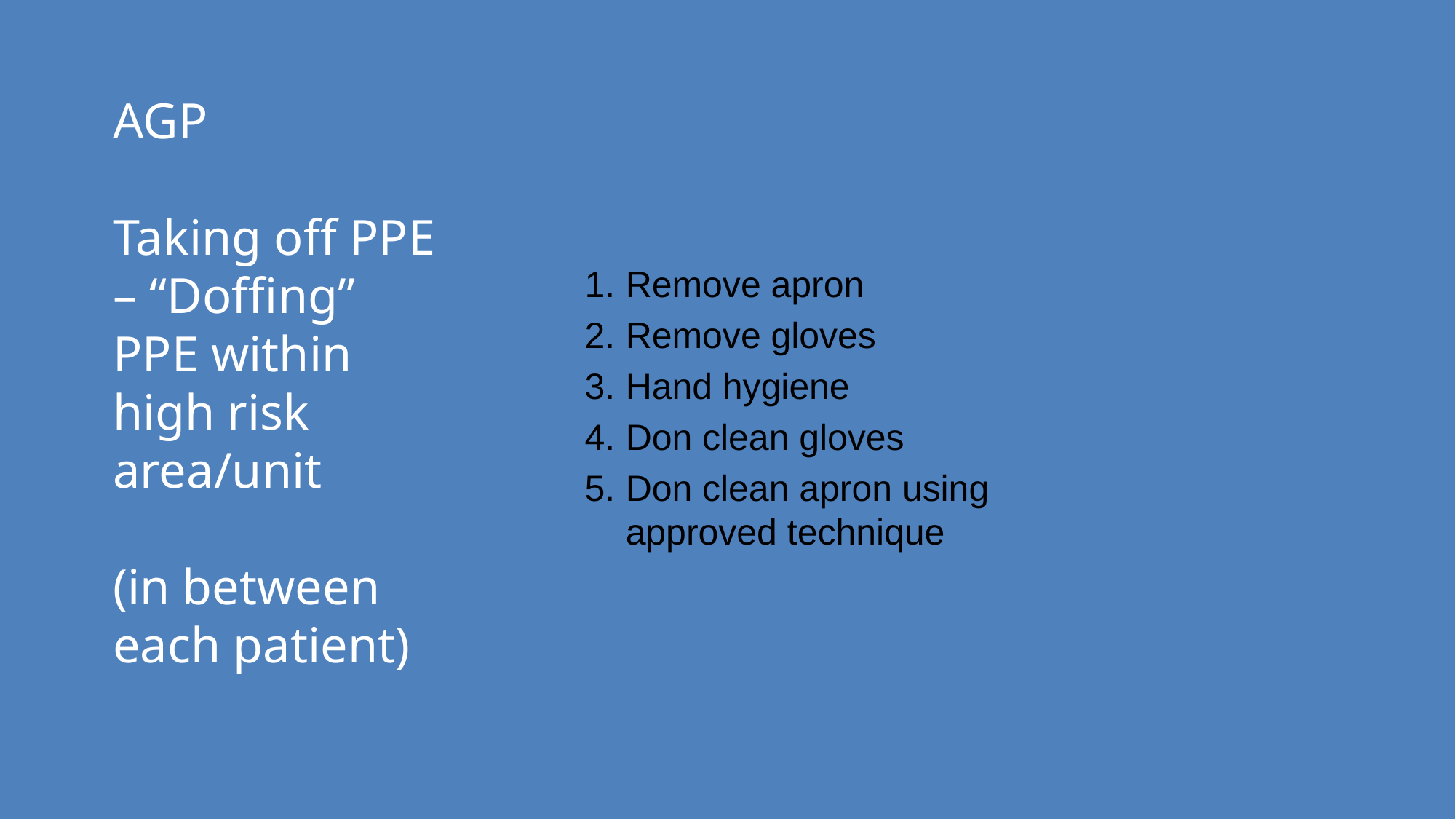

# AGP Taking off PPE – “Doffing” PPE within high risk area/unit (in between each patient)
Remove apron
Remove gloves
Hand hygiene
Don clean gloves
Don clean apron using approved technique

## Slide 64
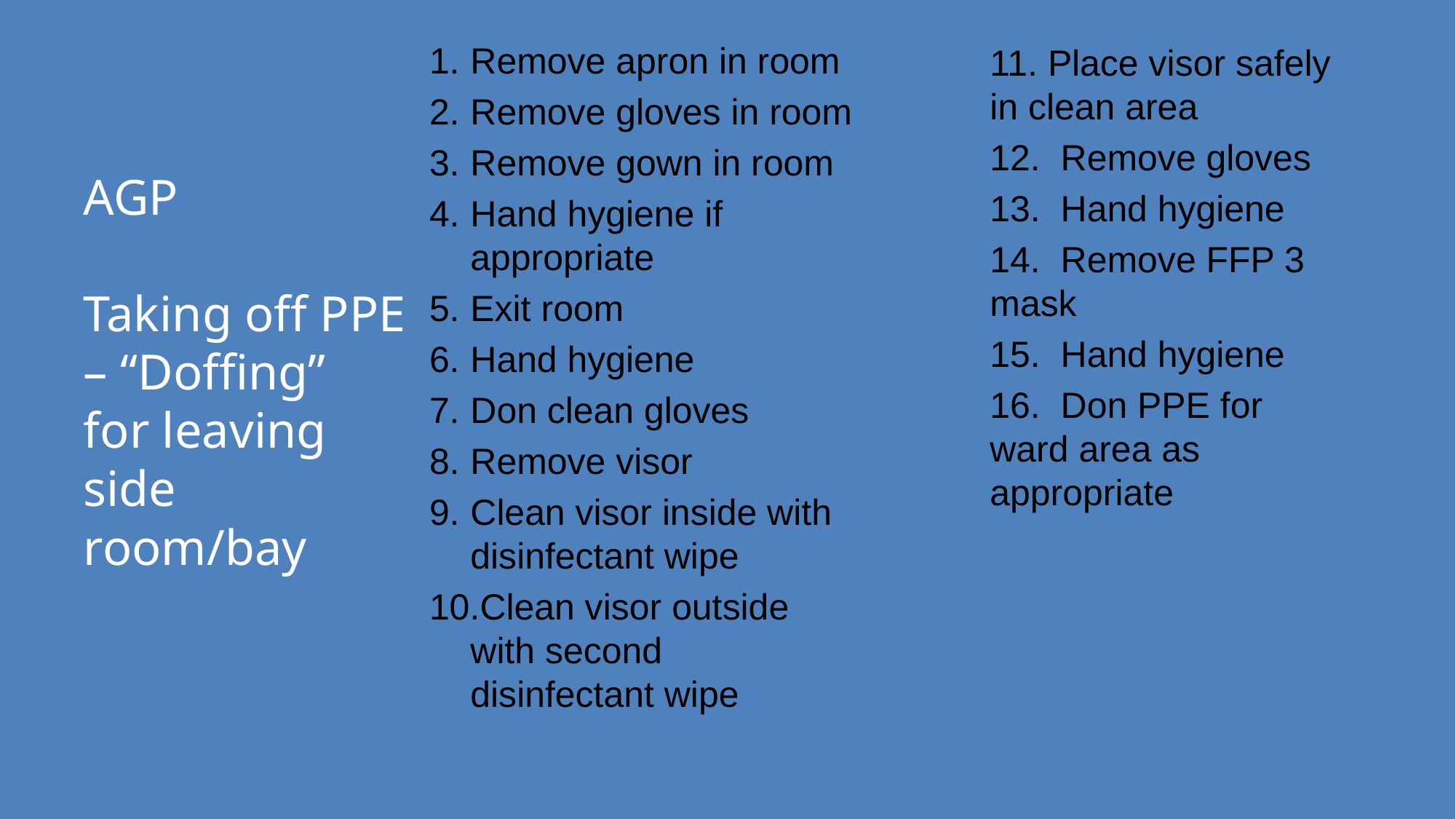

Remove apron in room
Remove gloves in room
Remove gown in room
Hand hygiene if appropriate
Exit room
Hand hygiene
Don clean gloves
Remove visor
Clean visor inside with disinfectant wipe
Clean visor outside with second disinfectant wipe
11. Place visor safely in clean area
12. Remove gloves
13. Hand hygiene
14. Remove FFP 3 mask
15. Hand hygiene
16. Don PPE for ward area as appropriate
# AGP Taking off PPE – “Doffing” for leaving side room/bay

## Slide 65
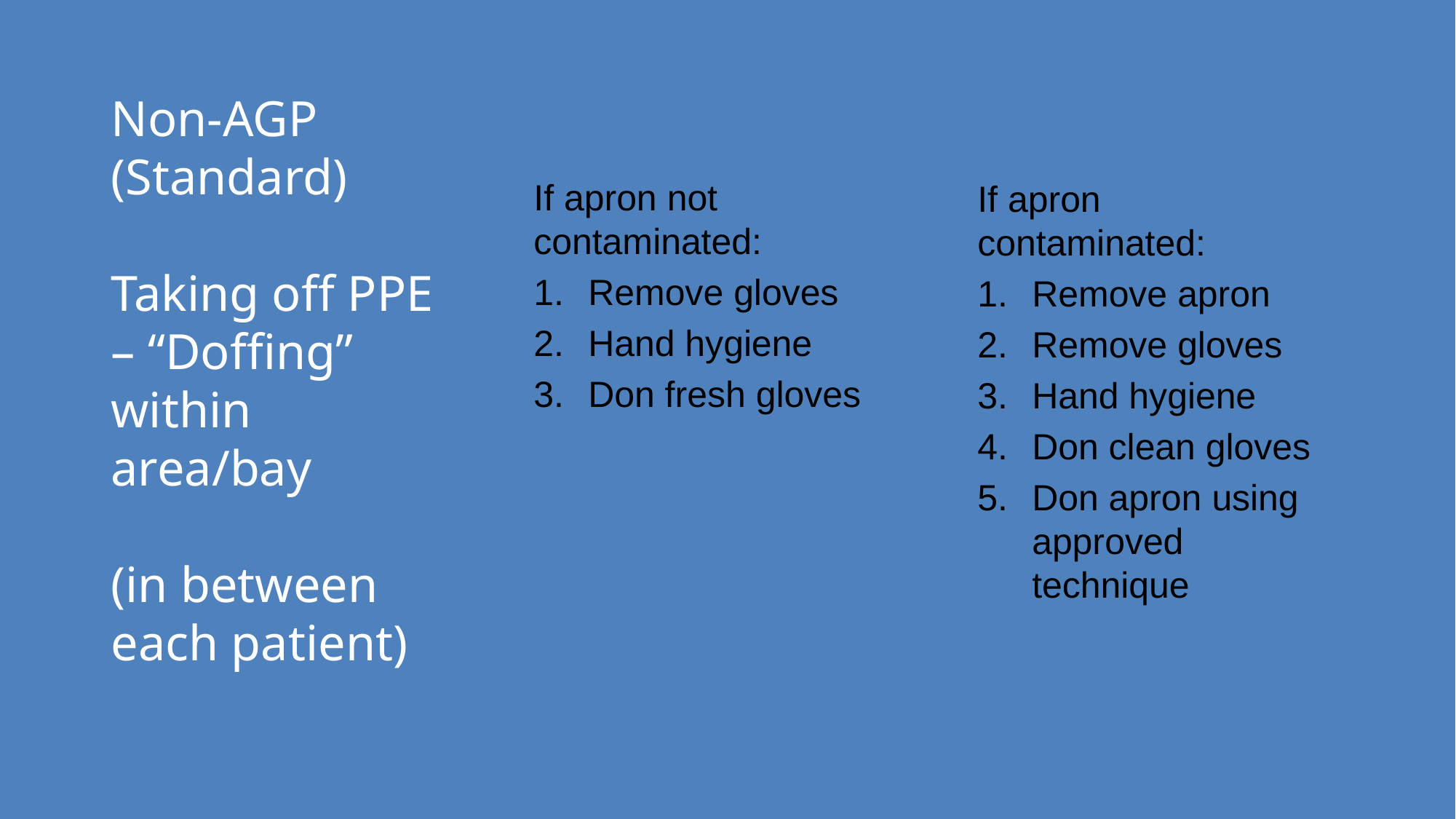

# Non-AGP (Standard) Taking off PPE – “Doffing” within area/bay (in between each patient)
If apron not contaminated:
Remove gloves
Hand hygiene
Don fresh gloves
If apron contaminated:
Remove apron
Remove gloves
Hand hygiene
Don clean gloves
Don apron using approved technique

## Slide 66
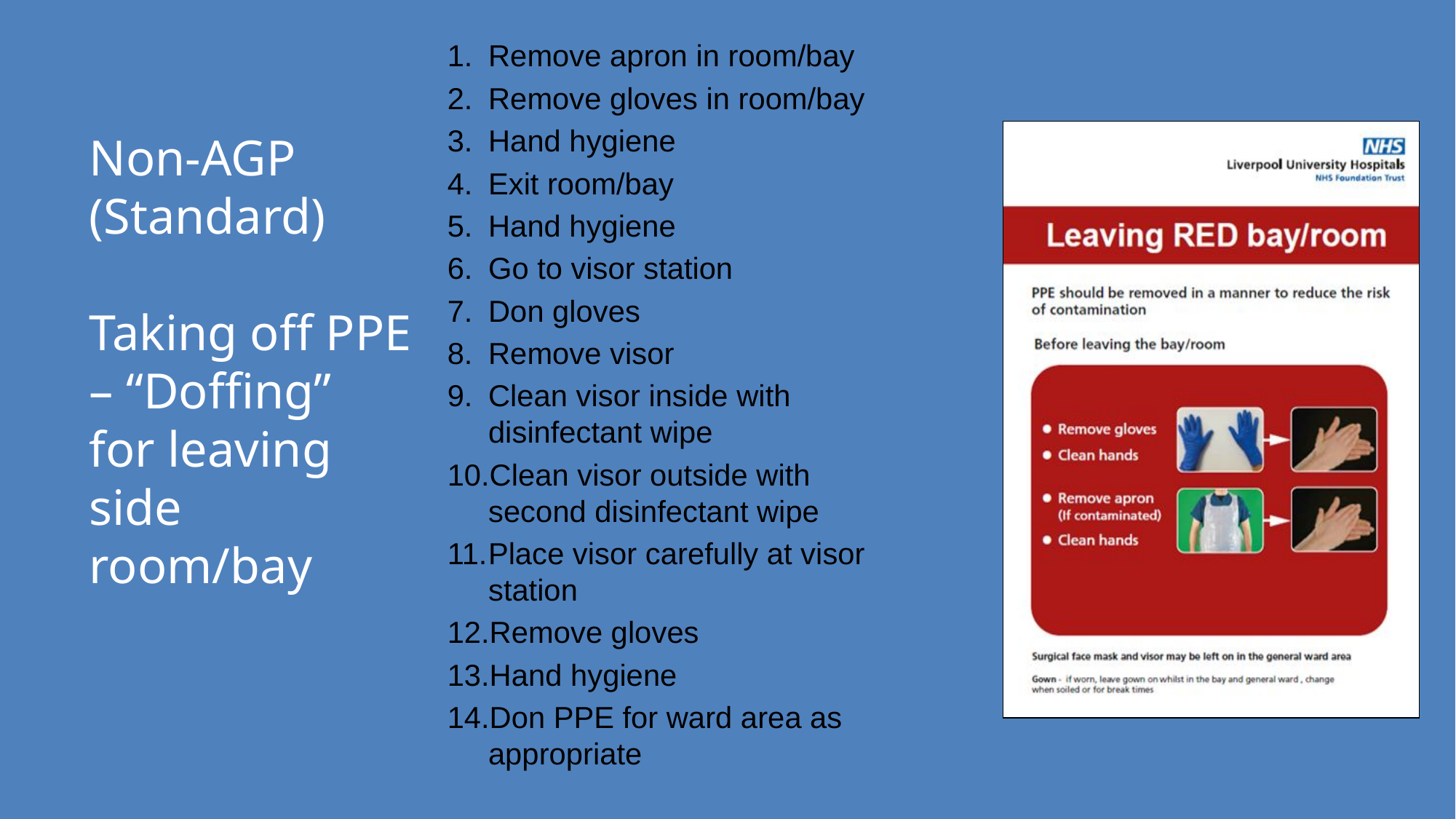

Remove apron in room/bay
Remove gloves in room/bay
Hand hygiene
Exit room/bay
Hand hygiene
Go to visor station
Don gloves
Remove visor
Clean visor inside with disinfectant wipe
Clean visor outside with second disinfectant wipe
Place visor carefully at visor station
Remove gloves
Hand hygiene
Don PPE for ward area as appropriate
# Non-AGP (Standard) Taking off PPE – “Doffing” for leaving side room/bay

## Slide 67
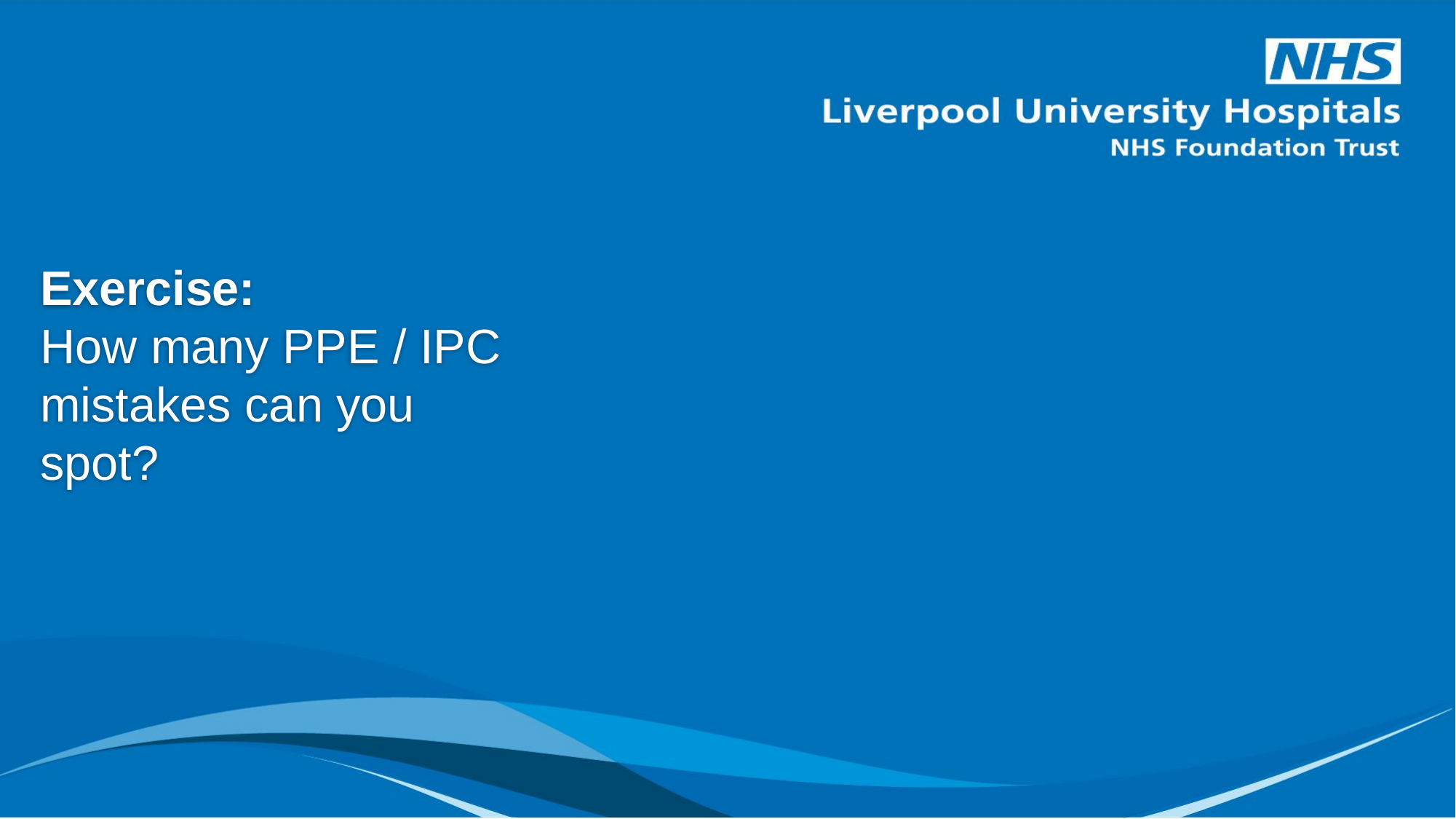

Exercise:
How many PPE / IPC mistakes can you spot?

## Slide 68
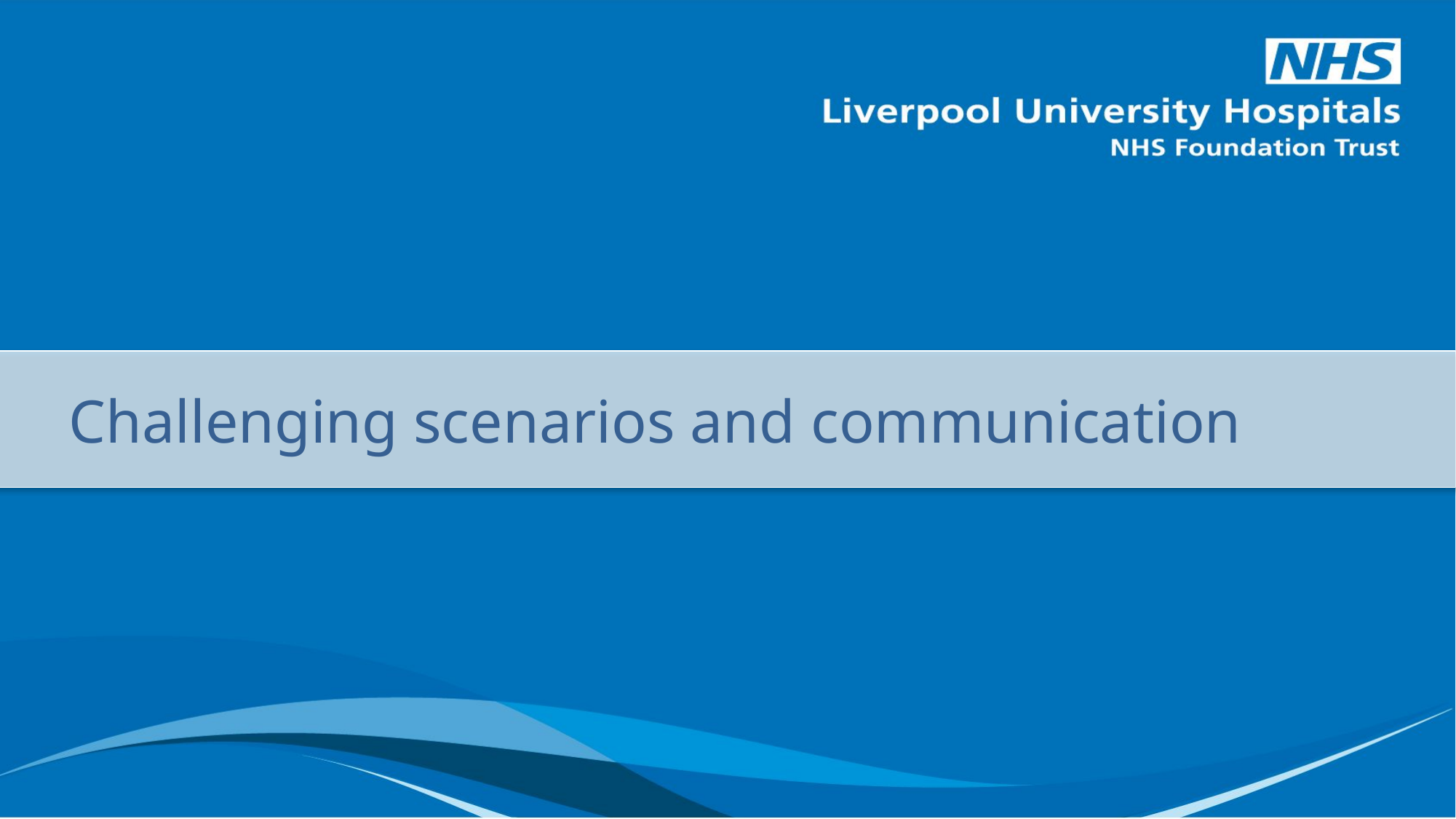

# Challenging scenarios and communication

## Slide 69
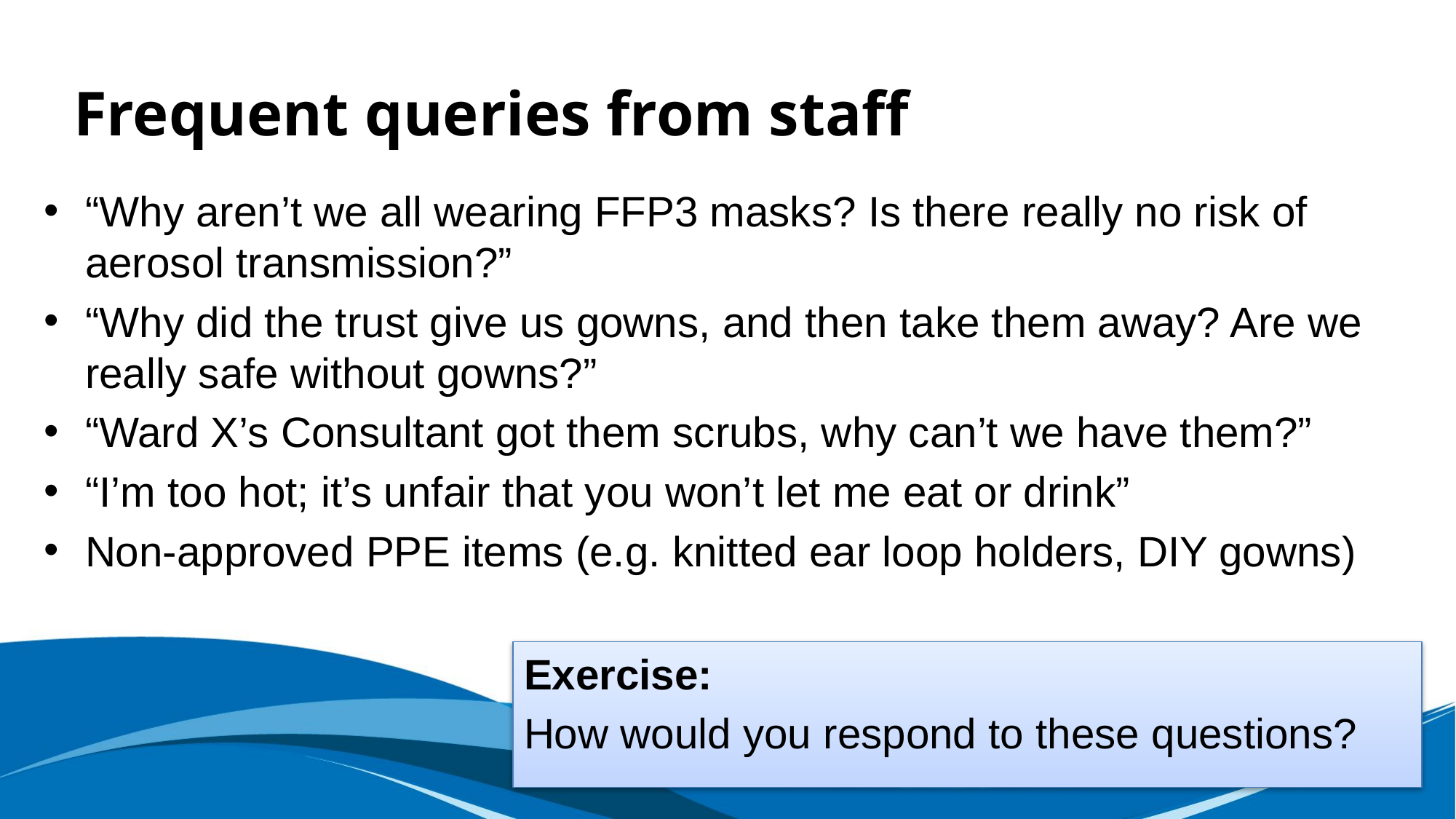

Frequent queries from staff
“Why aren’t we all wearing FFP3 masks? Is there really no risk of aerosol transmission?”
“Why did the trust give us gowns, and then take them away? Are we really safe without gowns?”
“Ward X’s Consultant got them scrubs, why can’t we have them?”
“I’m too hot; it’s unfair that you won’t let me eat or drink”
Non-approved PPE items (e.g. knitted ear loop holders, DIY gowns)
Exercise:
How would you respond to these questions?

## Slide 70
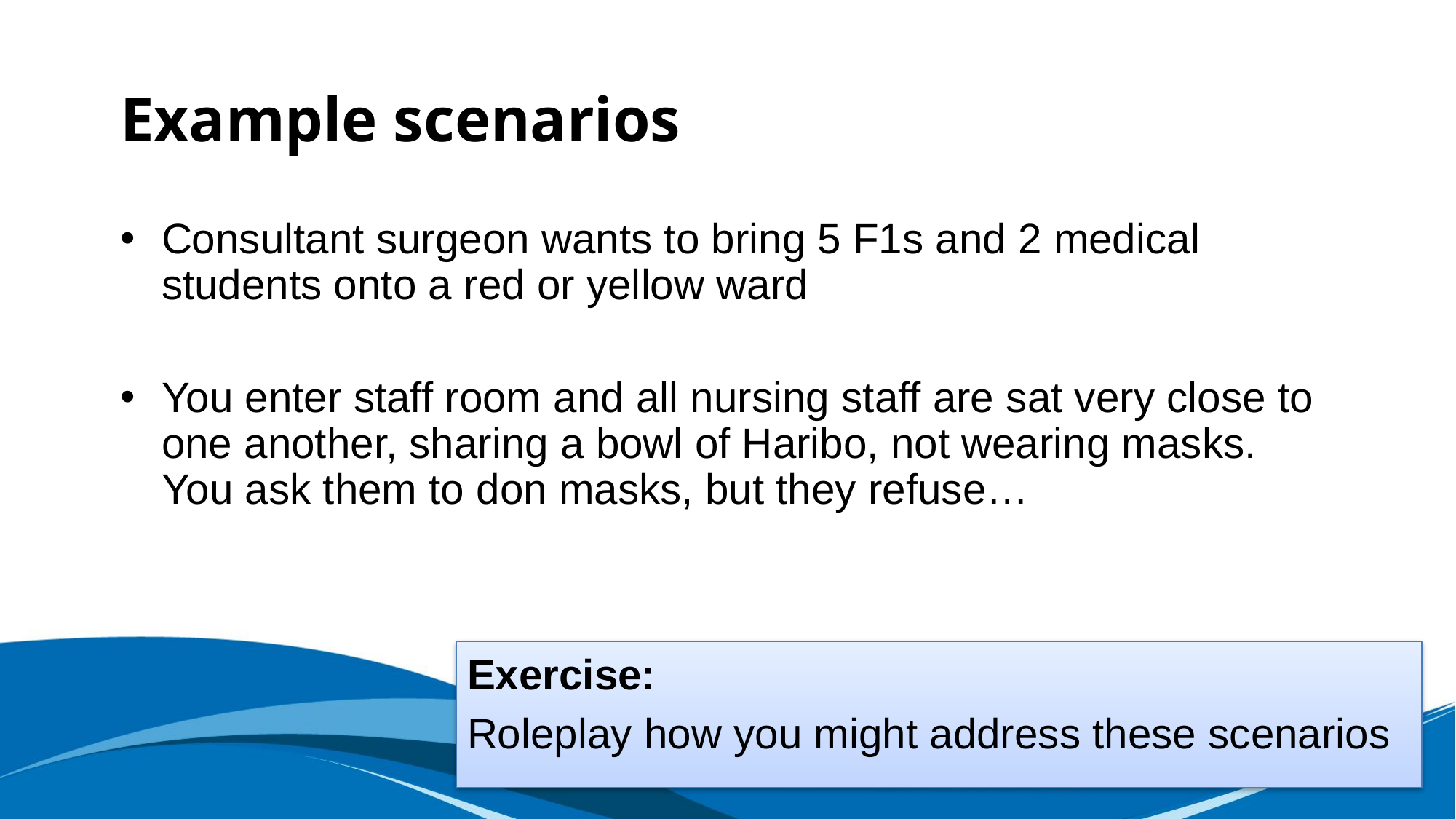

Example scenarios
Consultant surgeon wants to bring 5 F1s and 2 medical students onto a red or yellow ward
You enter staff room and all nursing staff are sat very close to one another, sharing a bowl of Haribo, not wearing masks. You ask them to don masks, but they refuse…
Exercise:
Roleplay how you might address these scenarios

## Slide 71
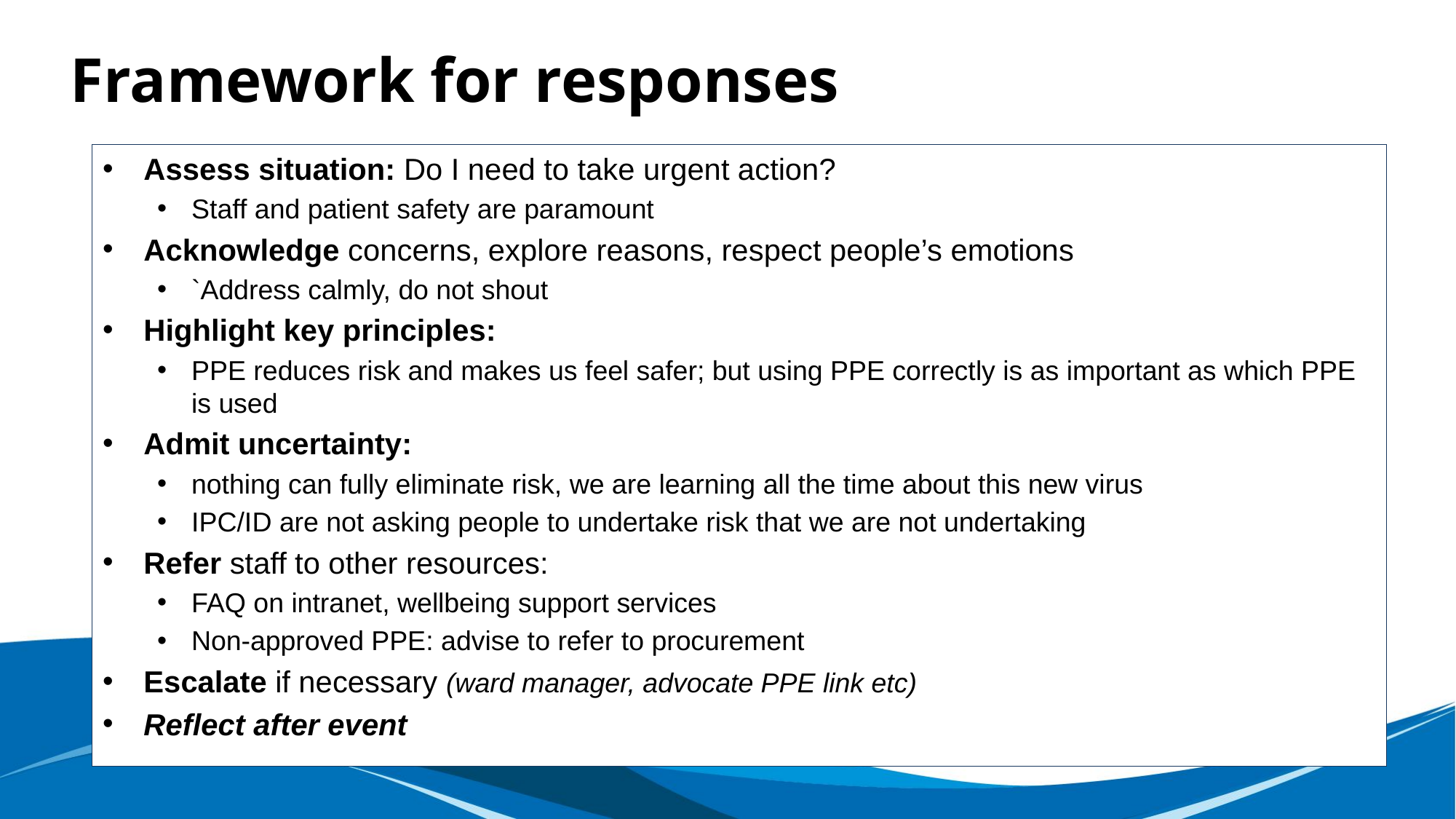

Framework for responses
Assess situation: Do I need to take urgent action?
Staff and patient safety are paramount
Acknowledge concerns, explore reasons, respect people’s emotions
`Address calmly, do not shout
Highlight key principles:
PPE reduces risk and makes us feel safer; but using PPE correctly is as important as which PPE is used
Admit uncertainty:
nothing can fully eliminate risk, we are learning all the time about this new virus
IPC/ID are not asking people to undertake risk that we are not undertaking
Refer staff to other resources:
FAQ on intranet, wellbeing support services
Non-approved PPE: advise to refer to procurement
Escalate if necessary (ward manager, advocate PPE link etc)
Reflect after event

## Slide 72
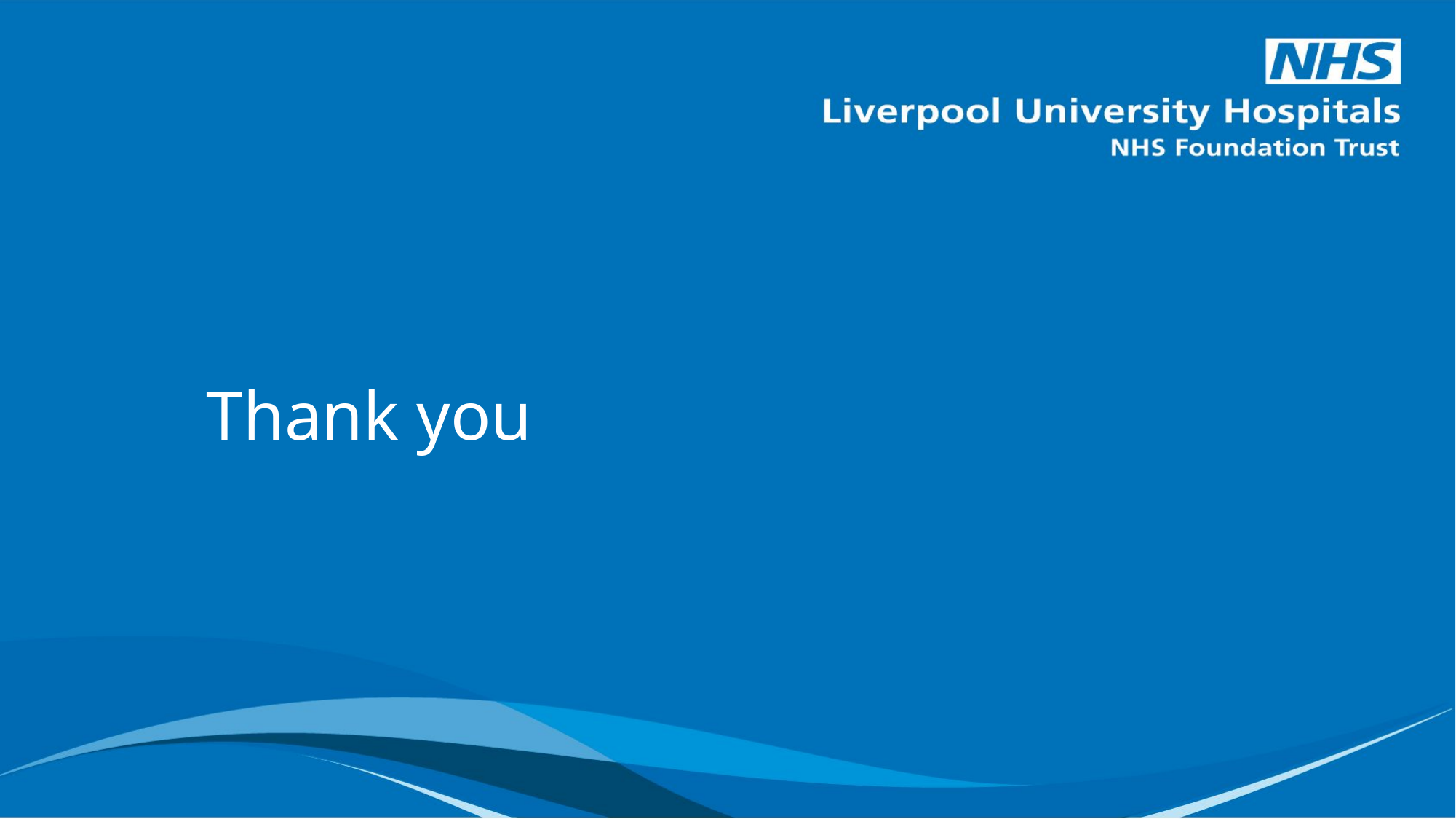

# Thank you

## Slide 73
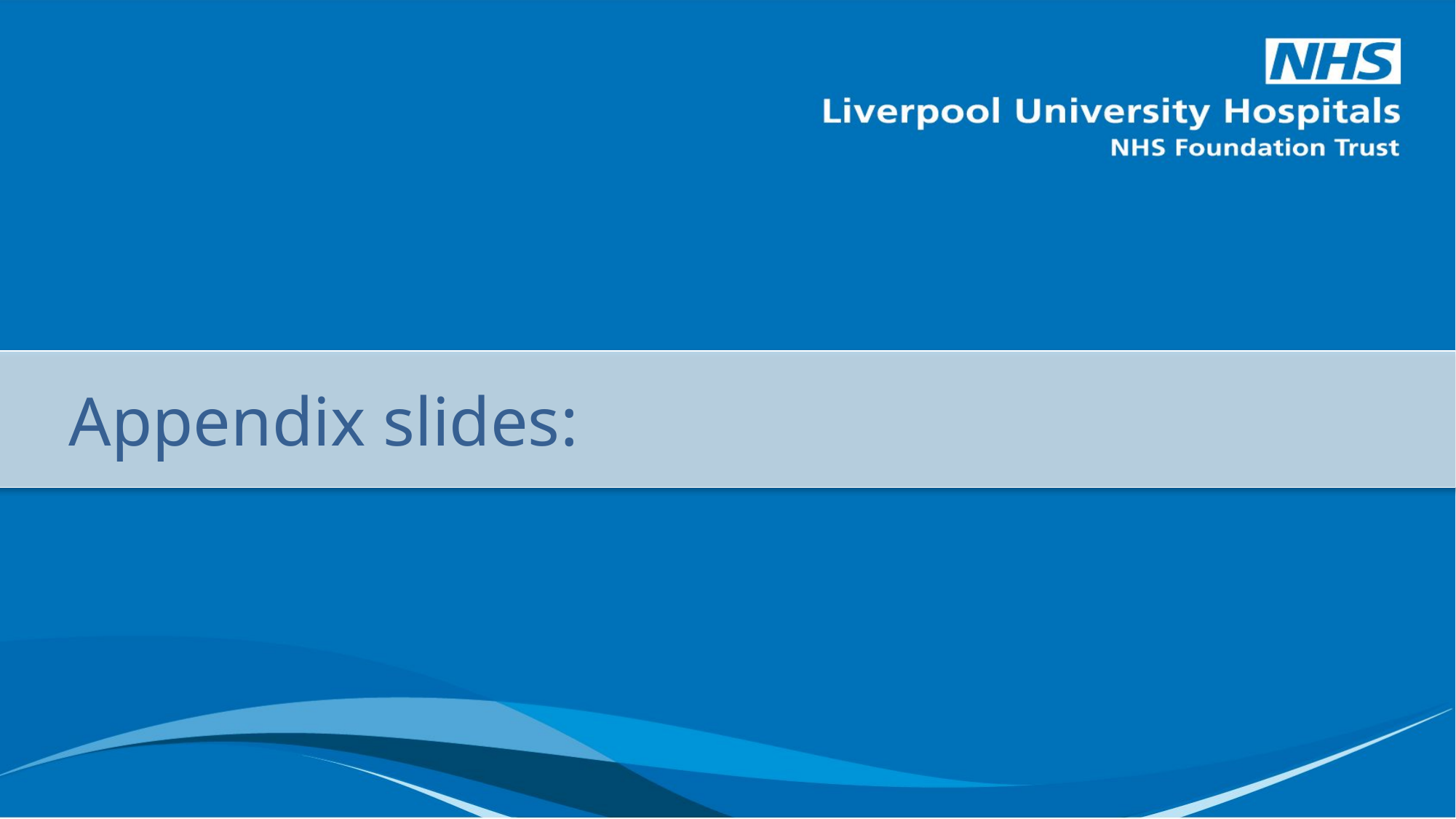

# Appendix slides:

## Slide 74
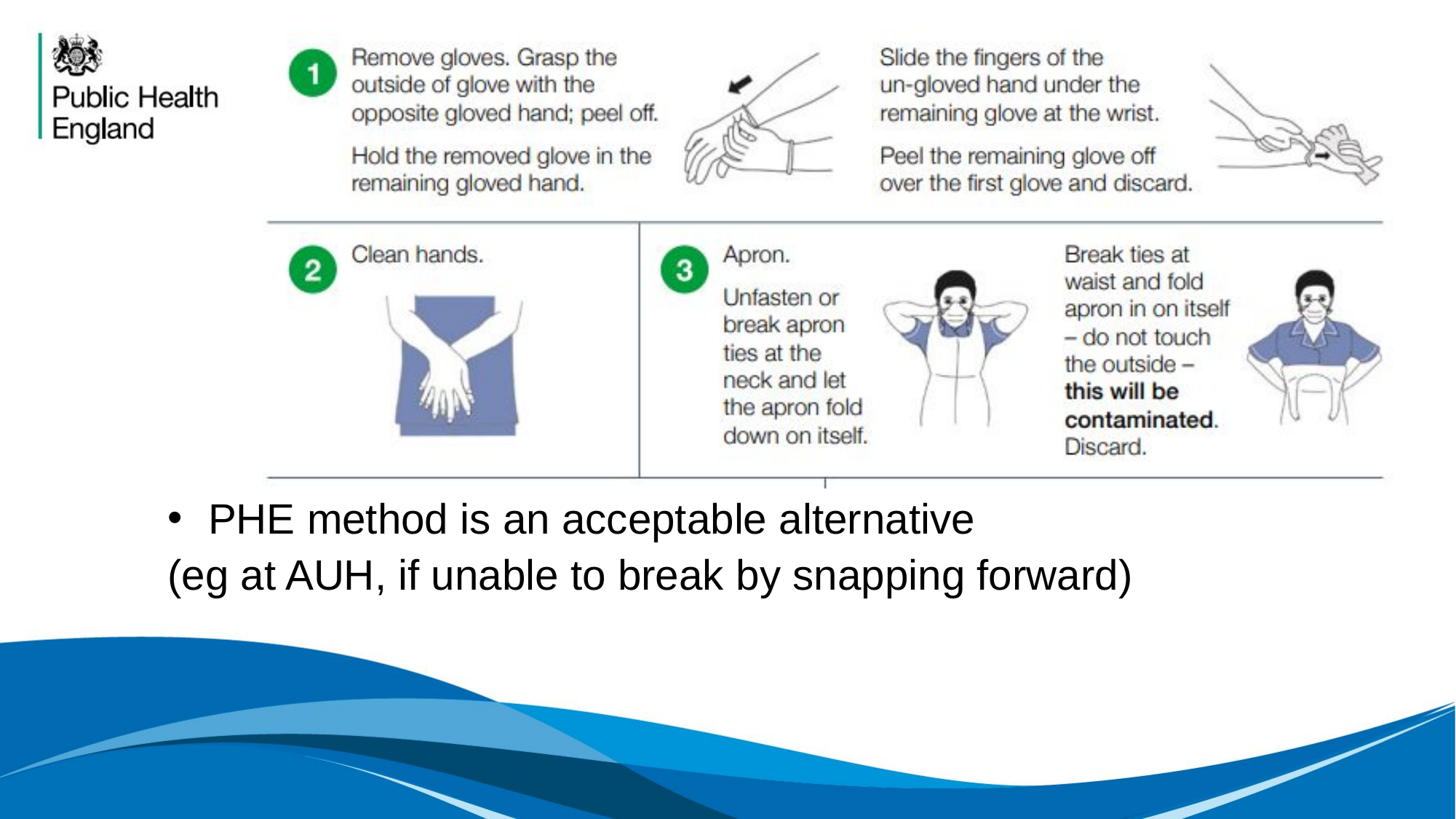

PHE method is an acceptable alternative
(eg at AUH, if unable to break by snapping forward)

## Slide 75
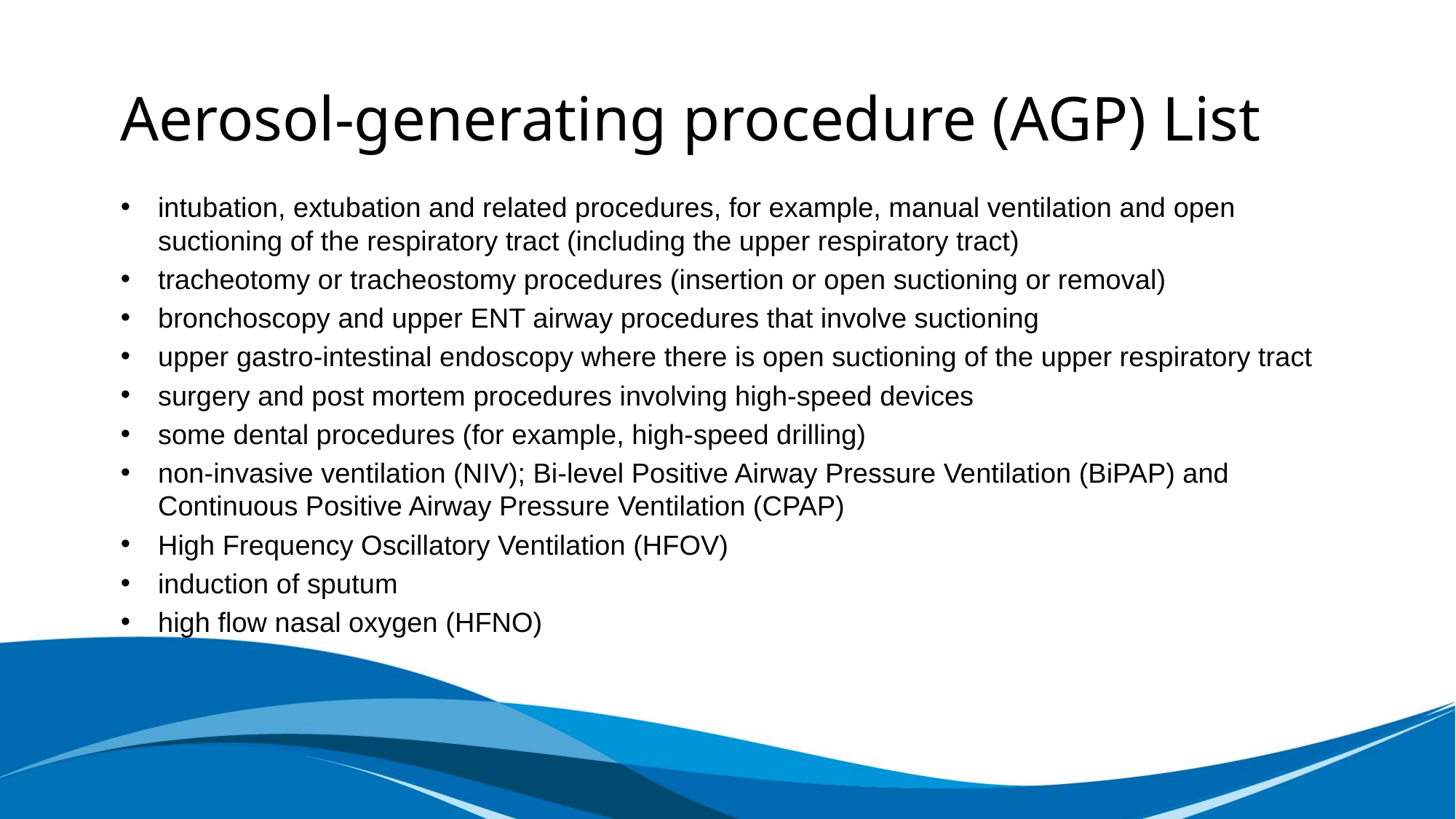

Aerosol-generating procedure (AGP) List
intubation, extubation and related procedures, for example, manual ventilation and open suctioning of the respiratory tract (including the upper respiratory tract)
tracheotomy or tracheostomy procedures (insertion or open suctioning or removal)
bronchoscopy and upper ENT airway procedures that involve suctioning
upper gastro-intestinal endoscopy where there is open suctioning of the upper respiratory tract
surgery and post mortem procedures involving high-speed devices
some dental procedures (for example, high-speed drilling)
non-invasive ventilation (NIV); Bi-level Positive Airway Pressure Ventilation (BiPAP) and Continuous Positive Airway Pressure Ventilation (CPAP)
High Frequency Oscillatory Ventilation (HFOV)
induction of sputum
high flow nasal oxygen (HFNO)

## Slide 76
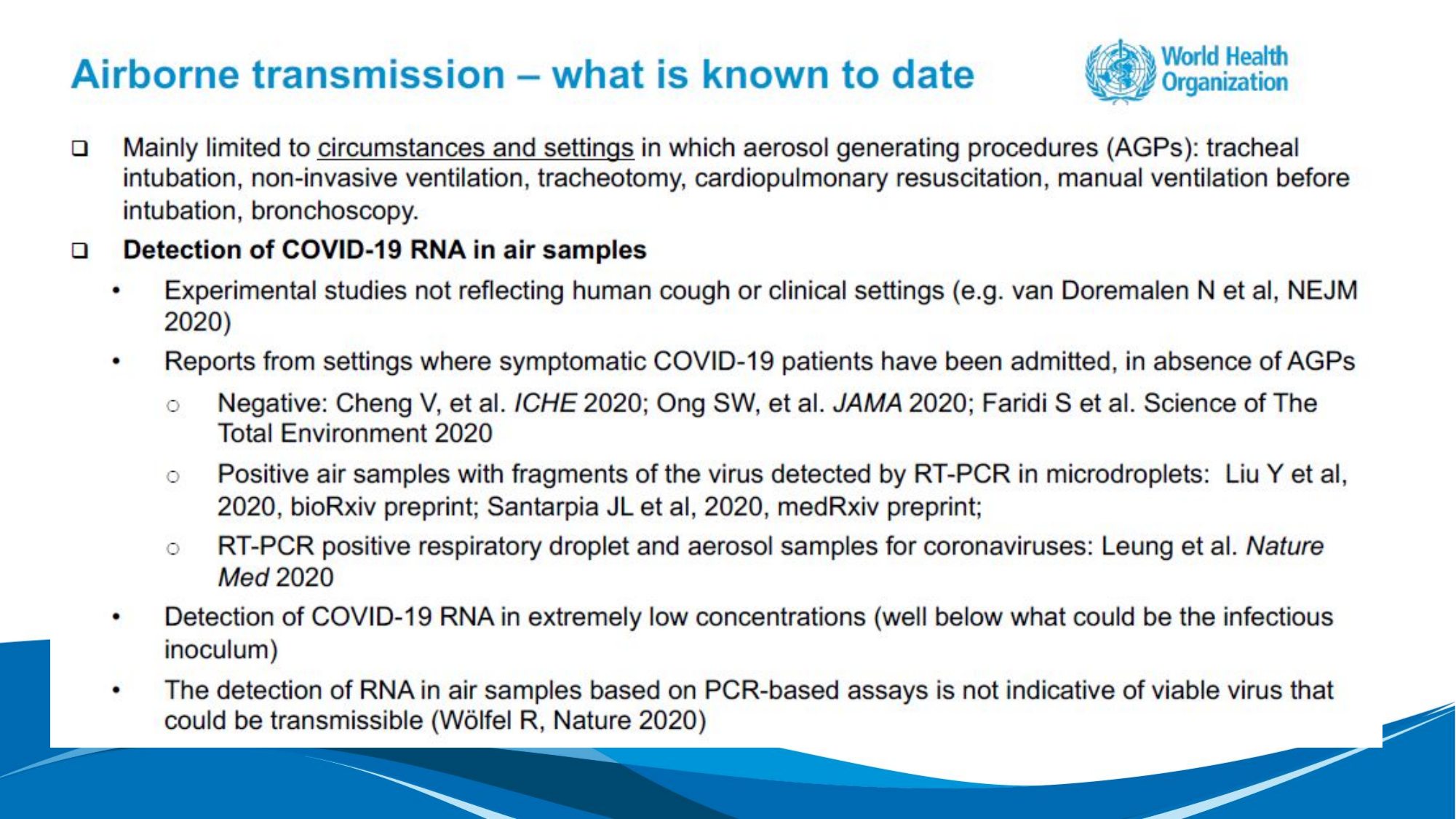

## Slide 77
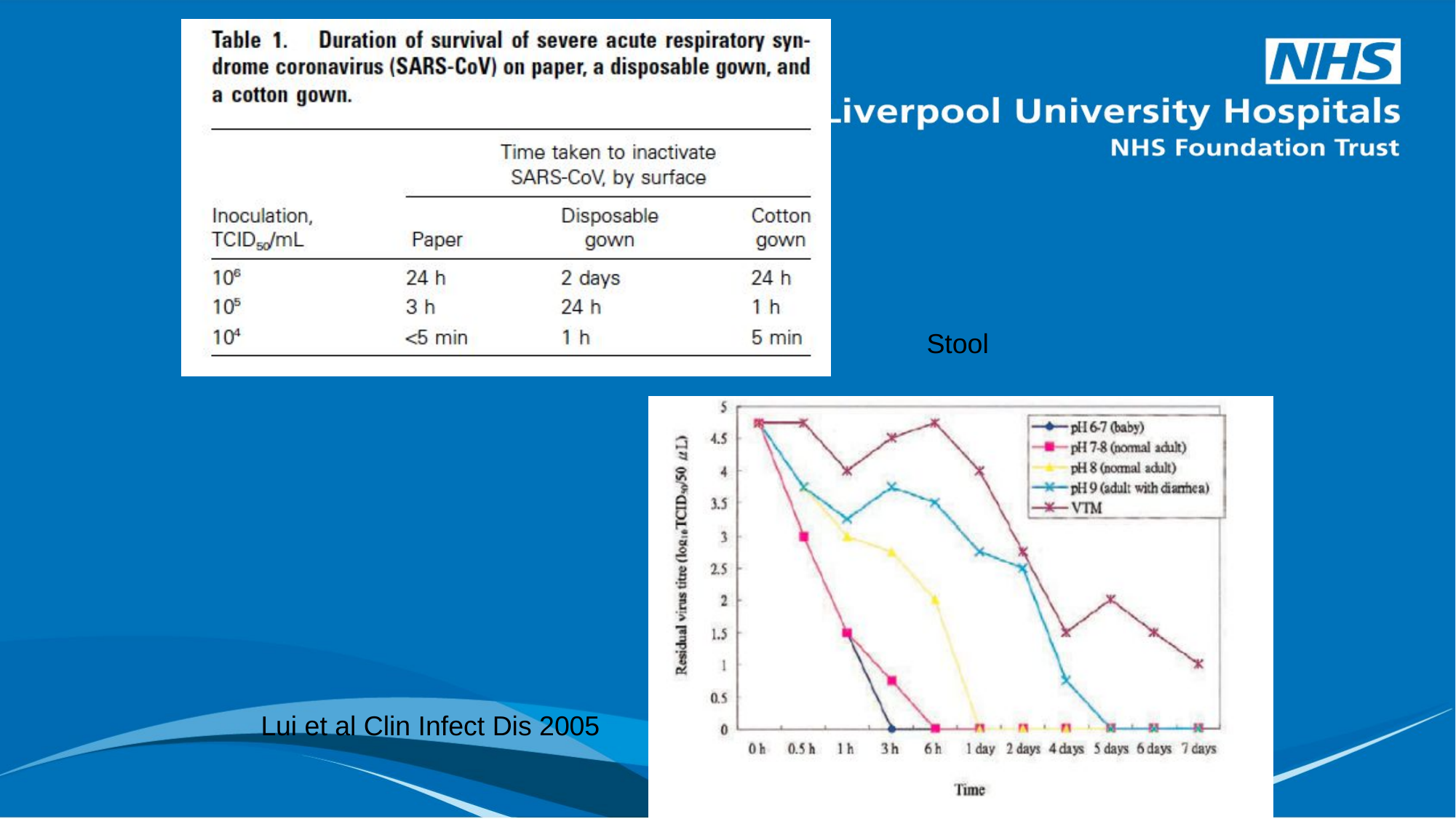

Stool
Lui et al Clin Infect Dis 2005

## Slide 78
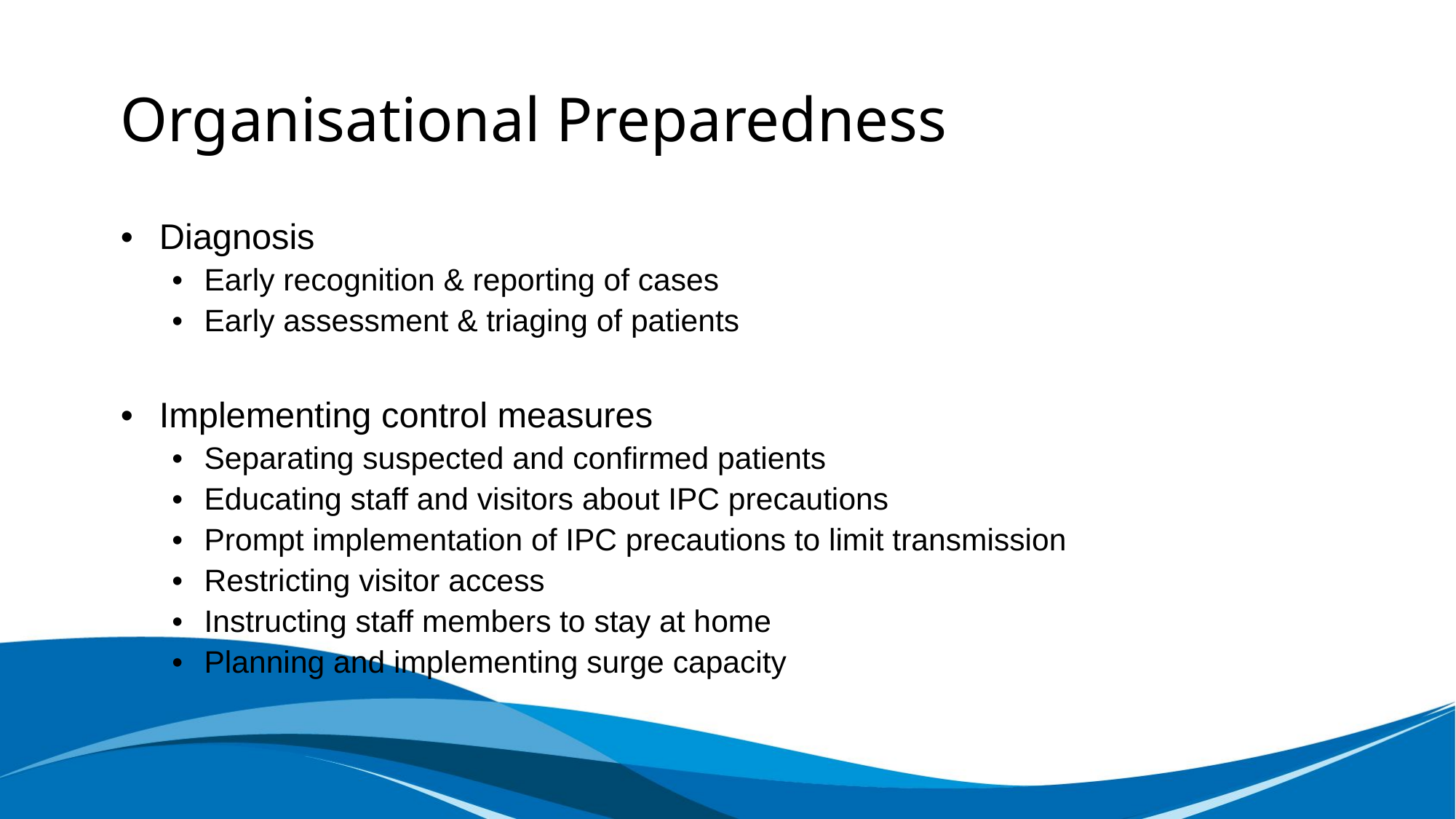

Organisational Preparedness
Diagnosis
Early recognition & reporting of cases
Early assessment & triaging of patients
Implementing control measures
Separating suspected and confirmed patients
Educating staff and visitors about IPC precautions
Prompt implementation of IPC precautions to limit transmission
Restricting visitor access
Instructing staff members to stay at home
Planning and implementing surge capacity

## Slide 79
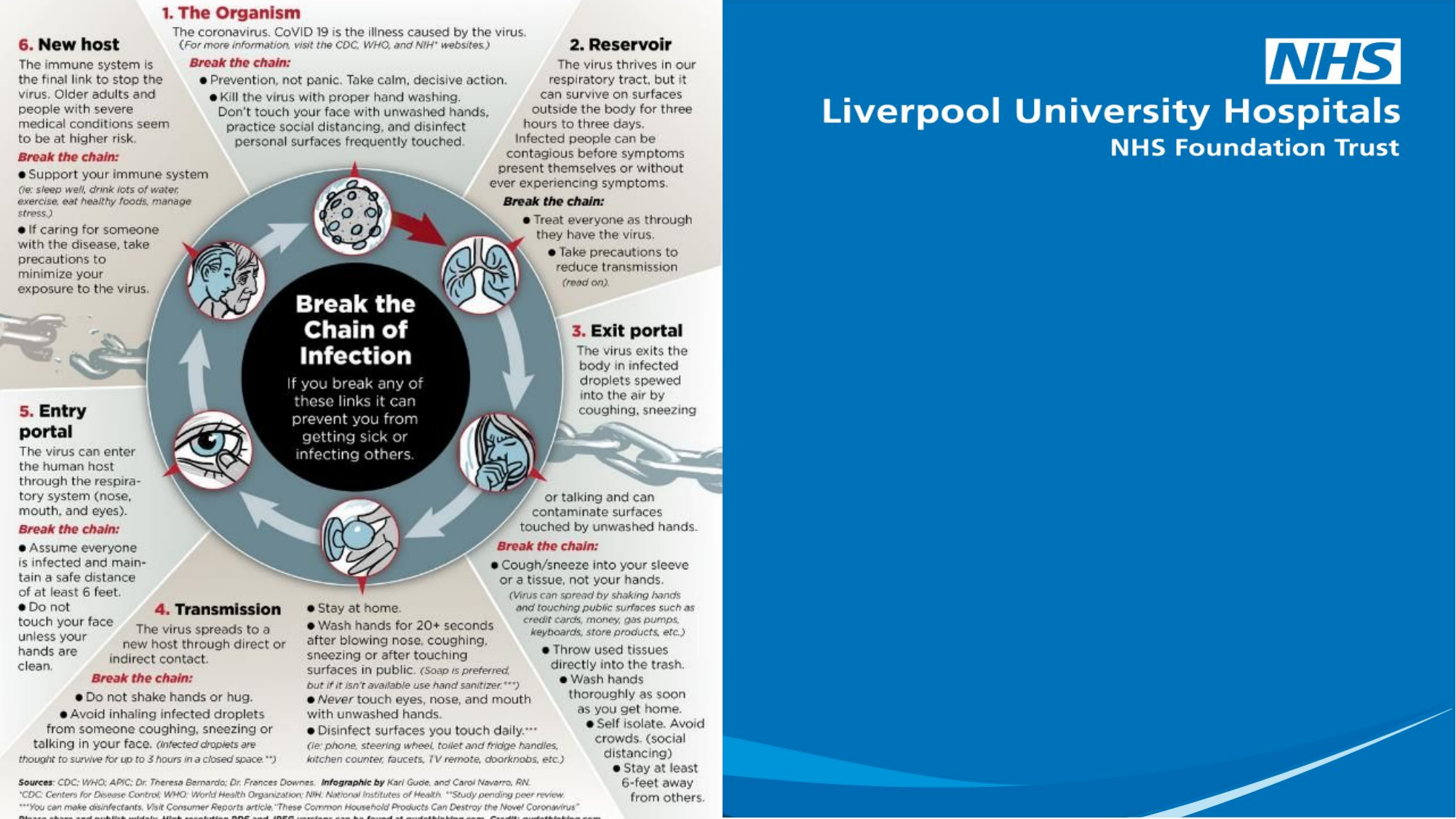

Supplement: Supplementary data 1 [file mmc1.pptx]
